# Supplementary figures and images for: Akkermansia muciniphila identified as key strain to alleviate gut barrier injury through Wnt signaling pathway
Source: eLife. 2025 Feb 6;12:RP92906. doi: 10.7554/eLife.92906 (PMC11801796; doi:10.7554/eLife.92906)

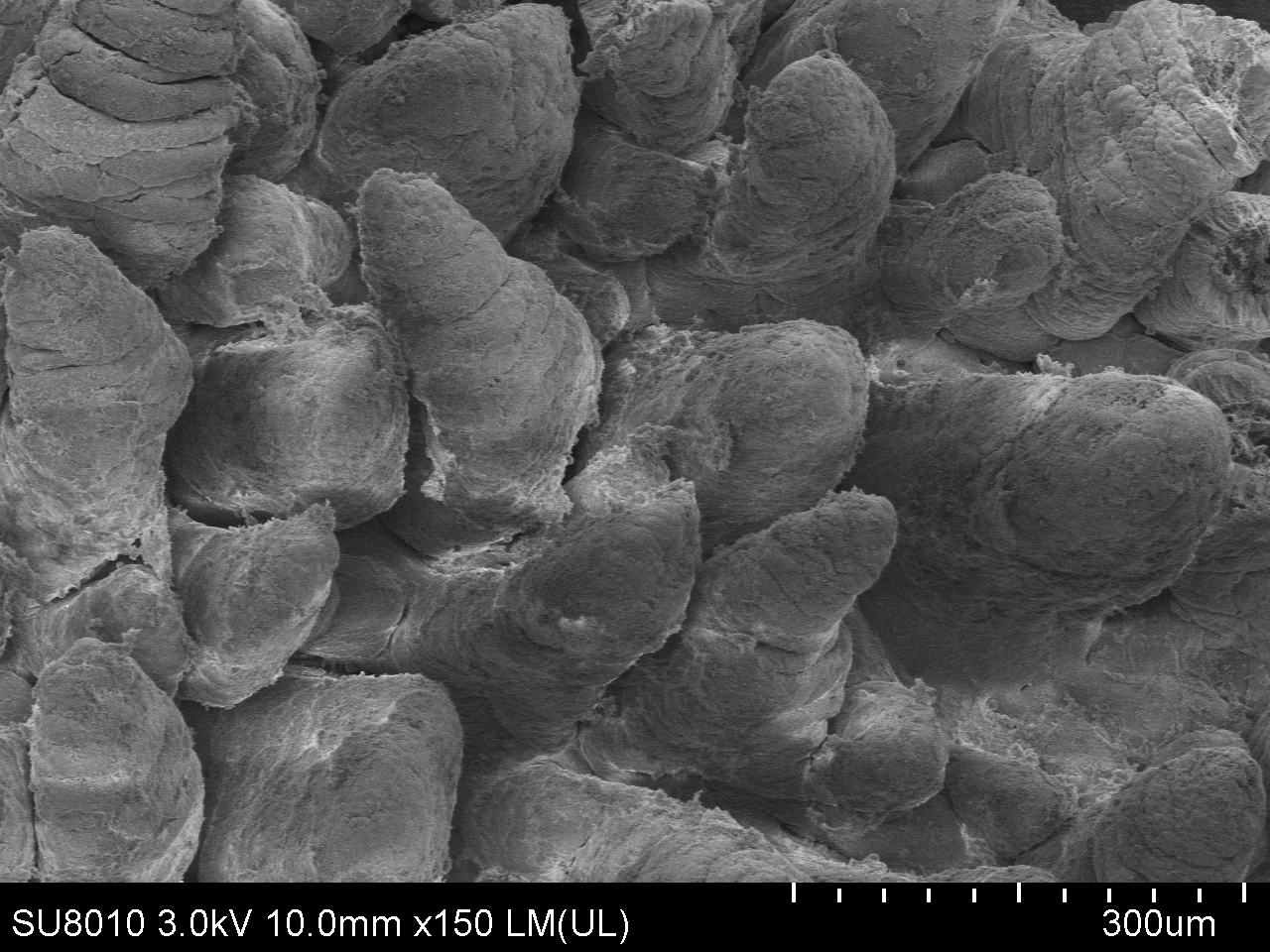

Supplement: Figure 3—source data 1. [file elife-92906-fig3-data1.zip › Figure 3-source data 1/Figure3-CON-SEM-150.tif]

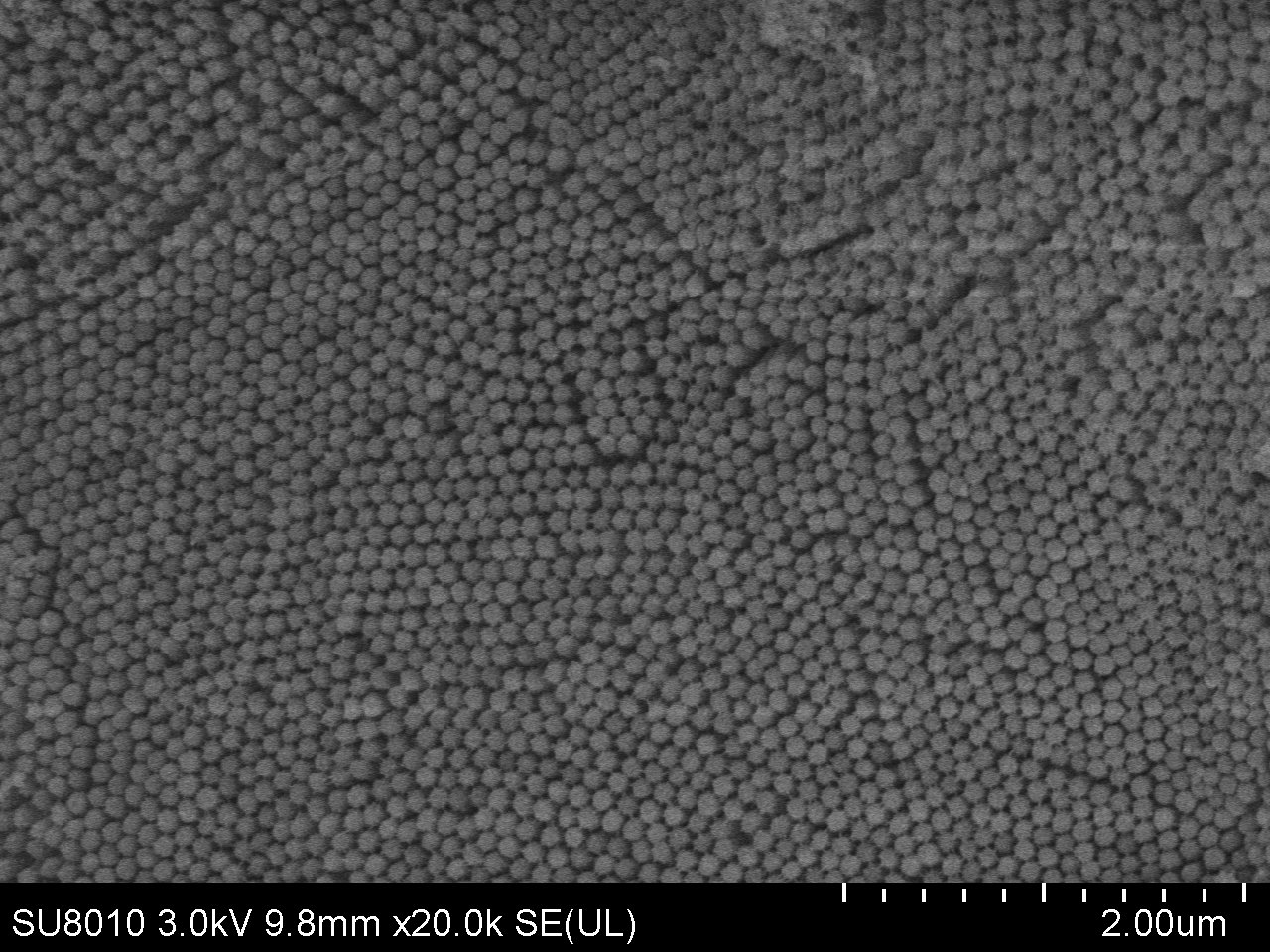

Supplement: Figure 3—source data 1. [file elife-92906-fig3-data1.zip › Figure 3-source data 1/Figure3-CON-SEM-20k.tif]

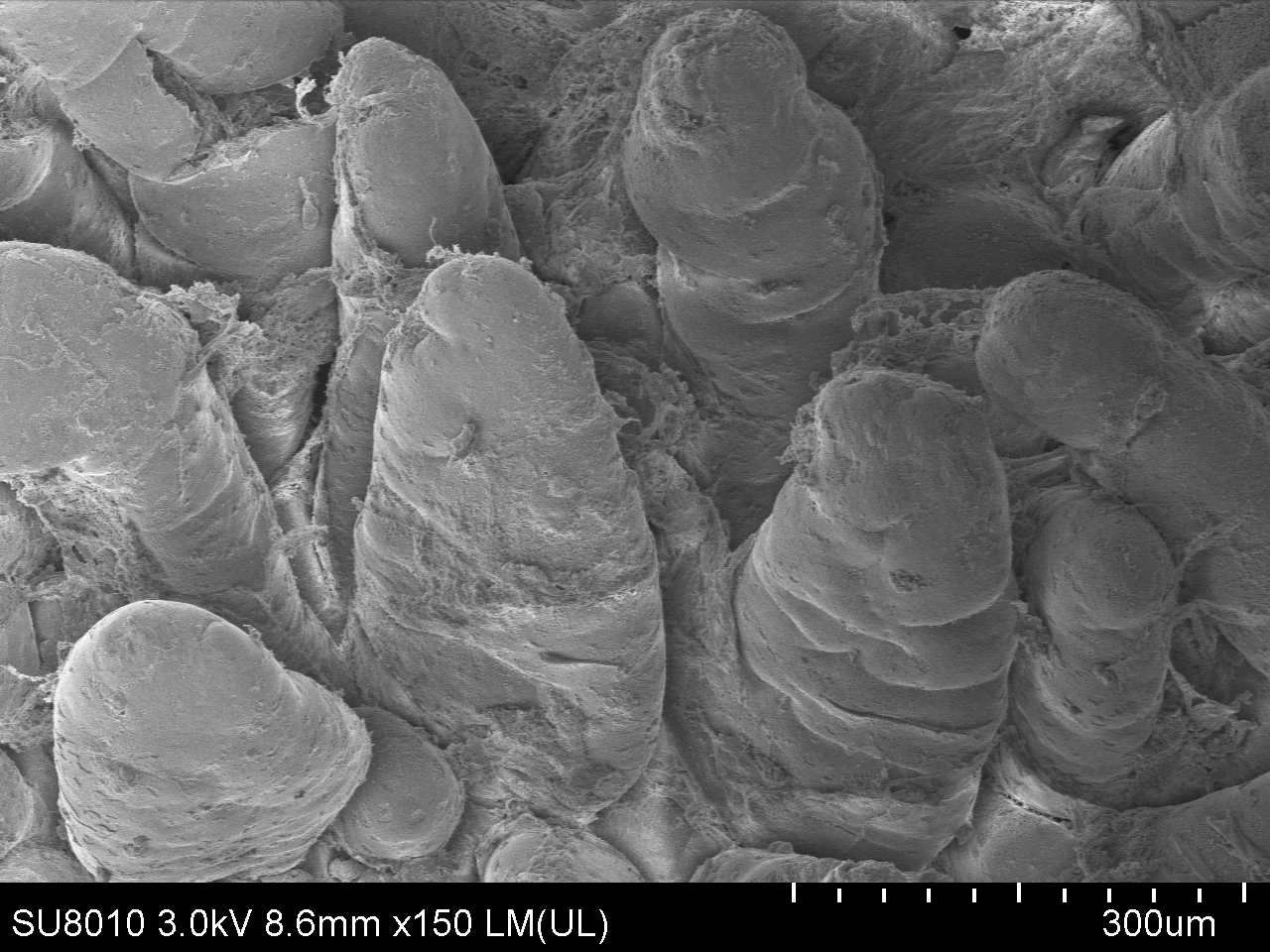

Supplement: Figure 3—source data 1. [file elife-92906-fig3-data1.zip › Figure 3-source data 1/Figure3-EF-SEM-150.tif]

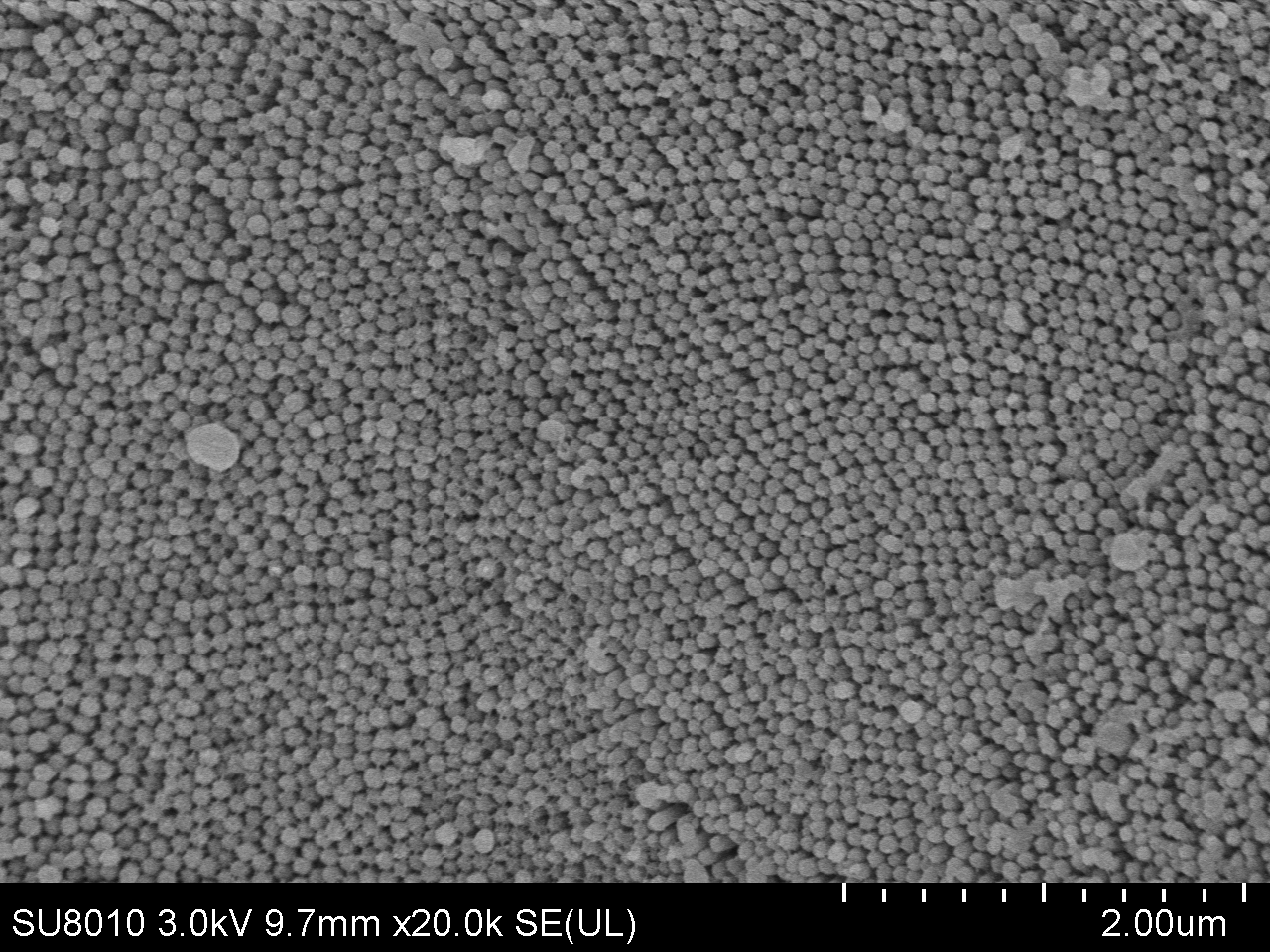

Supplement: Figure 3—source data 1. [file elife-92906-fig3-data1.zip › Figure 3-source data 1/Figure3-EF-SEM-20k.tif]

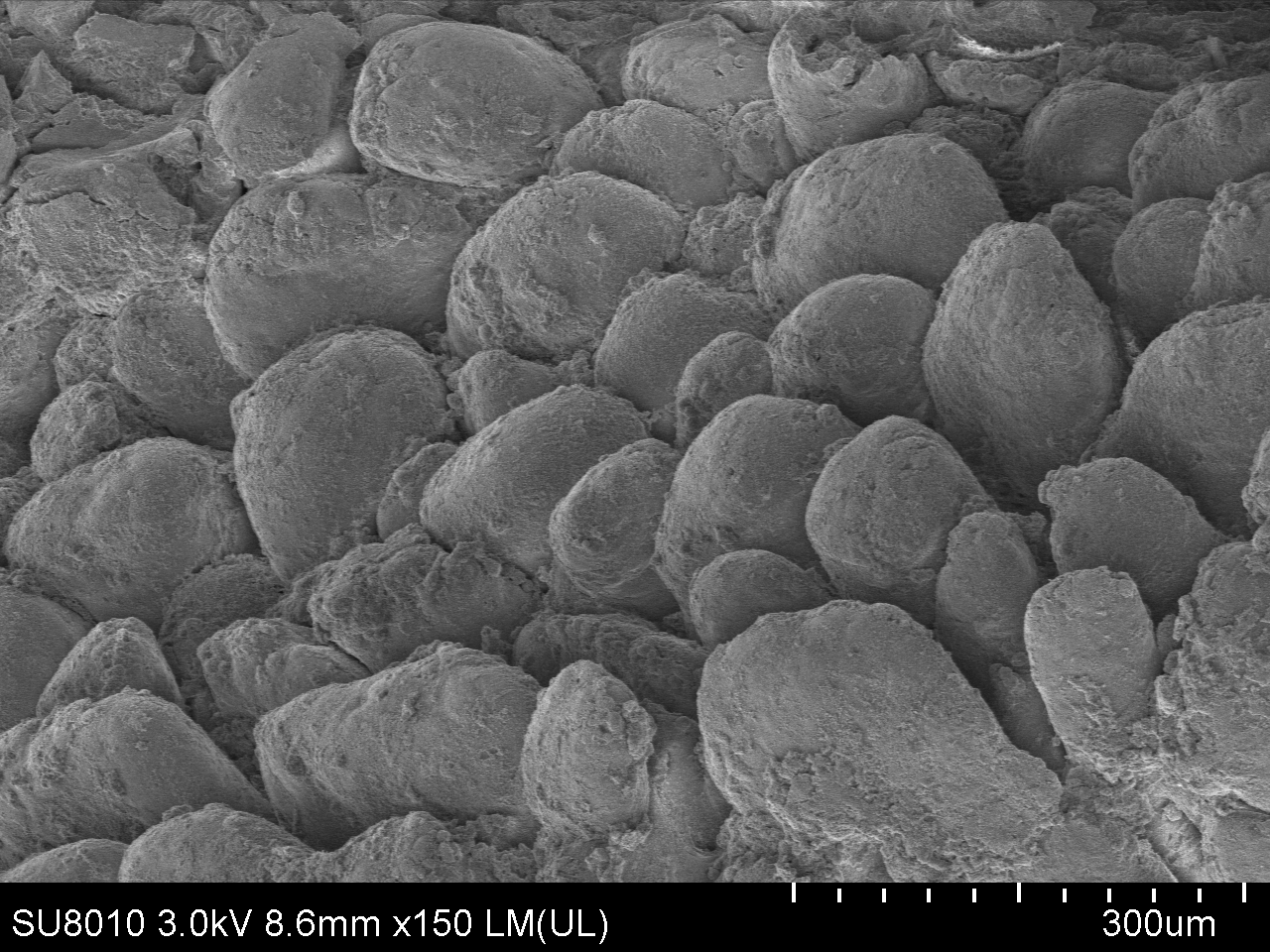

Supplement: Figure 3—source data 1. [file elife-92906-fig3-data1.zip › Figure 3-source data 1/Figure3-EP-SEM-150.tif]

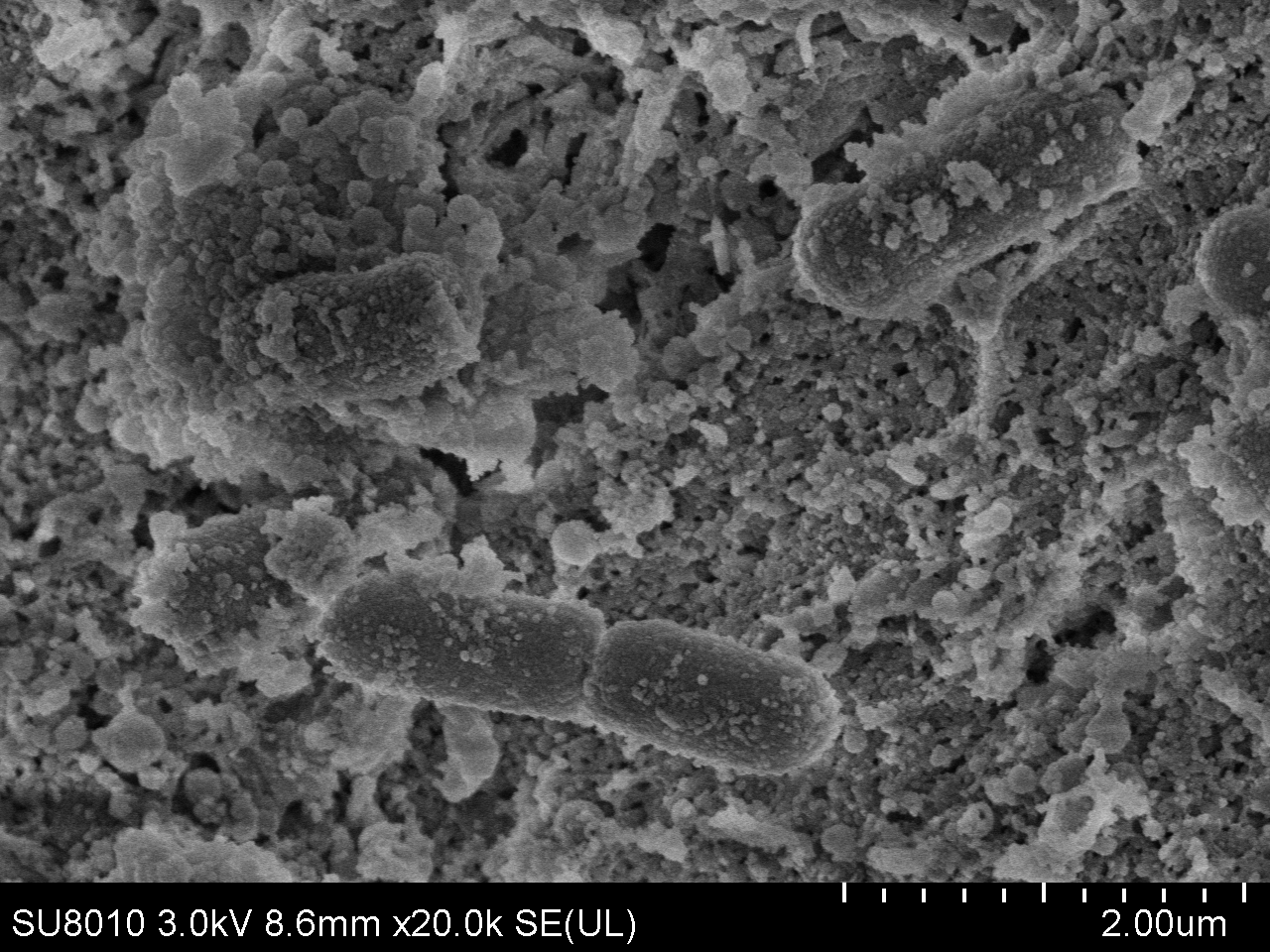

Supplement: Figure 3—source data 1. [file elife-92906-fig3-data1.zip › Figure 3-source data 1/Figure3-EP-SEM-20k.tif]

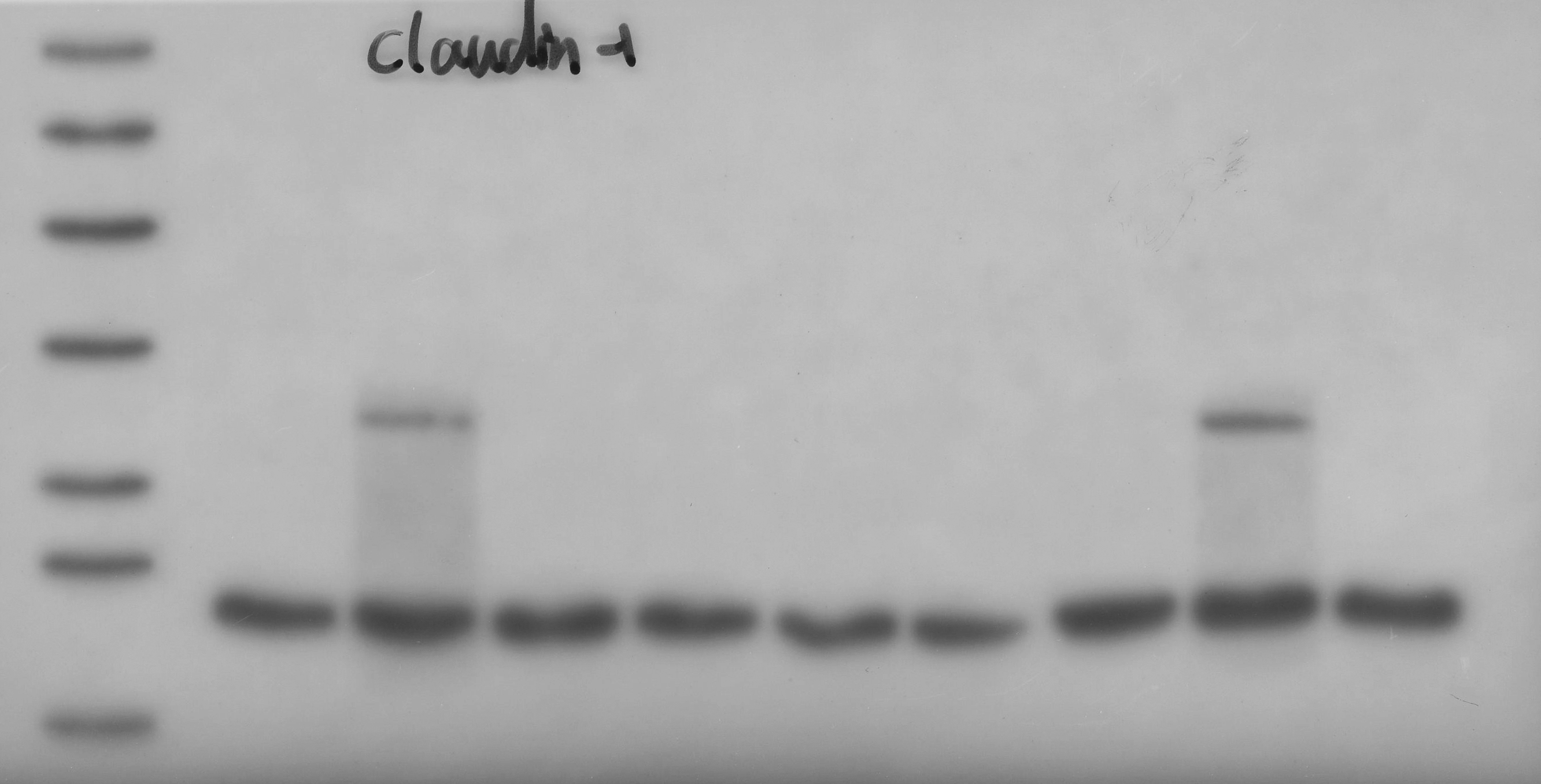

Supplement: Figure 3—source data 2. [file elife-92906-fig3-data2.zip › Figure 3-source data 2/Figure3-Claudin-1.tif]

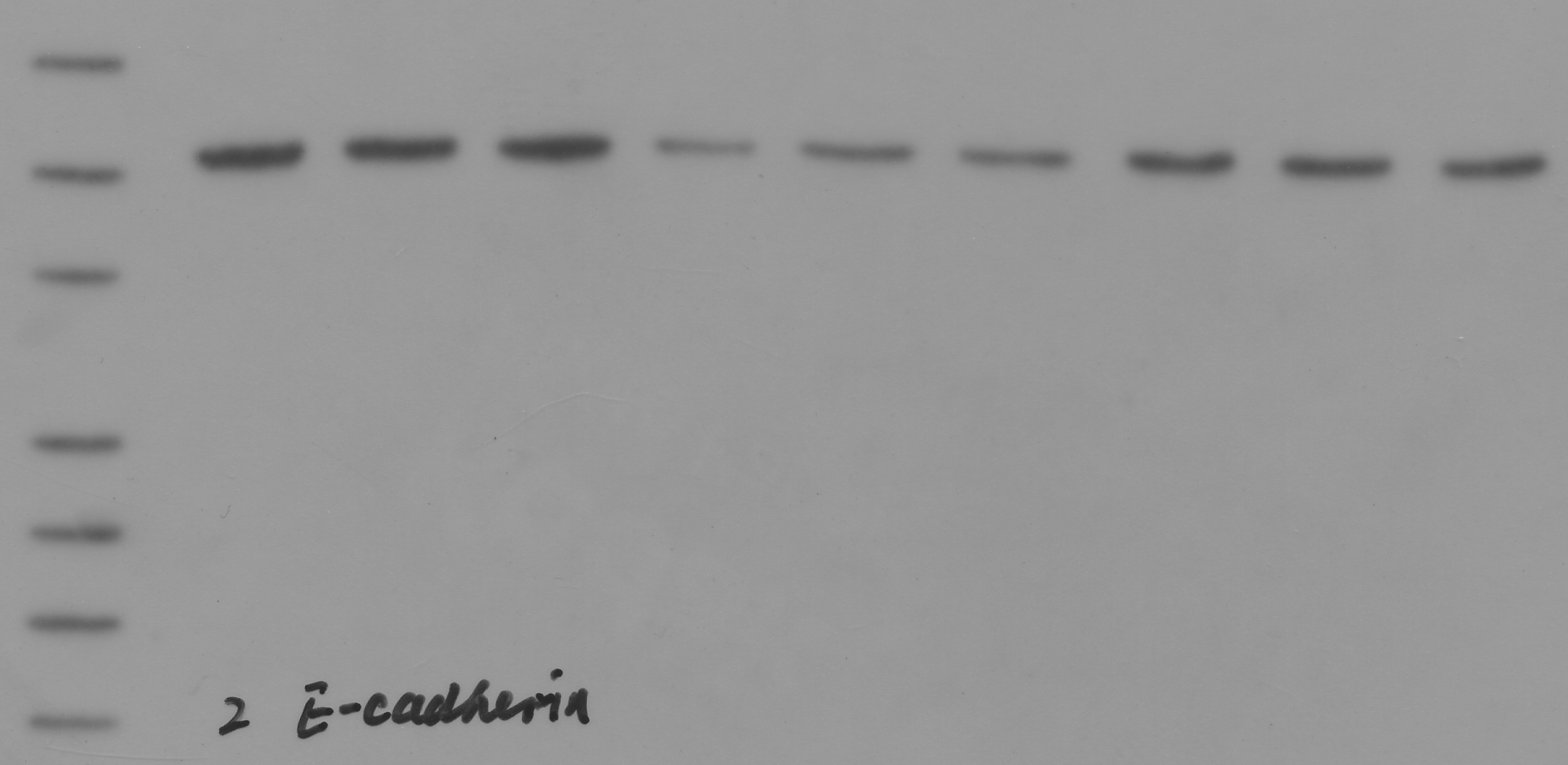

Supplement: Figure 3—source data 2. [file elife-92906-fig3-data2.zip › Figure 3-source data 2/Figure3-E-cadherin.tif]

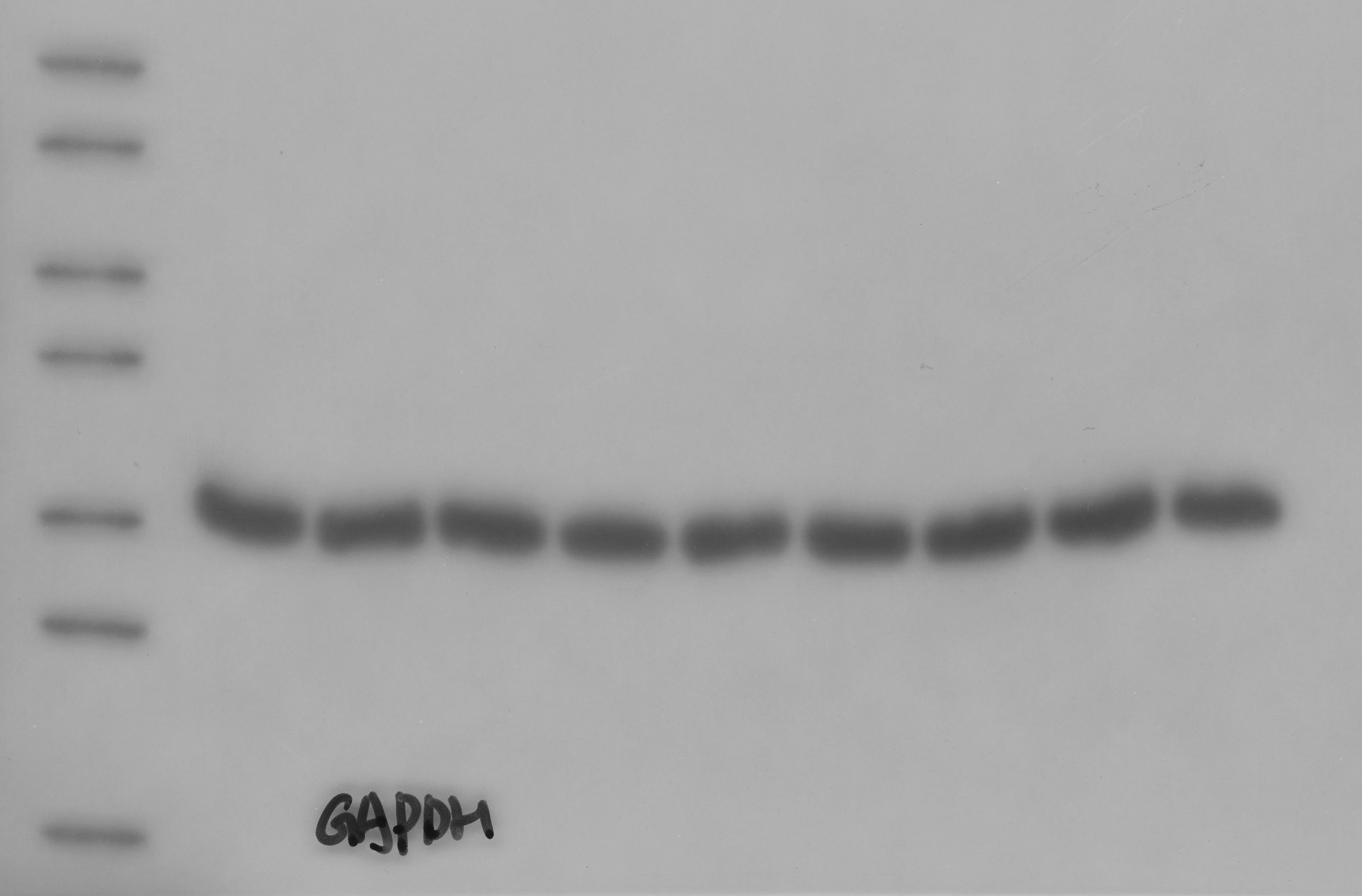

Supplement: Figure 3—source data 2. [file elife-92906-fig3-data2.zip › Figure 3-source data 2/Figure3-GAPDH-1.tif]

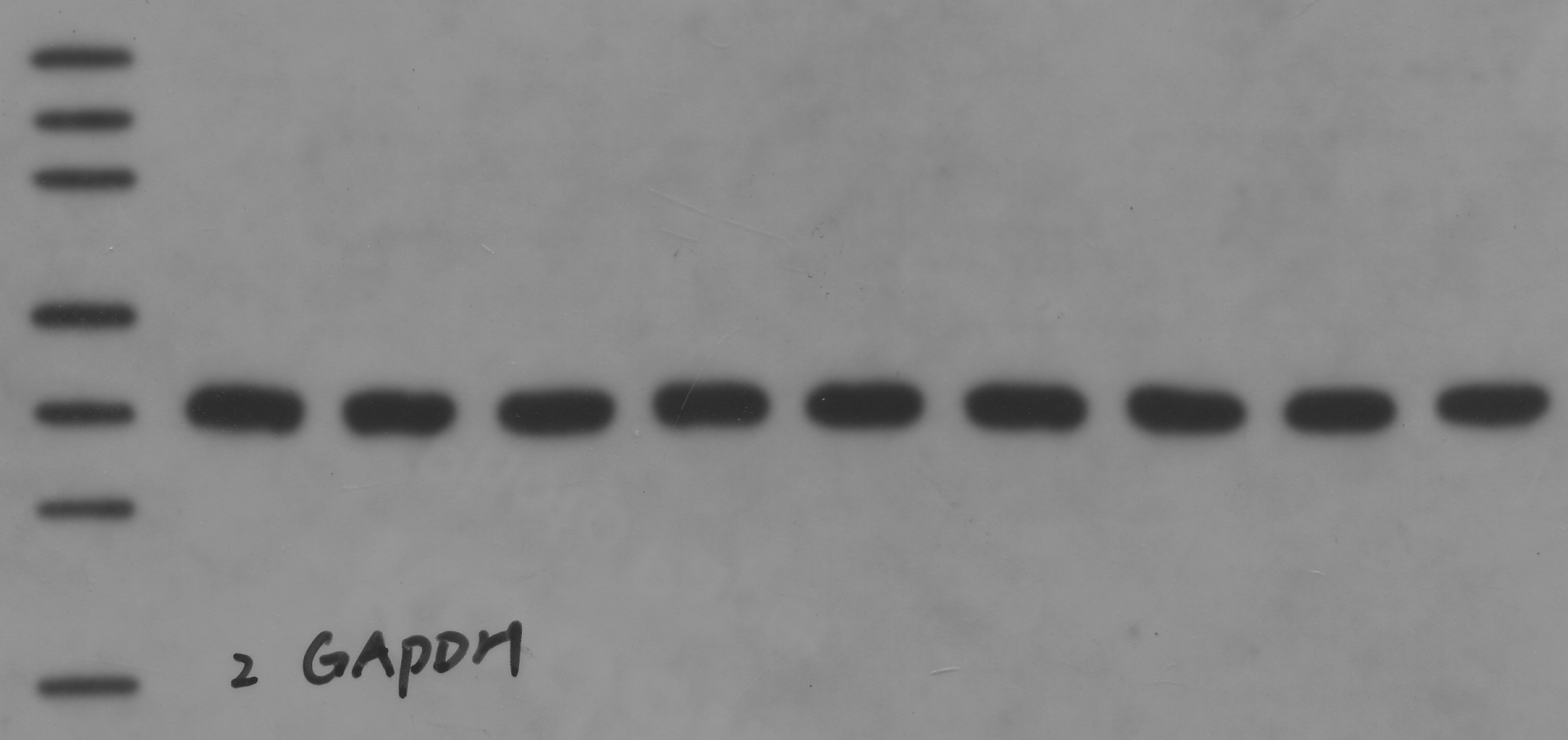

Supplement: Figure 3—source data 2. [file elife-92906-fig3-data2.zip › Figure 3-source data 2/Figure3-GAPDH-2.tif]

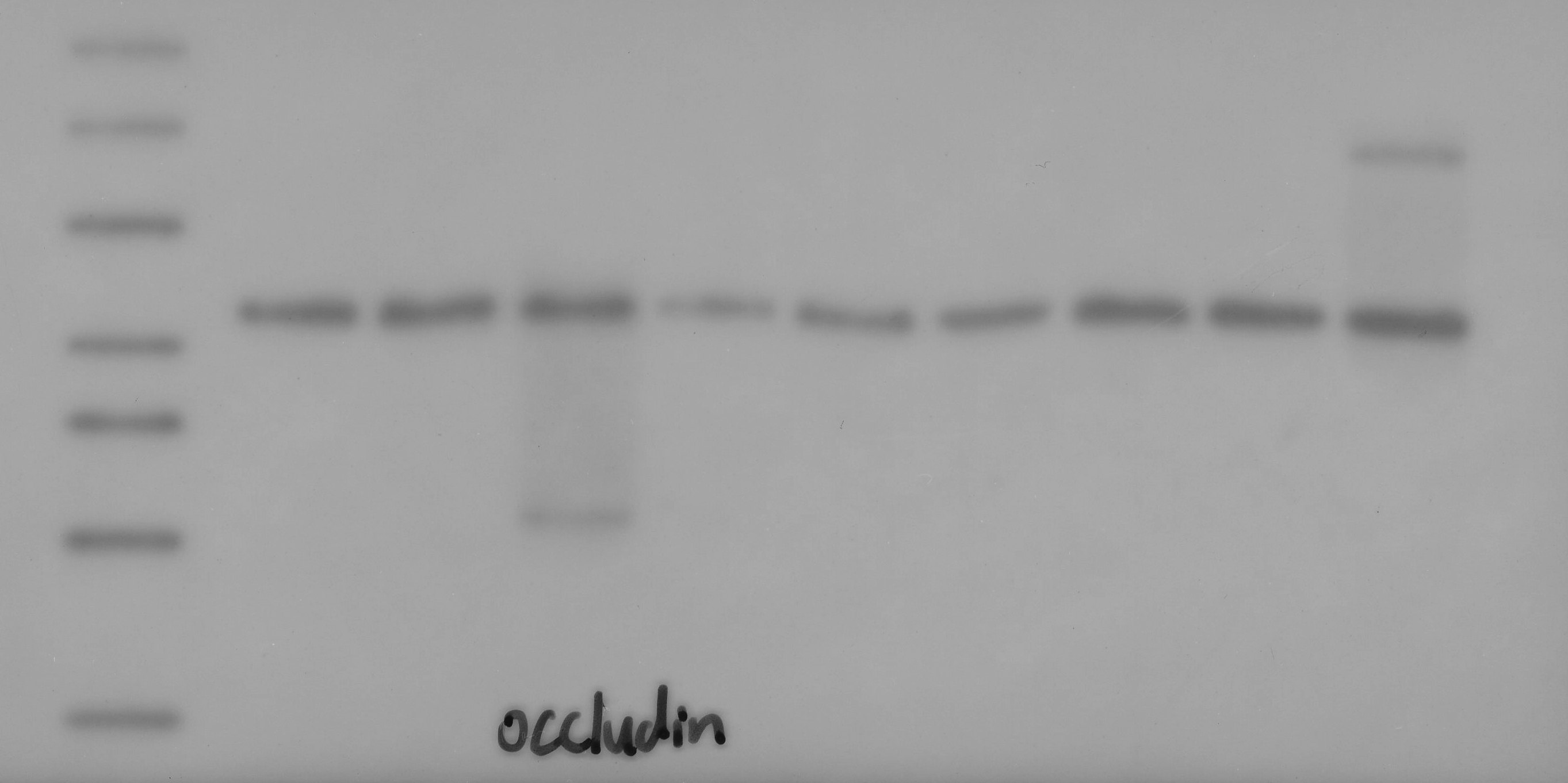

Supplement: Figure 3—source data 2. [file elife-92906-fig3-data2.zip › Figure 3-source data 2/Figure3-Occludin.tif]

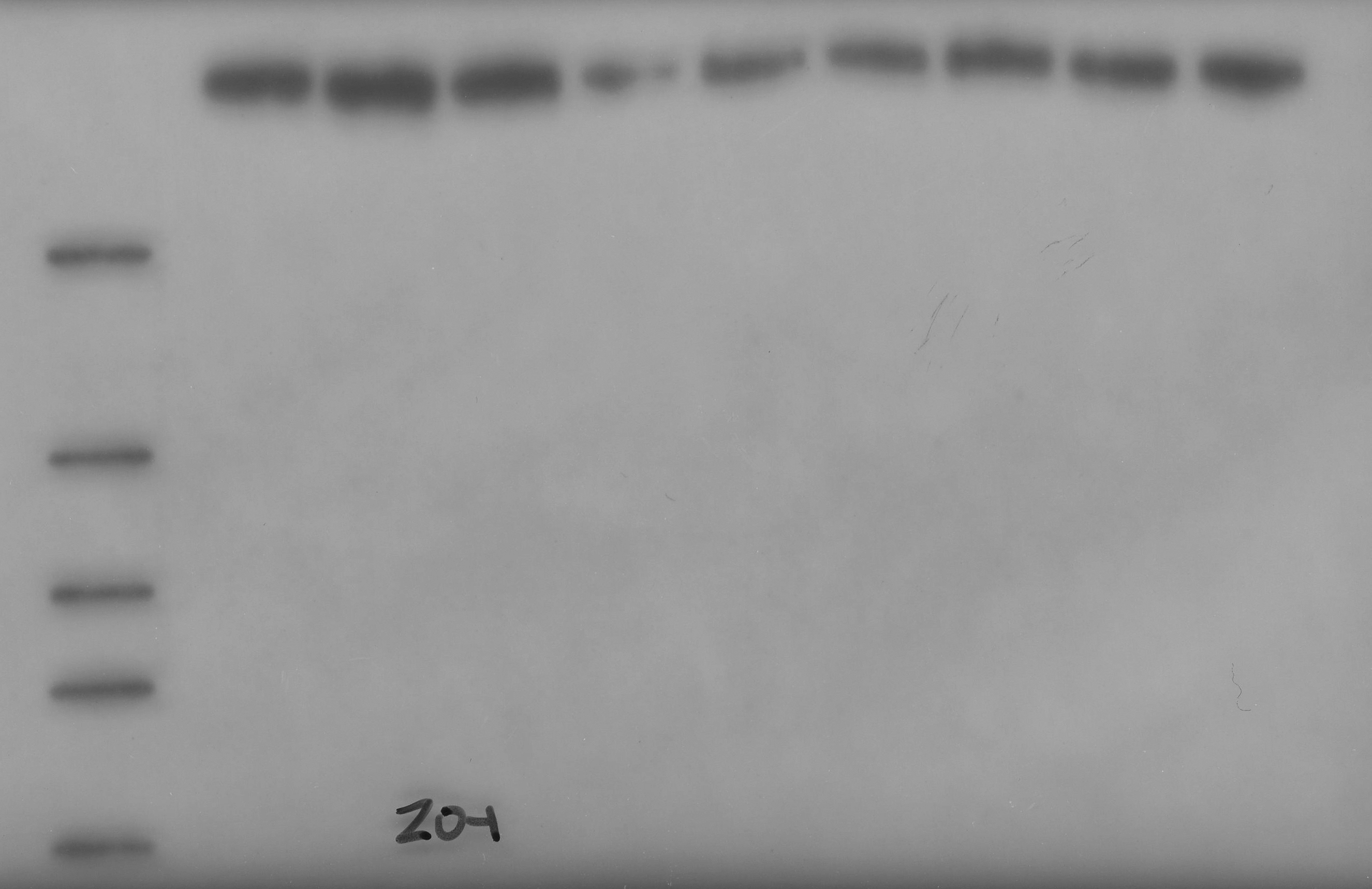

Supplement: Figure 3—source data 2. [file elife-92906-fig3-data2.zip › Figure 3-source data 2/Figure3-ZO-1.tif]

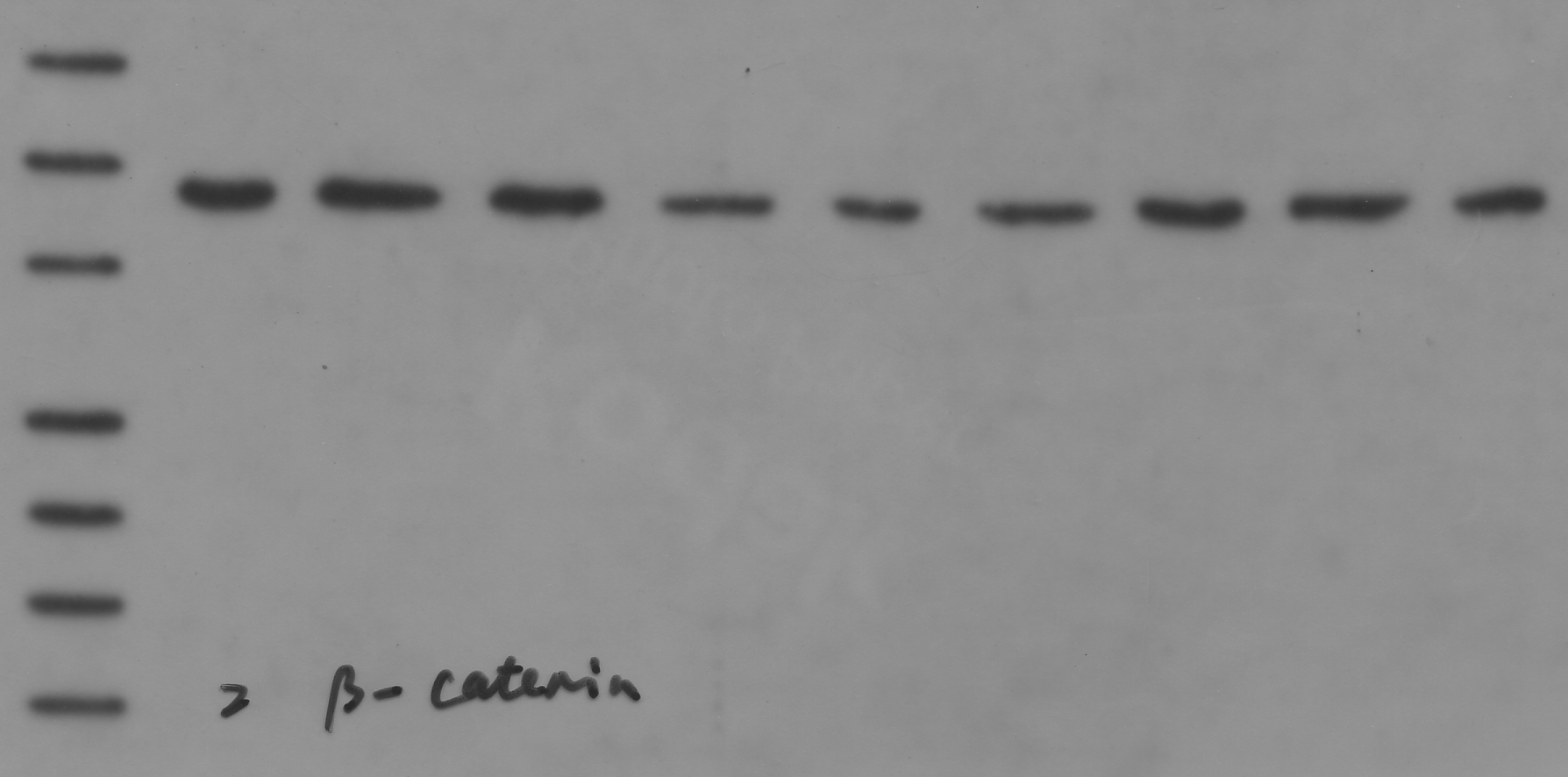

Supplement: Figure 3—source data 2. [file elife-92906-fig3-data2.zip › Figure 3-source data 2/Figure3-β-catenin.tif]

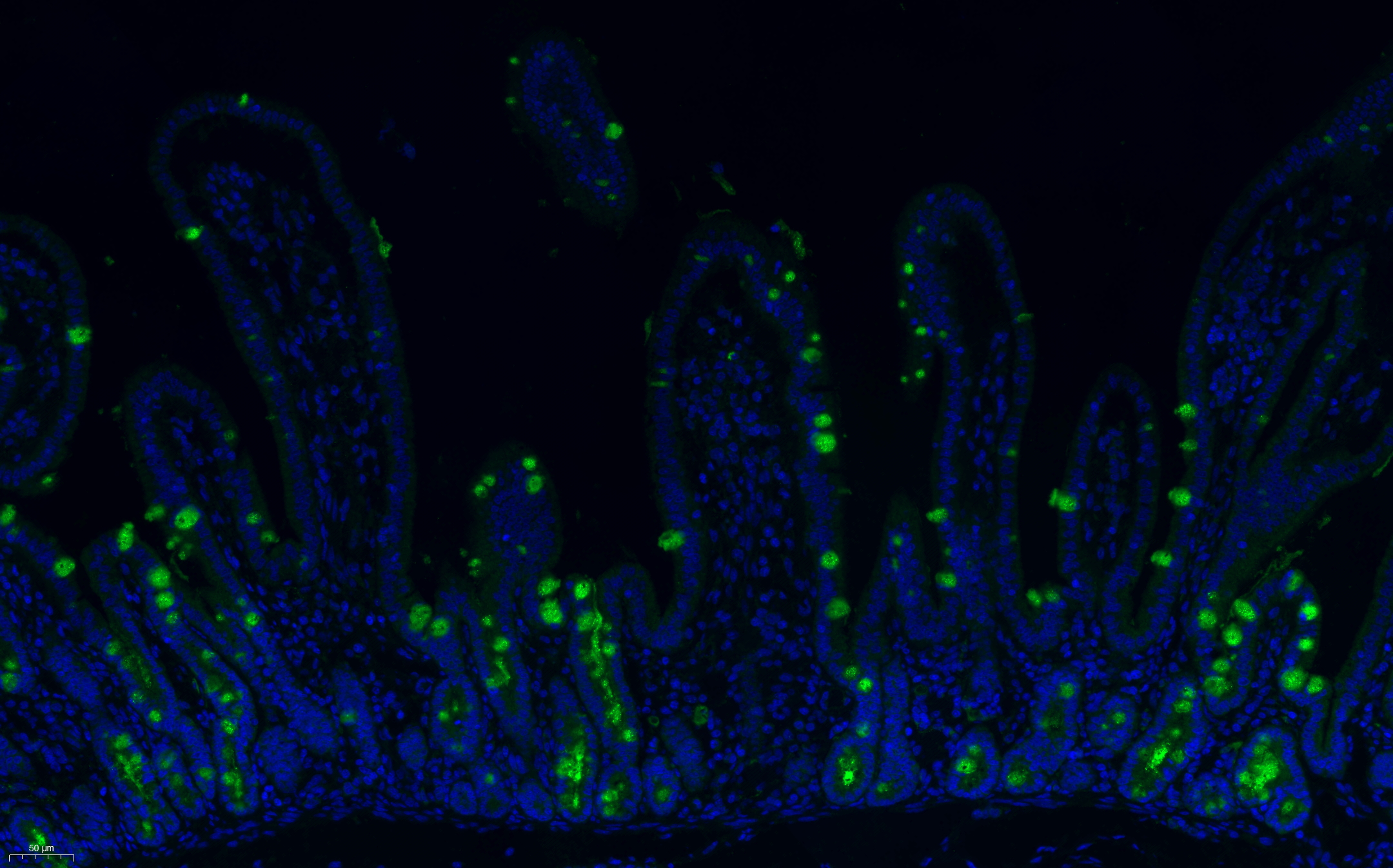

Supplement: Figure 3—source data 3. [file elife-92906-fig3-data3.zip › Figure 3-source data 3/Figure3-MUC2-Con.jpg]

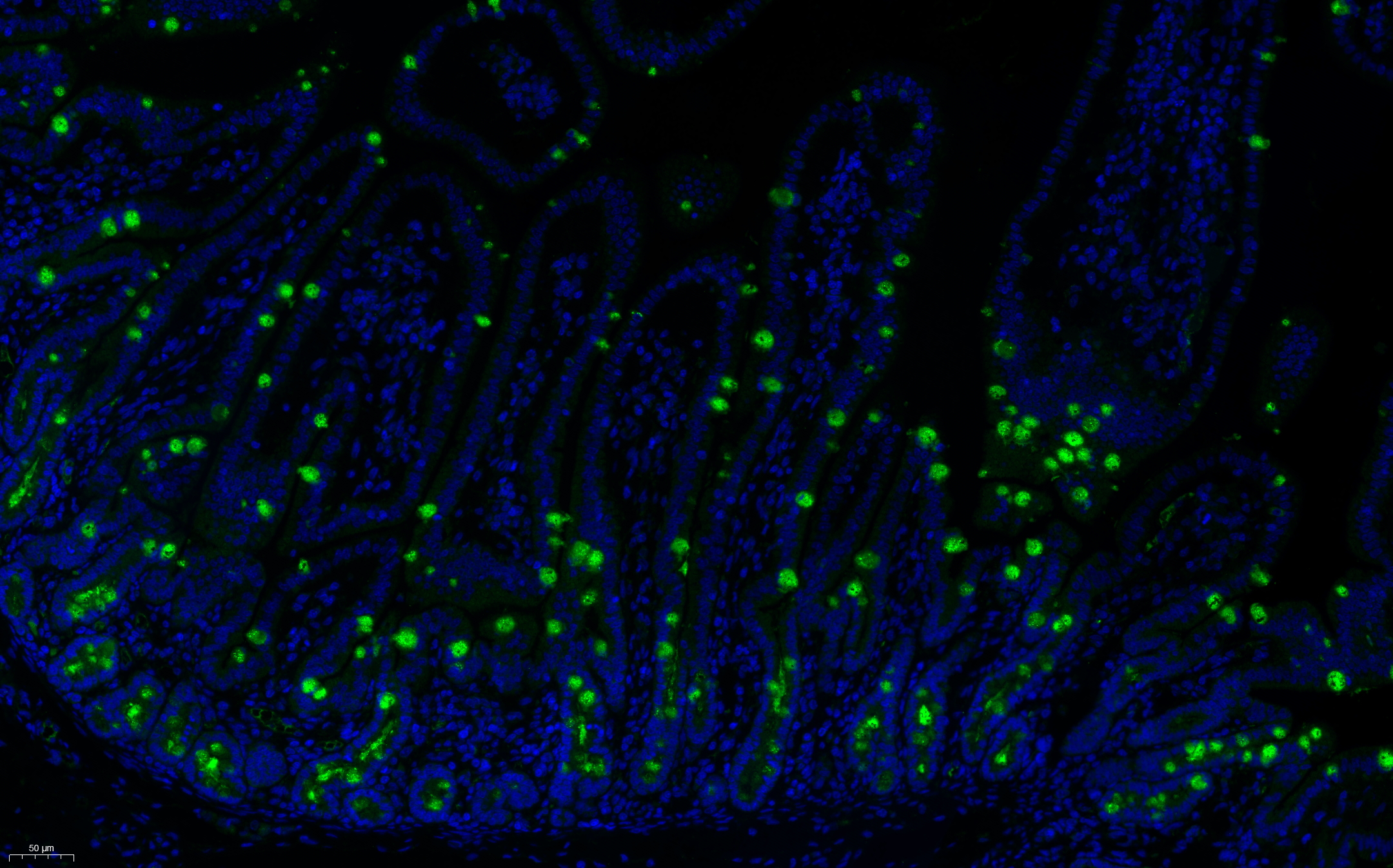

Supplement: Figure 3—source data 3. [file elife-92906-fig3-data3.zip › Figure 3-source data 3/Figure3-MUC2-EF.jpg]

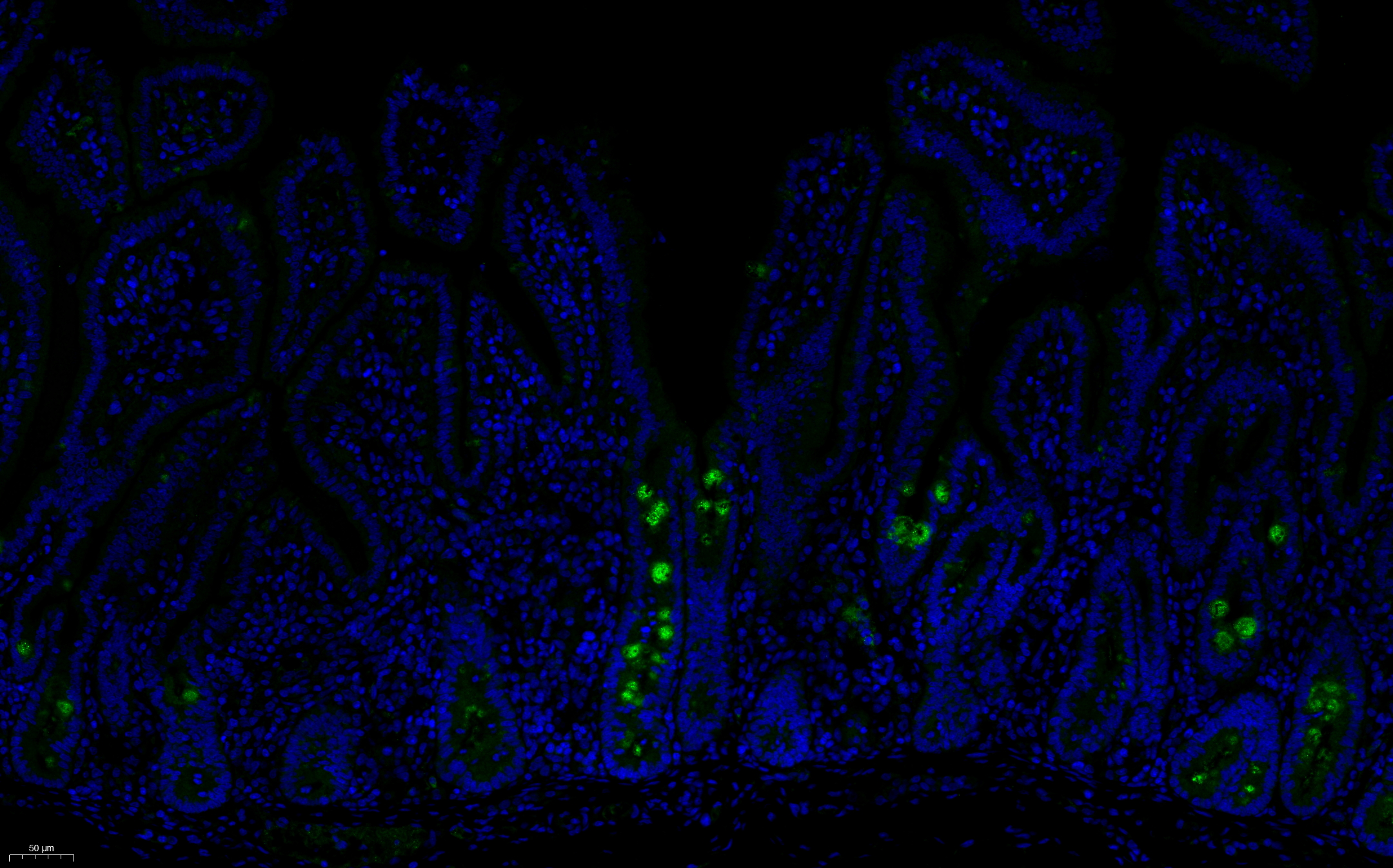

Supplement: Figure 3—source data 3. [file elife-92906-fig3-data3.zip › Figure 3-source data 3/Figure3-MUC2-EP.jpg]

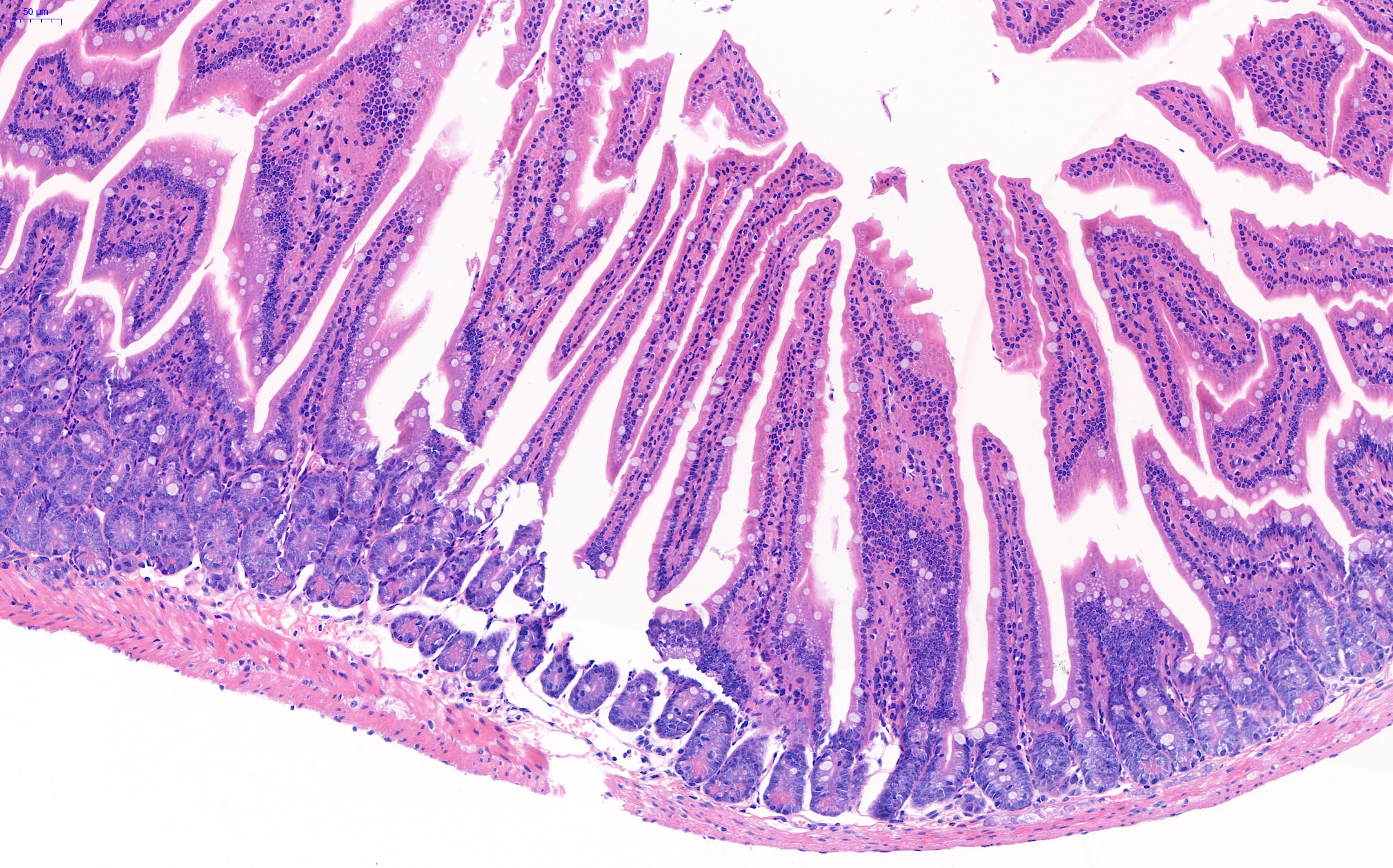

Supplement: Figure 5—source data 1. [file elife-92906-fig5-data1.zip › Figure 5-source data 1/Figure5-HE-A.jpg]

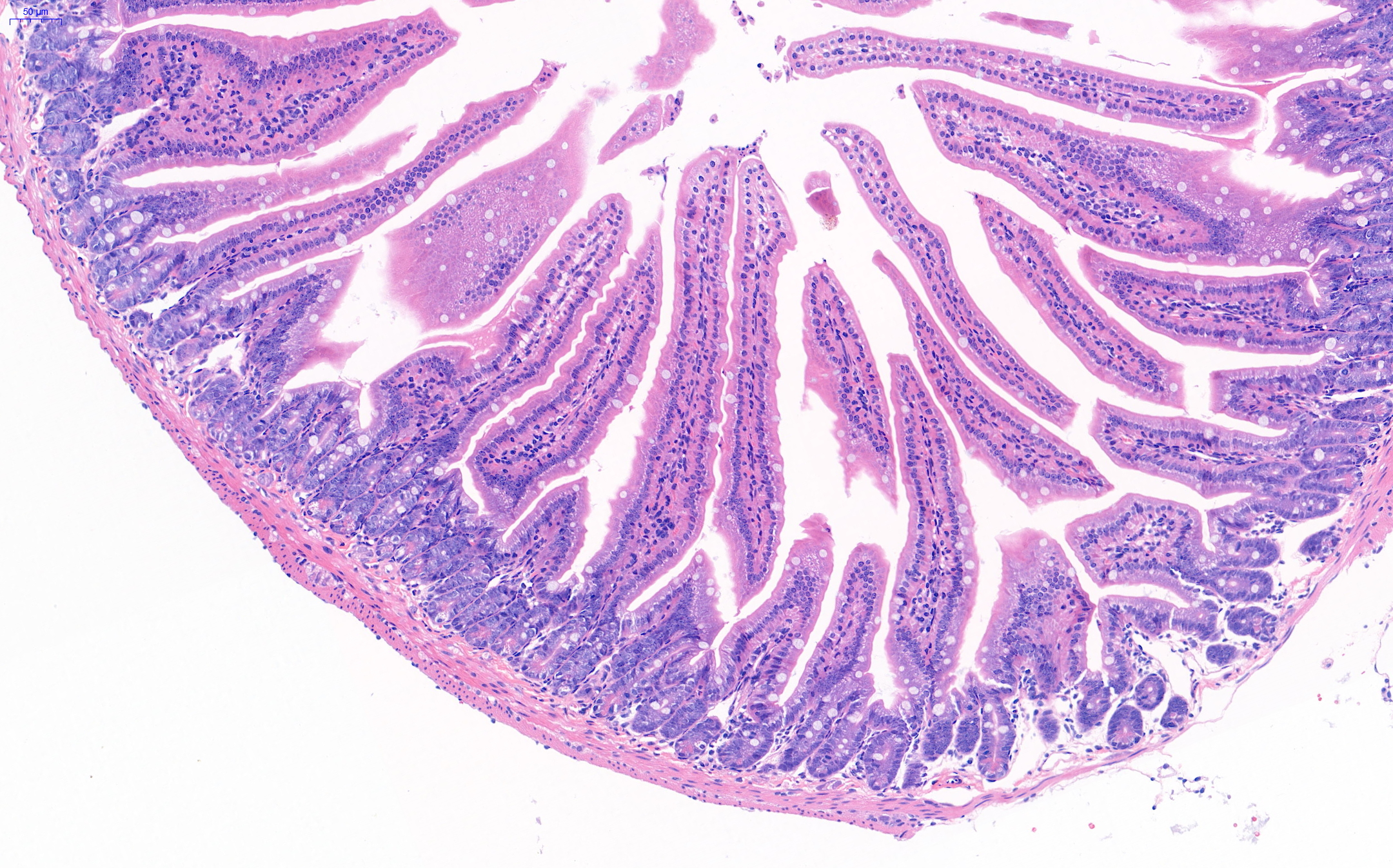

Supplement: Figure 5—source data 1. [file elife-92906-fig5-data1.zip › Figure 5-source data 1/Figure5-HE-B.jpg]

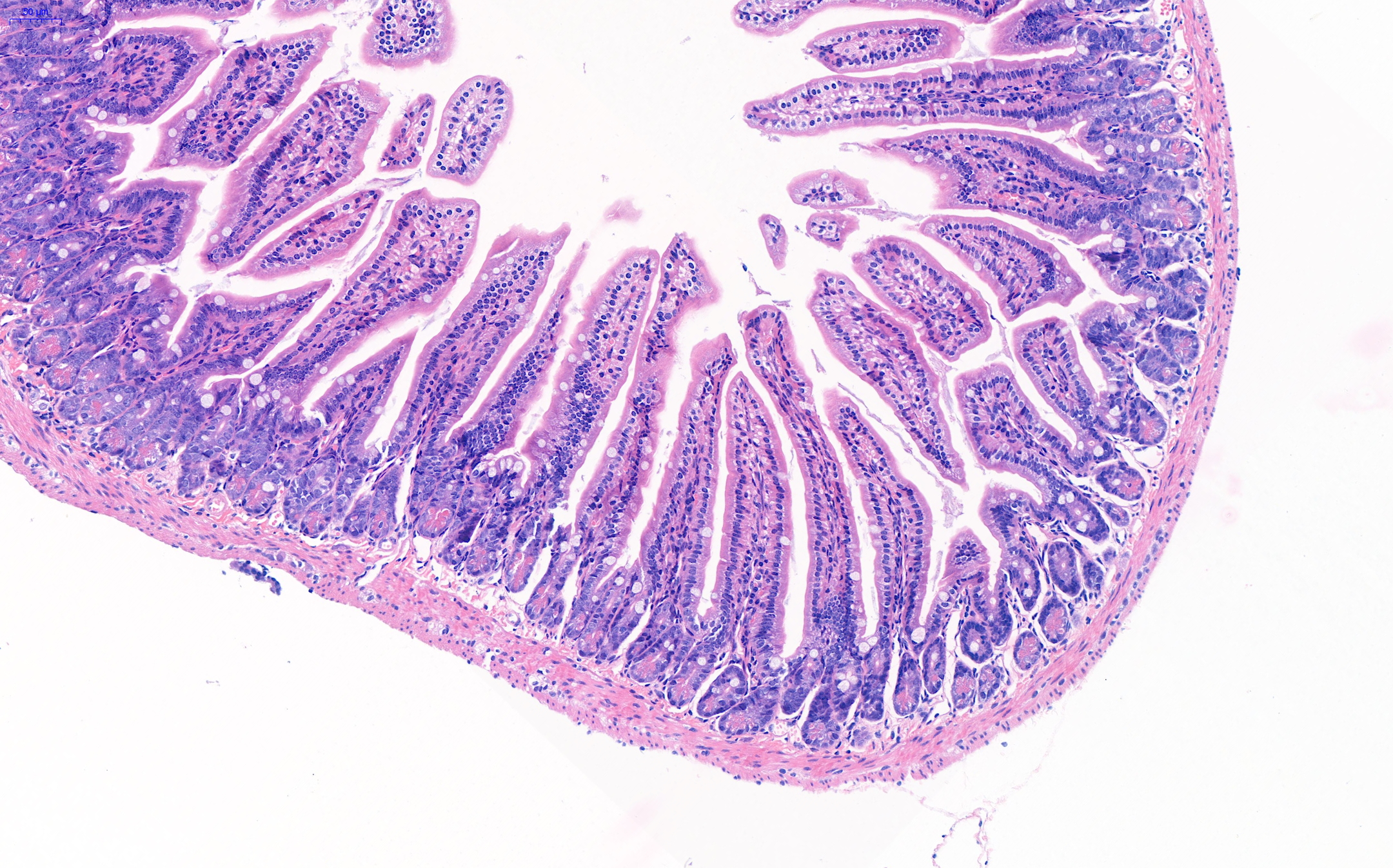

Supplement: Figure 5—source data 1. [file elife-92906-fig5-data1.zip › Figure 5-source data 1/Figure5-HE-C.jpg]

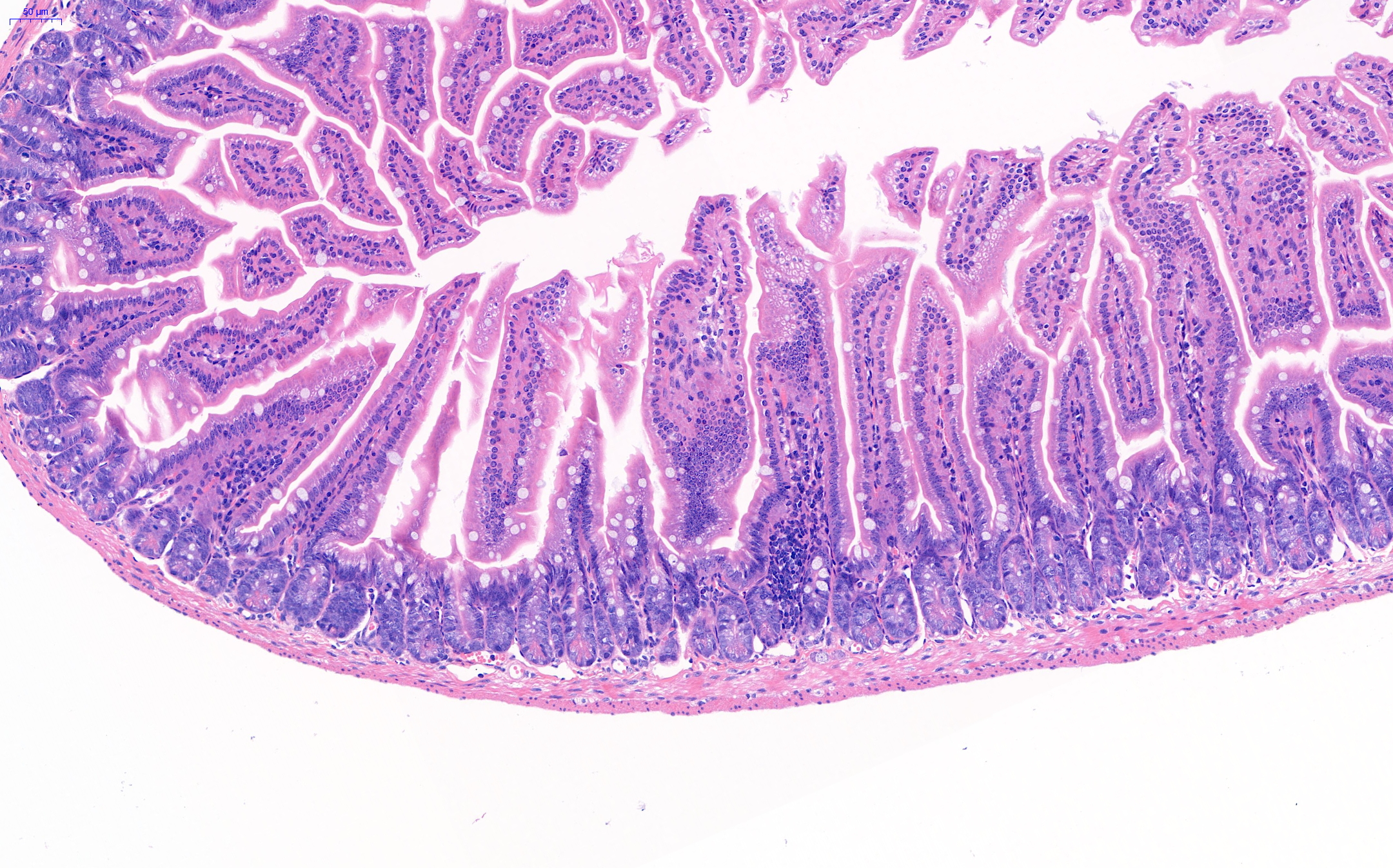

Supplement: Figure 5—source data 1. [file elife-92906-fig5-data1.zip › Figure 5-source data 1/Figure5-HE-E.jpg]

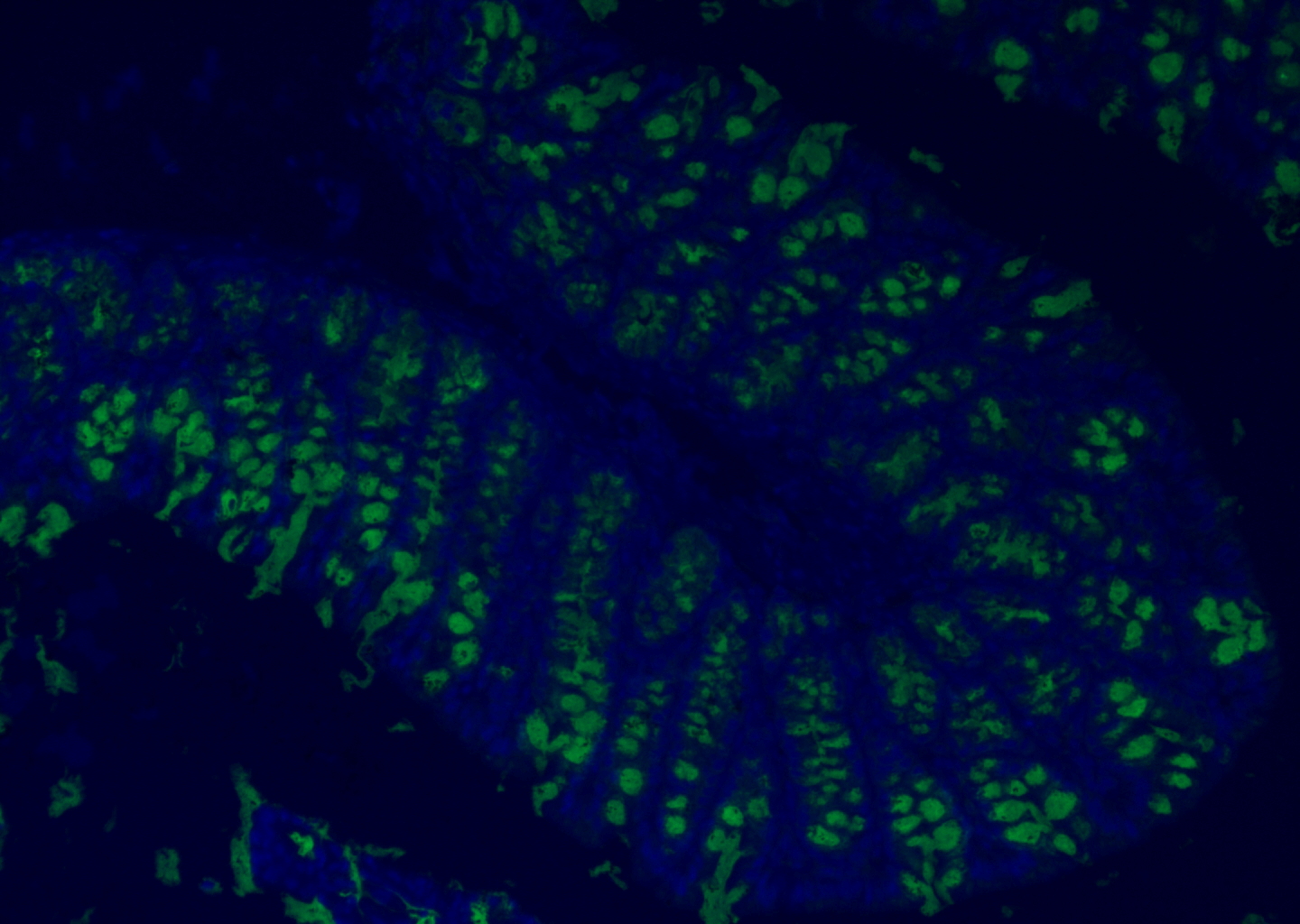

Supplement: Figure 5—source data 3. [file elife-92906-fig5-data3.zip › Figure 5-source data 3/Figure5-CO-MUC2-A.jpg]

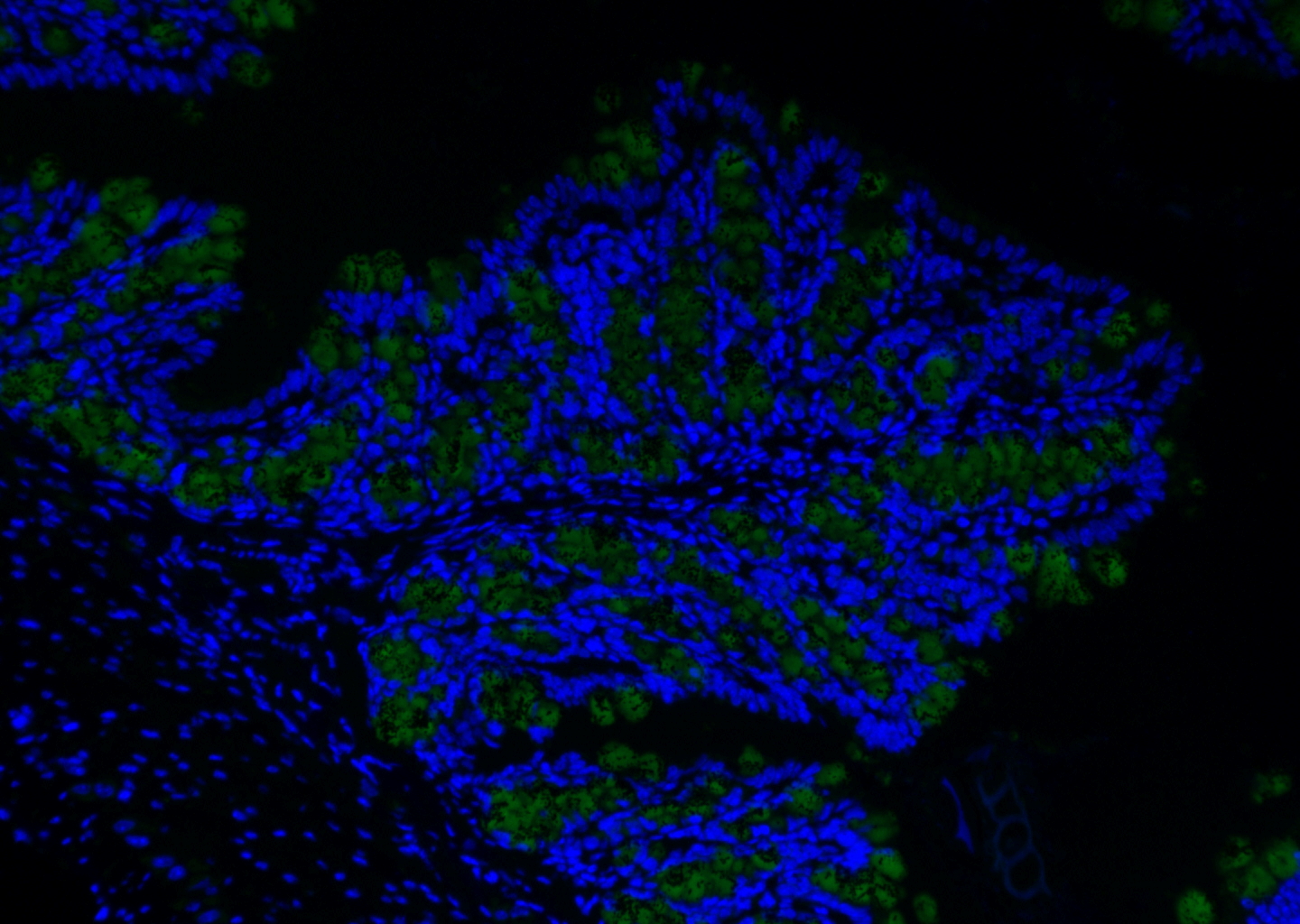

Supplement: Figure 5—source data 3. [file elife-92906-fig5-data3.zip › Figure 5-source data 3/Figure5-CO-MUC2-B.jpg]

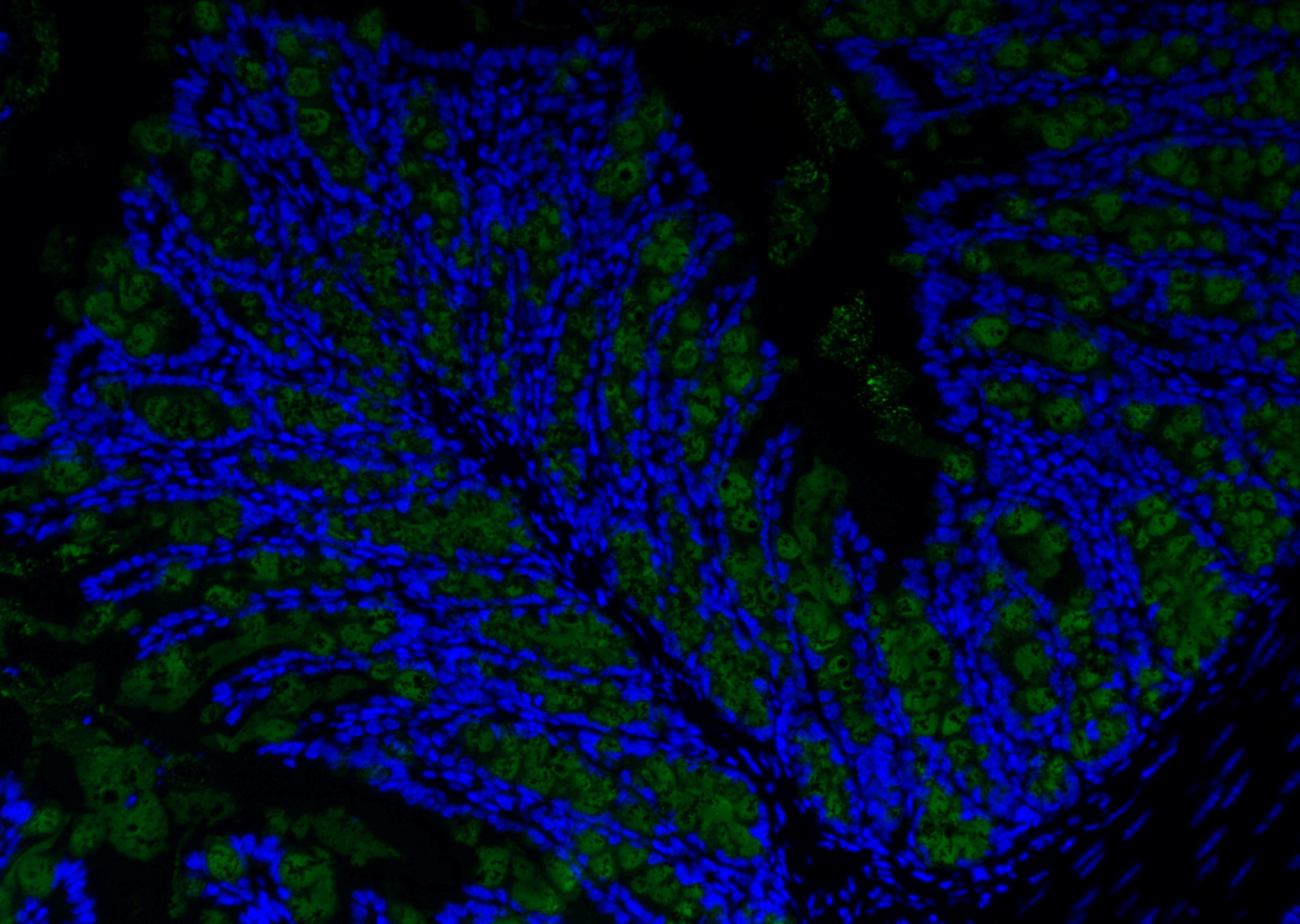

Supplement: Figure 5—source data 3. [file elife-92906-fig5-data3.zip › Figure 5-source data 3/Figure5-CO-MUC2-C.jpg]

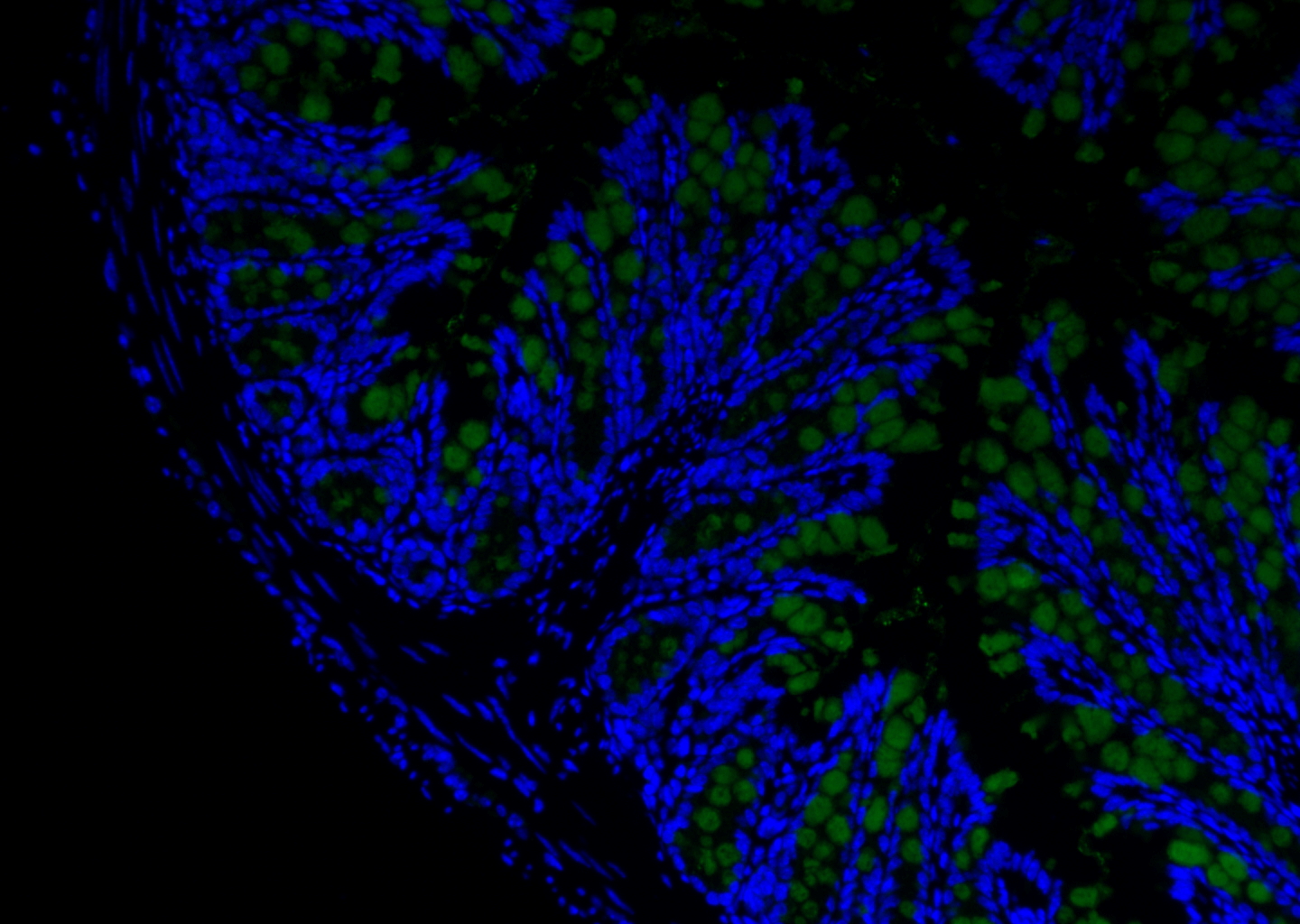

Supplement: Figure 5—source data 3. [file elife-92906-fig5-data3.zip › Figure 5-source data 3/Figure5-CO-MUC2-E.jpg]

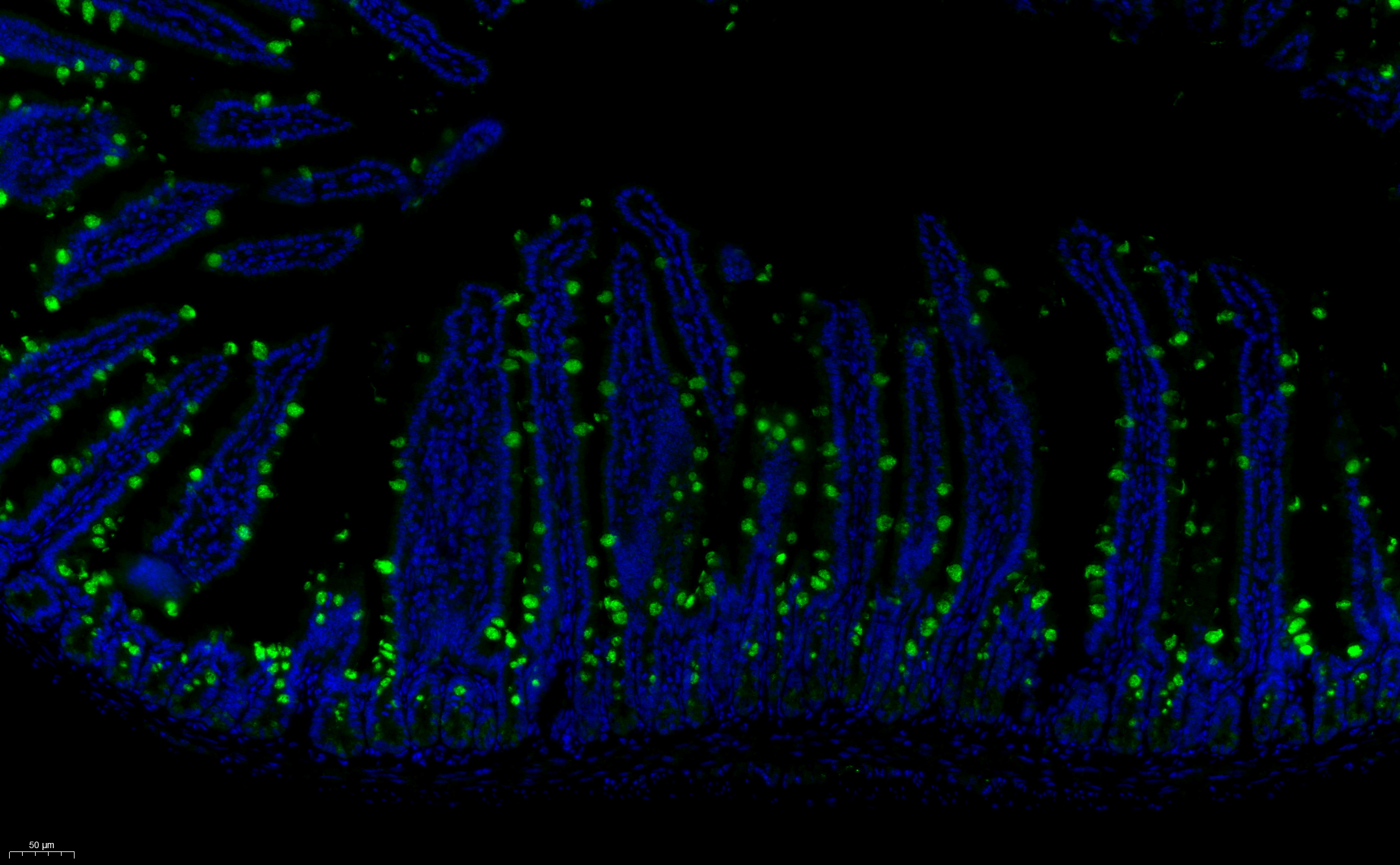

Supplement: Figure 5—source data 3. [file elife-92906-fig5-data3.zip › Figure 5-source data 3/Figure5-JE-MUC2-A.jpg]

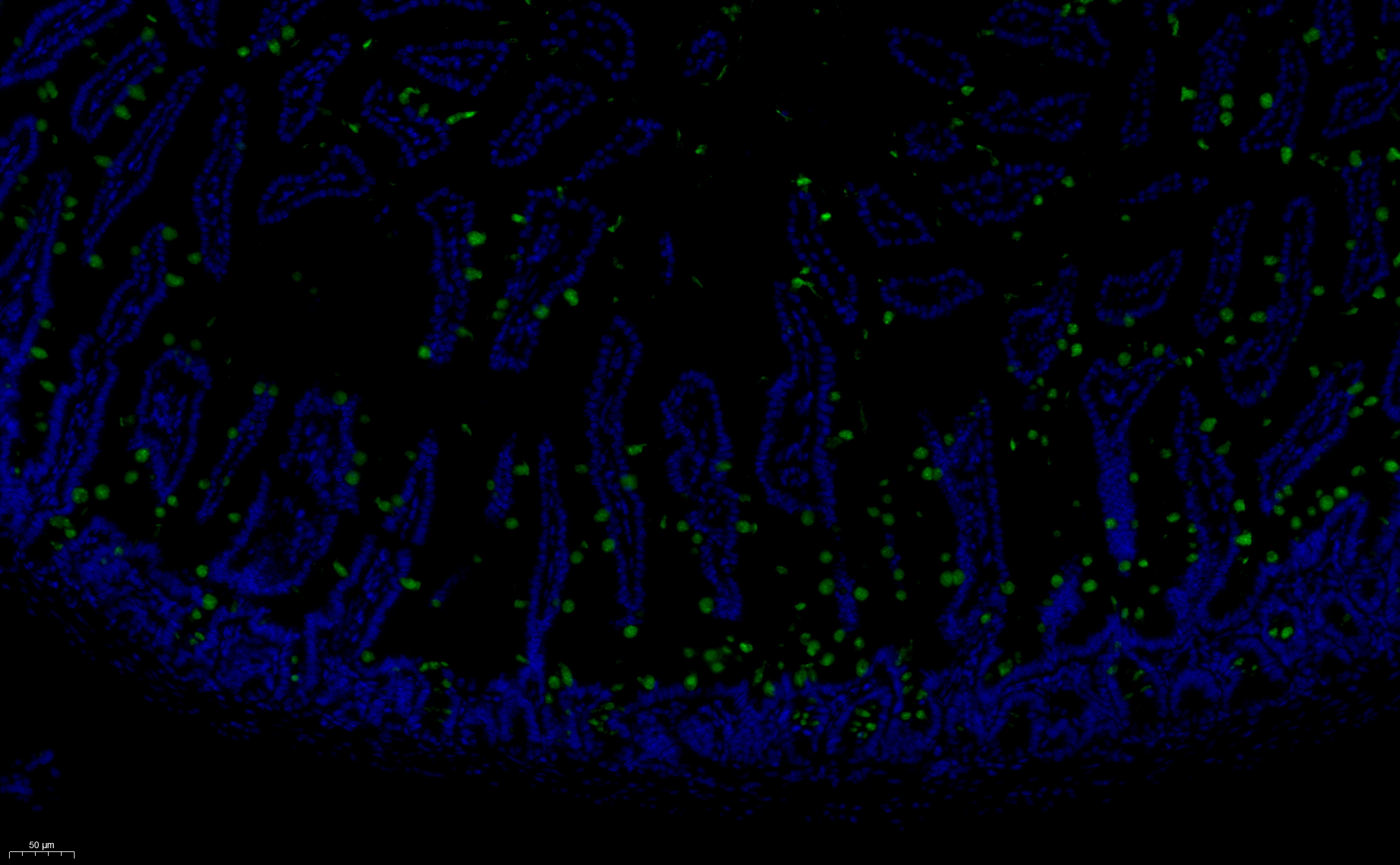

Supplement: Figure 5—source data 3. [file elife-92906-fig5-data3.zip › Figure 5-source data 3/Figure5-JE-MUC2-B.jpg]

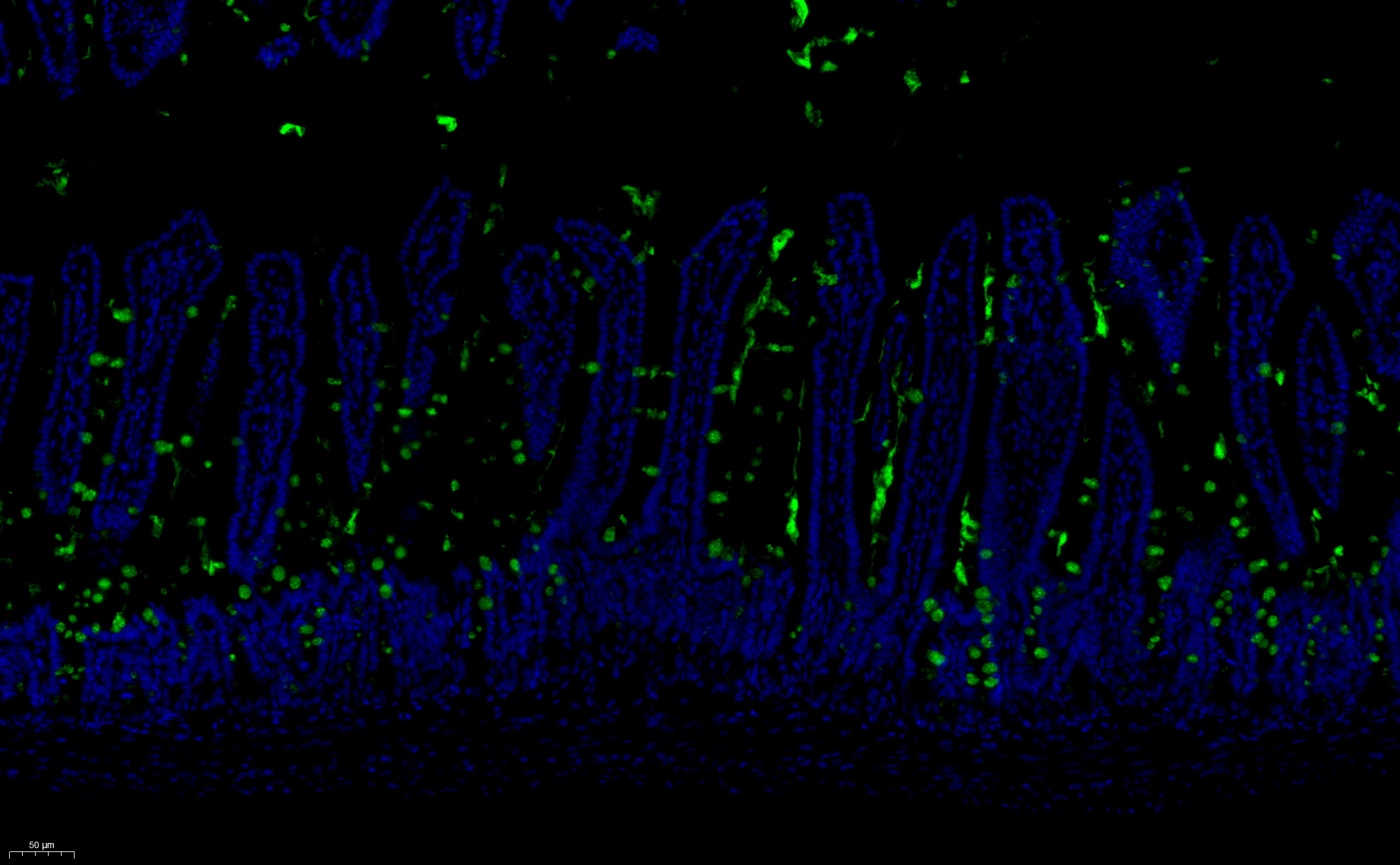

Supplement: Figure 5—source data 3. [file elife-92906-fig5-data3.zip › Figure 5-source data 3/Figure5-JE-MUC2-C.jpg]

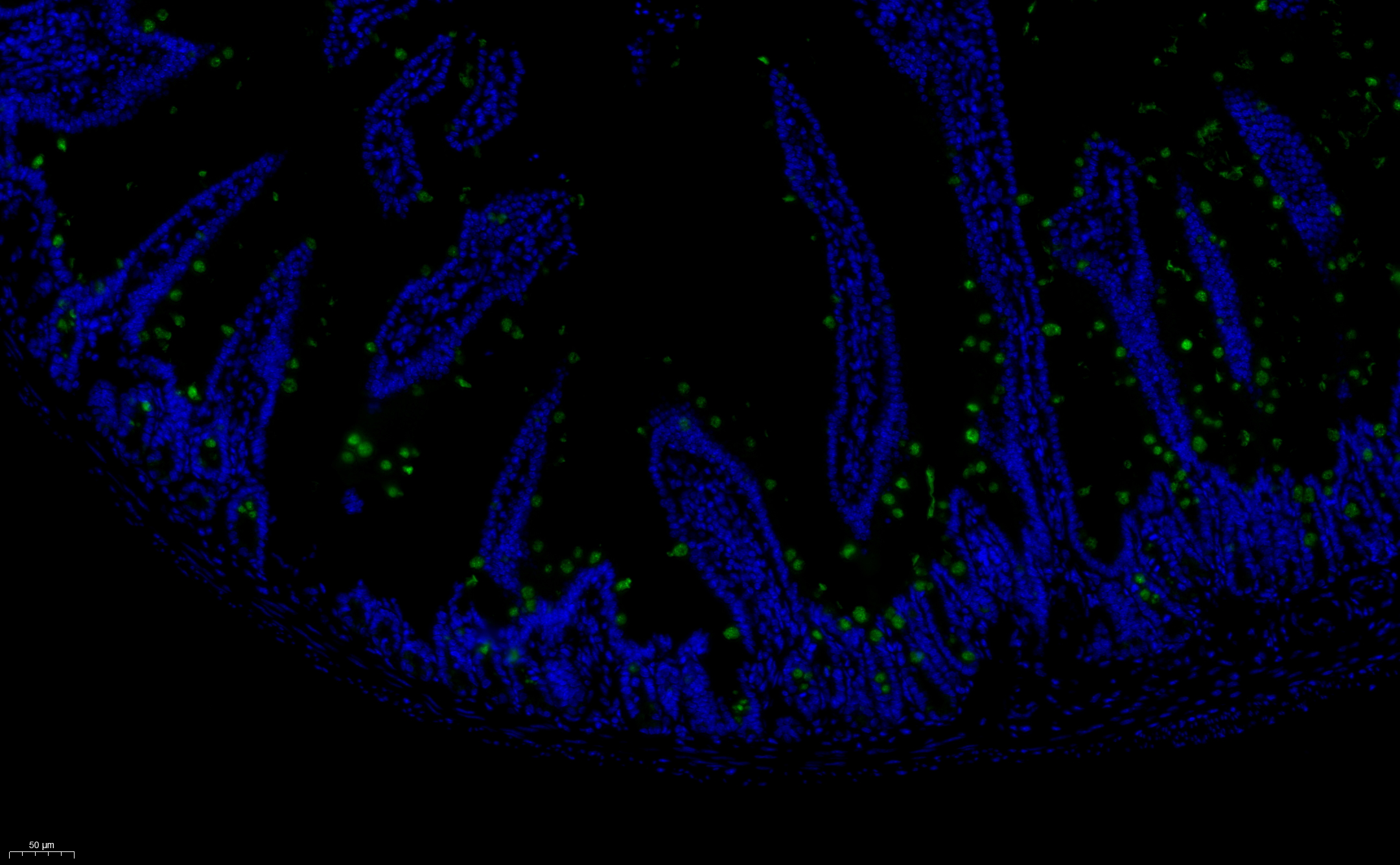

Supplement: Figure 5—source data 3. [file elife-92906-fig5-data3.zip › Figure 5-source data 3/Figure5-JE-MUC2-E.jpg]

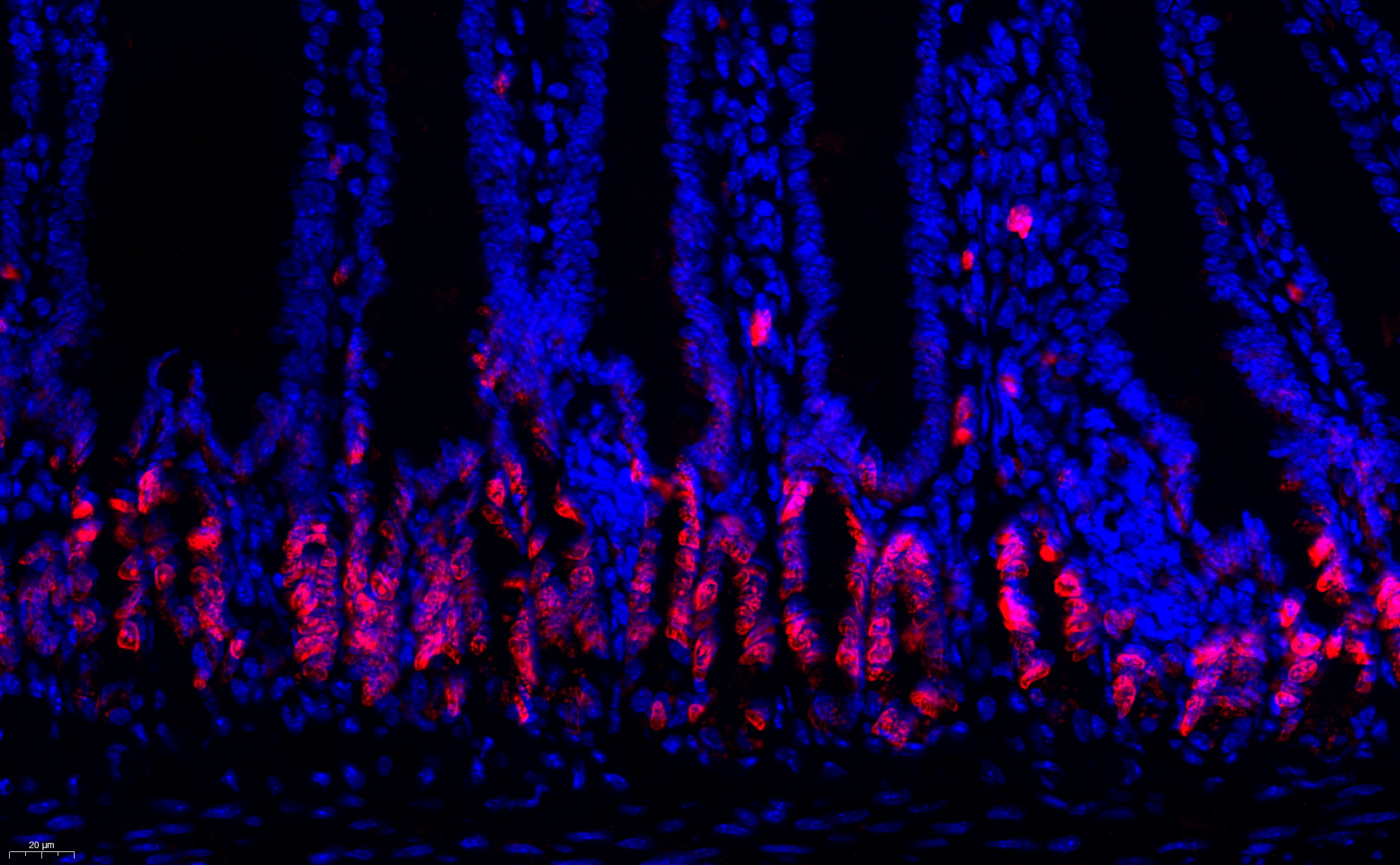

Supplement: Figure 7—source data 1. [file elife-92906-fig7-data1.zip › Figure 7-source data 1/Figure7-KI67-A.jpg]

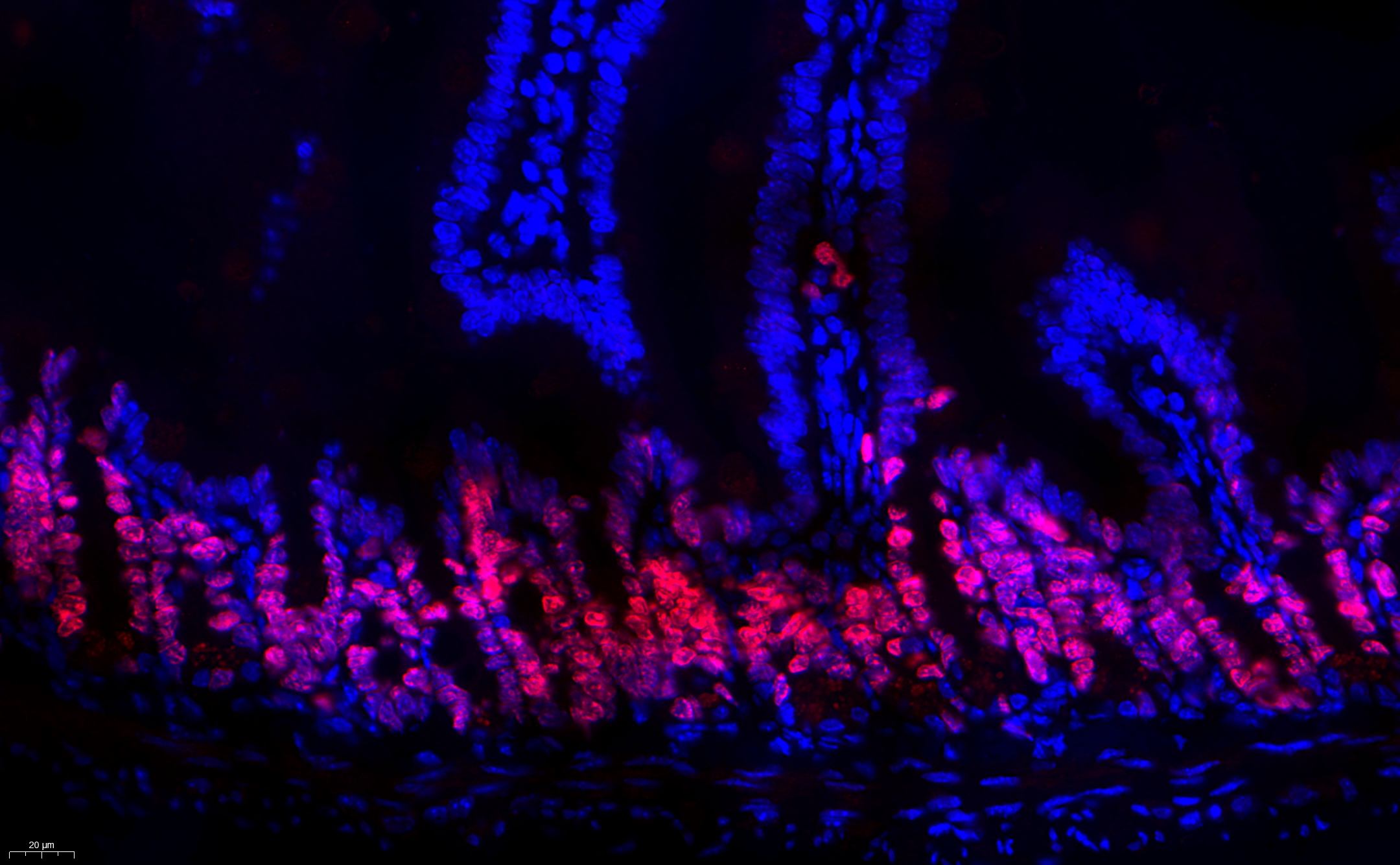

Supplement: Figure 7—source data 1. [file elife-92906-fig7-data1.zip › Figure 7-source data 1/Figure7-KI67-B.jpg]

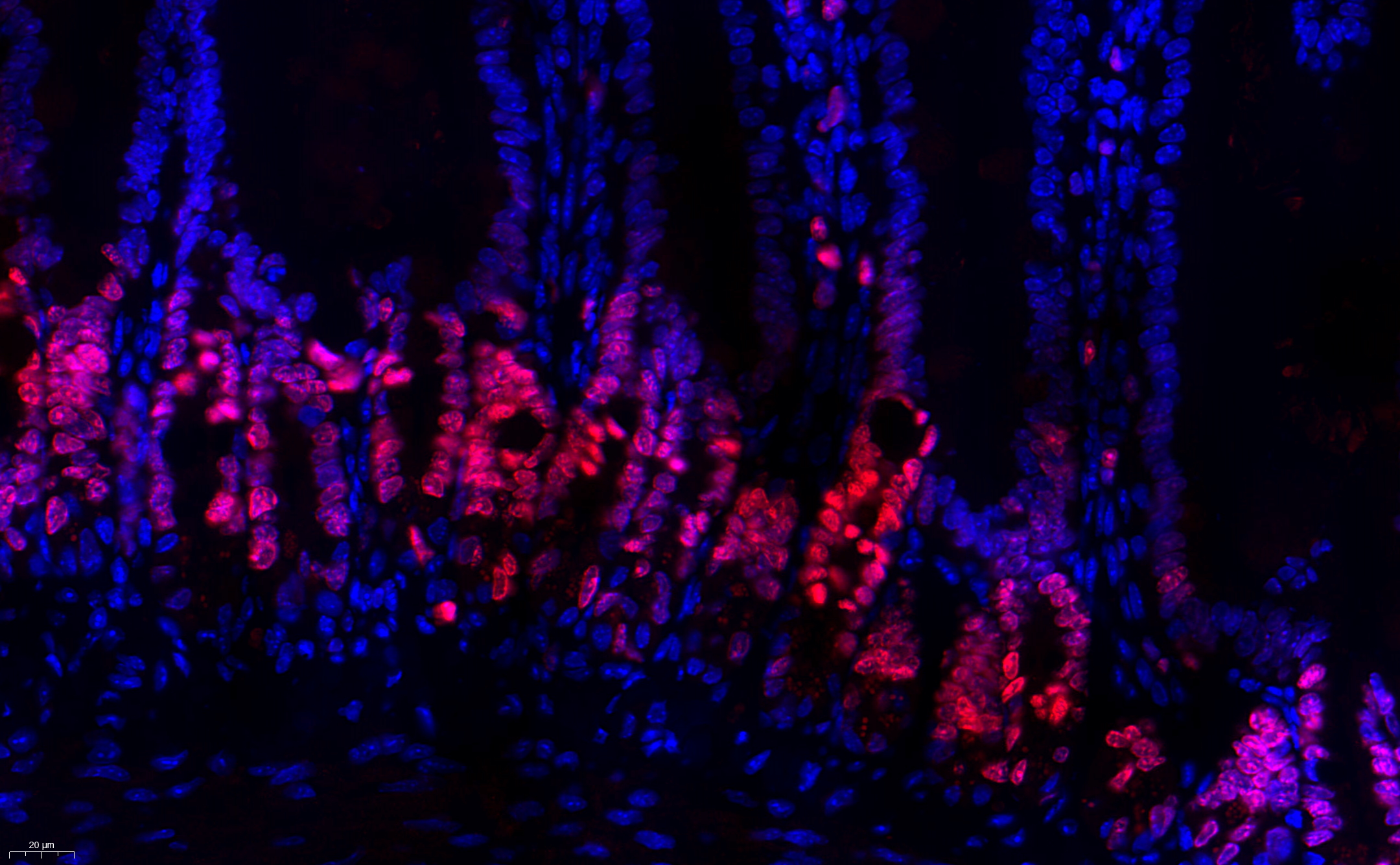

Supplement: Figure 7—source data 1. [file elife-92906-fig7-data1.zip › Figure 7-source data 1/Figure7-KI67-C.jpg]

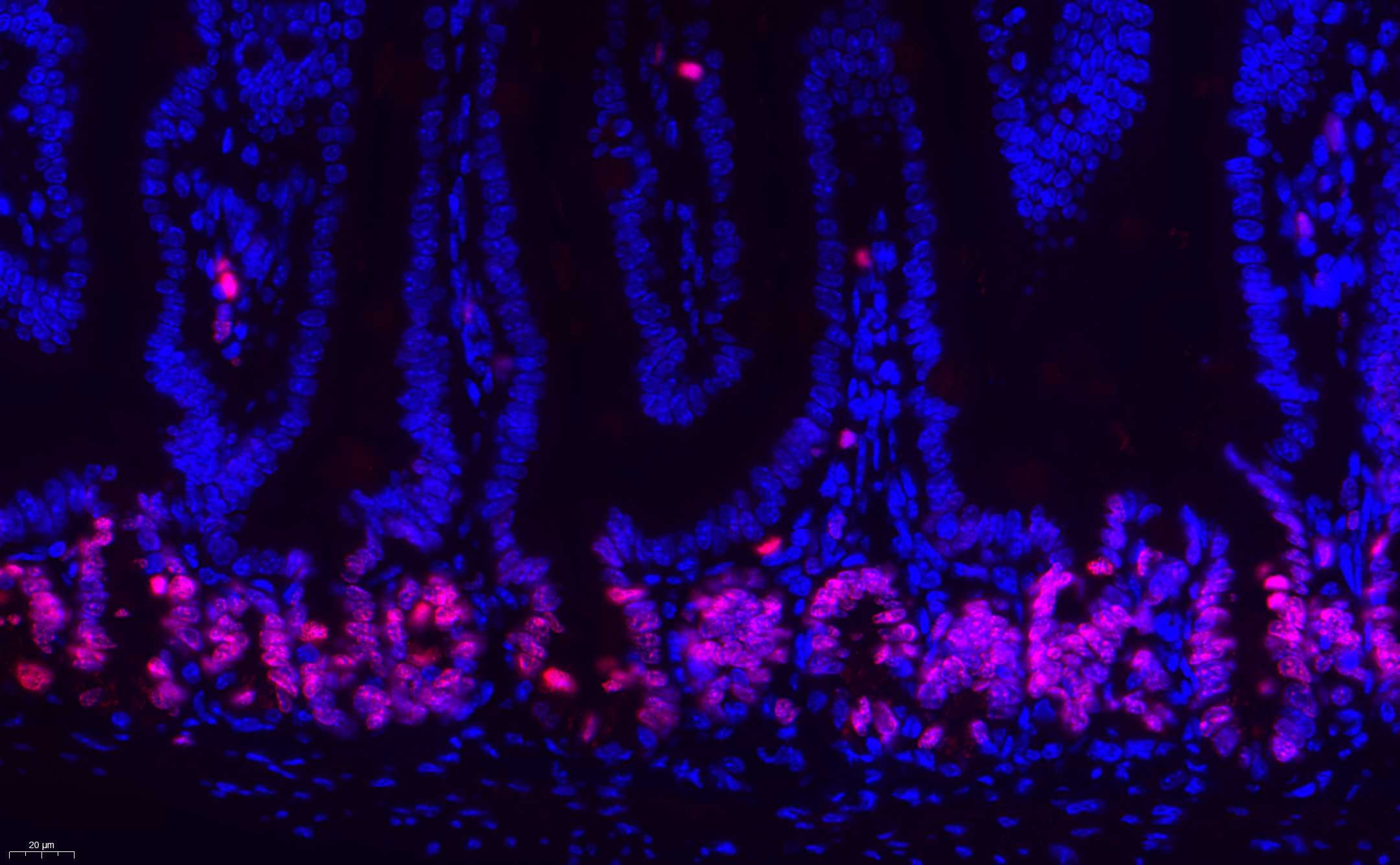

Supplement: Figure 7—source data 1. [file elife-92906-fig7-data1.zip › Figure 7-source data 1/Figure7-KI67-E.jpg]

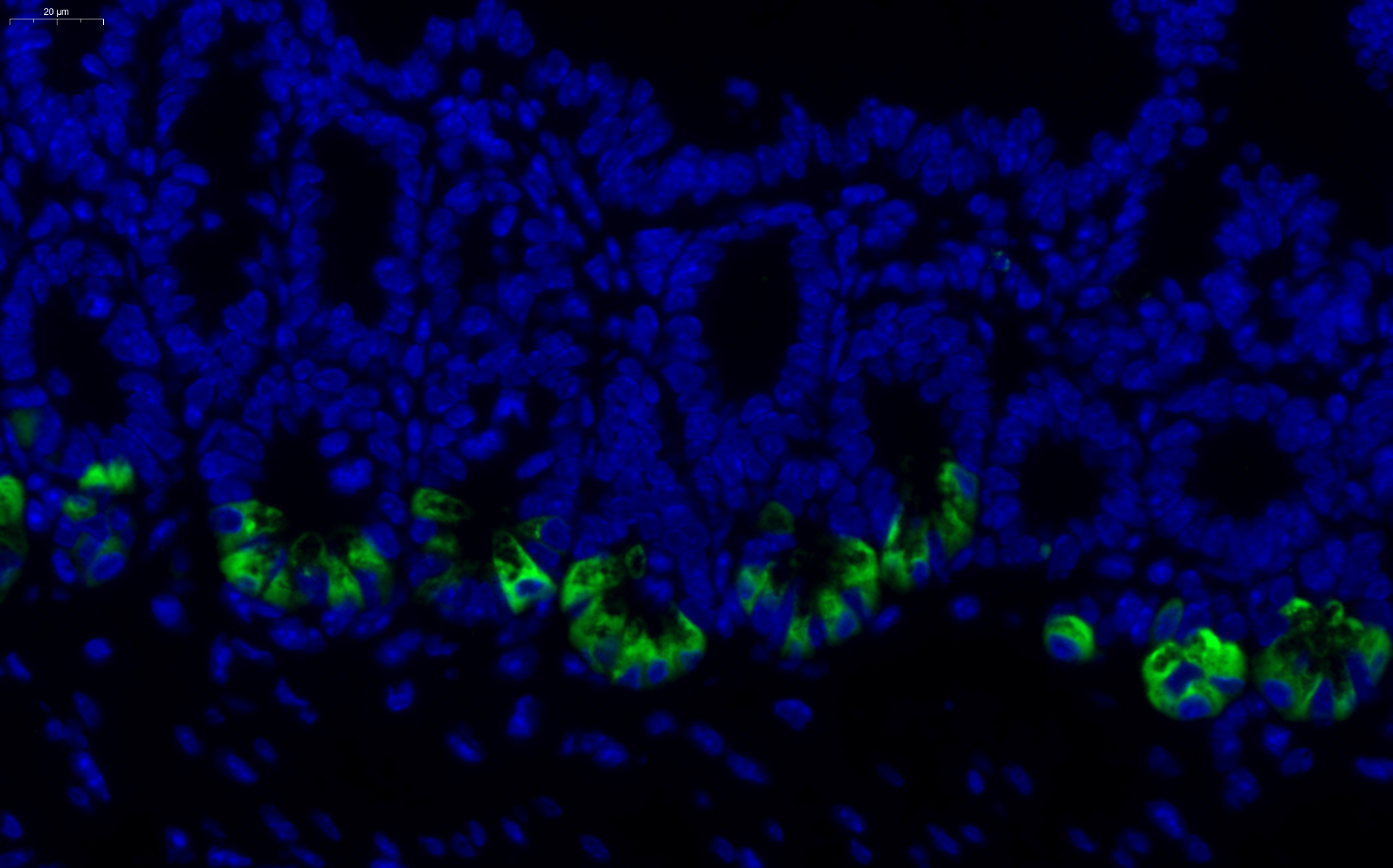

Supplement: Figure 7—source data 1. [file elife-92906-fig7-data1.zip › Figure 7-source data 1/Figure7-LYZ-A.jpg]

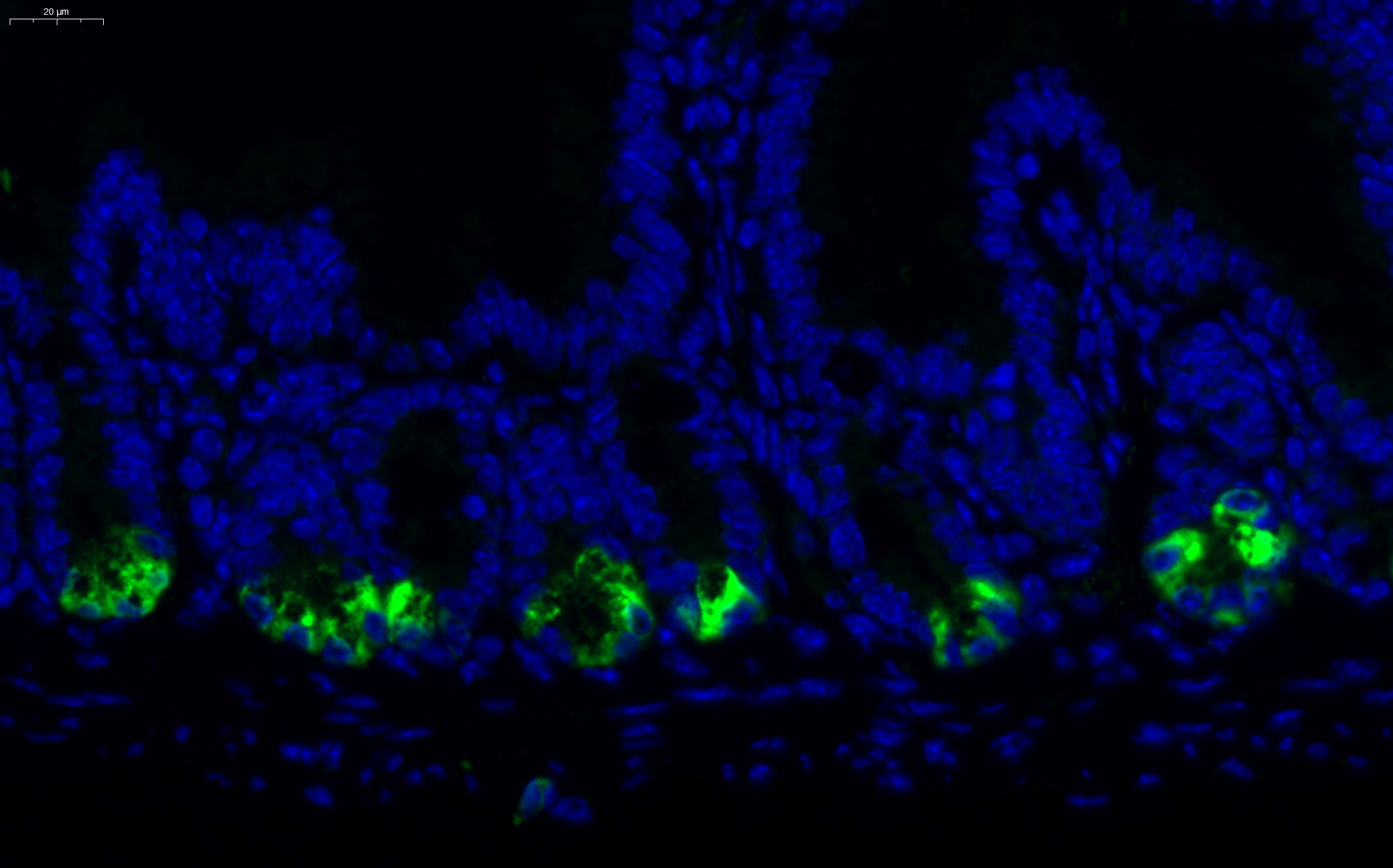

Supplement: Figure 7—source data 1. [file elife-92906-fig7-data1.zip › Figure 7-source data 1/Figure7-LYZ-B.jpg]

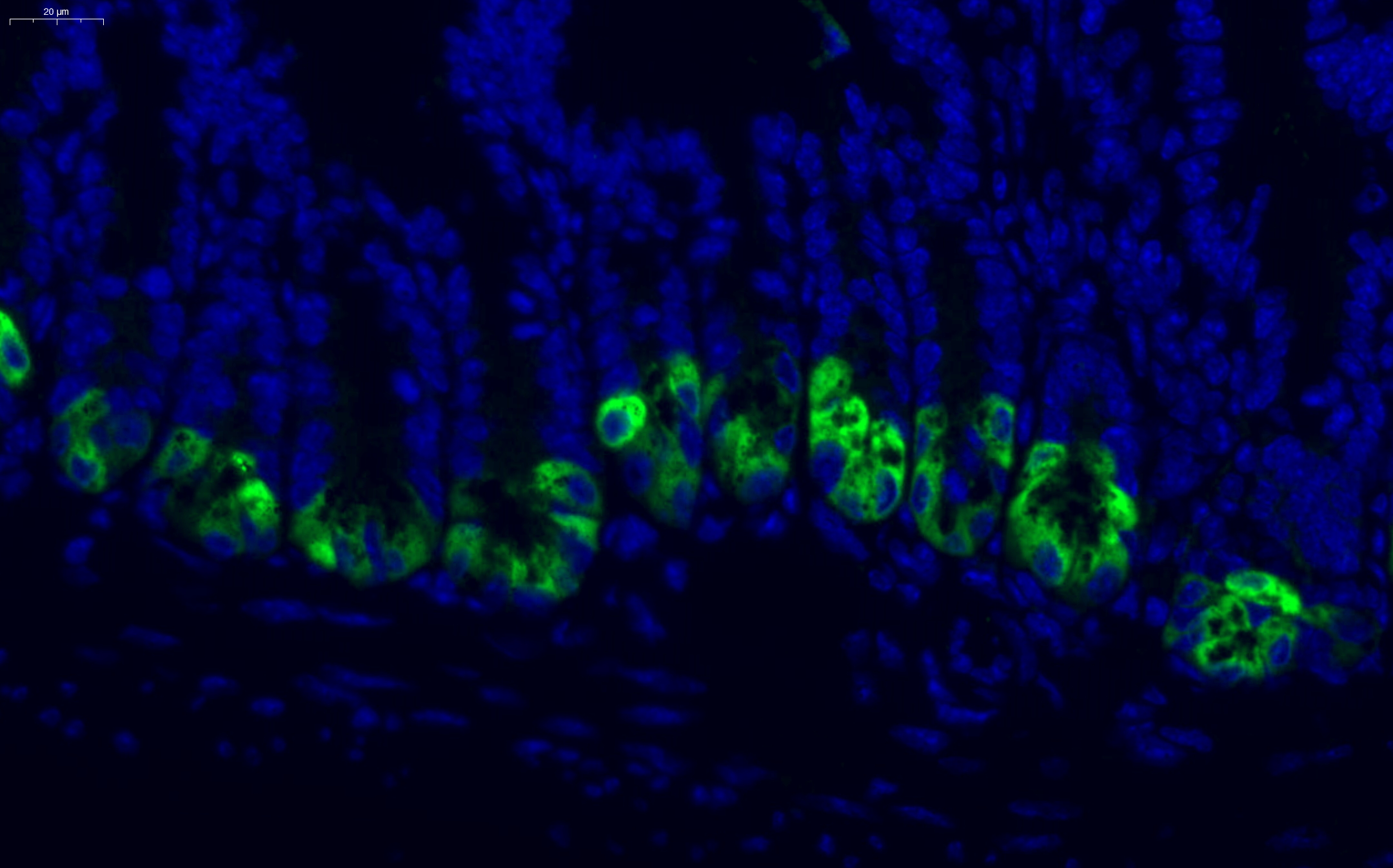

Supplement: Figure 7—source data 1. [file elife-92906-fig7-data1.zip › Figure 7-source data 1/Figure7-LYZ-C.jpg]

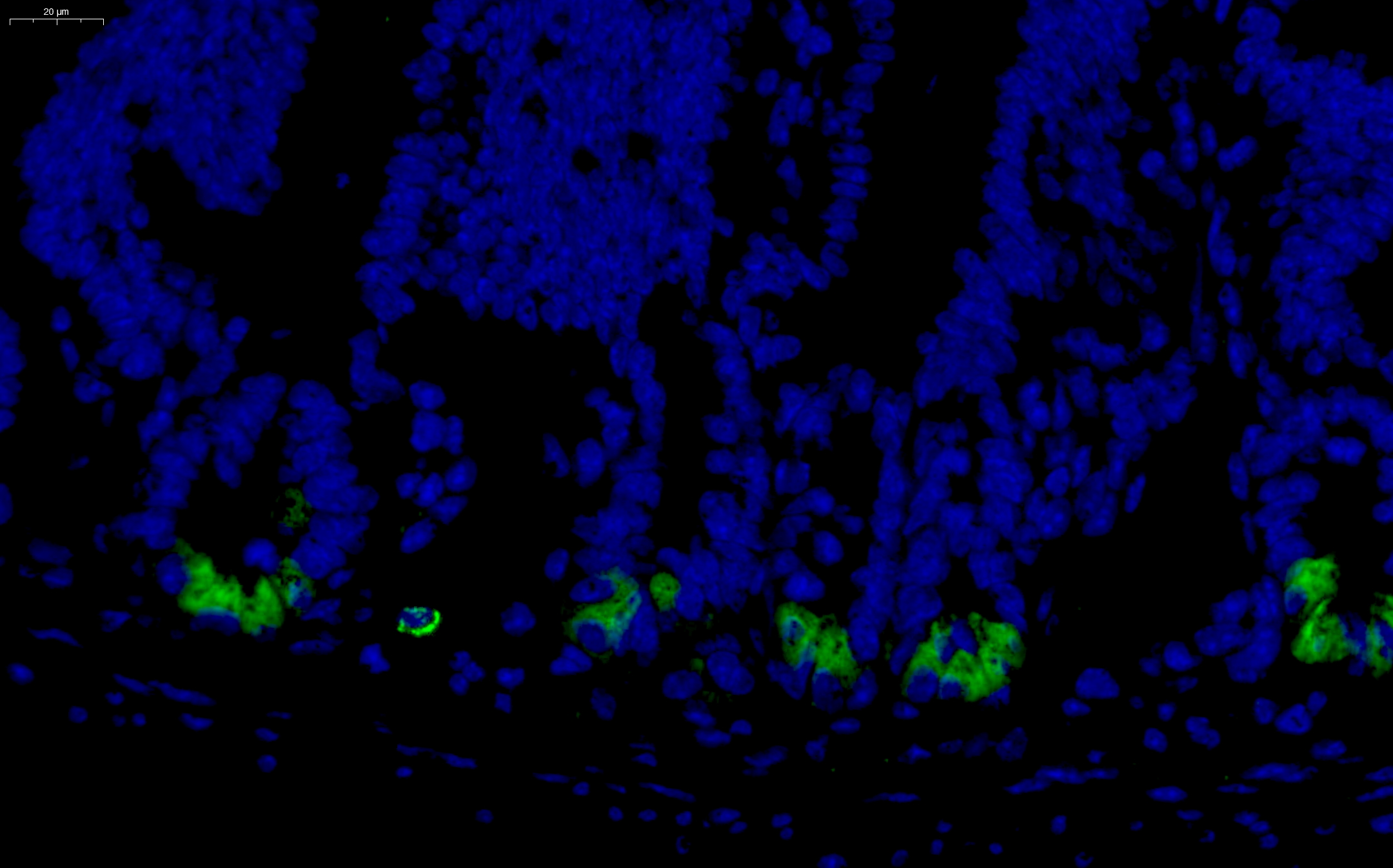

Supplement: Figure 7—source data 1. [file elife-92906-fig7-data1.zip › Figure 7-source data 1/Figure7-LYZ-E.jpg]

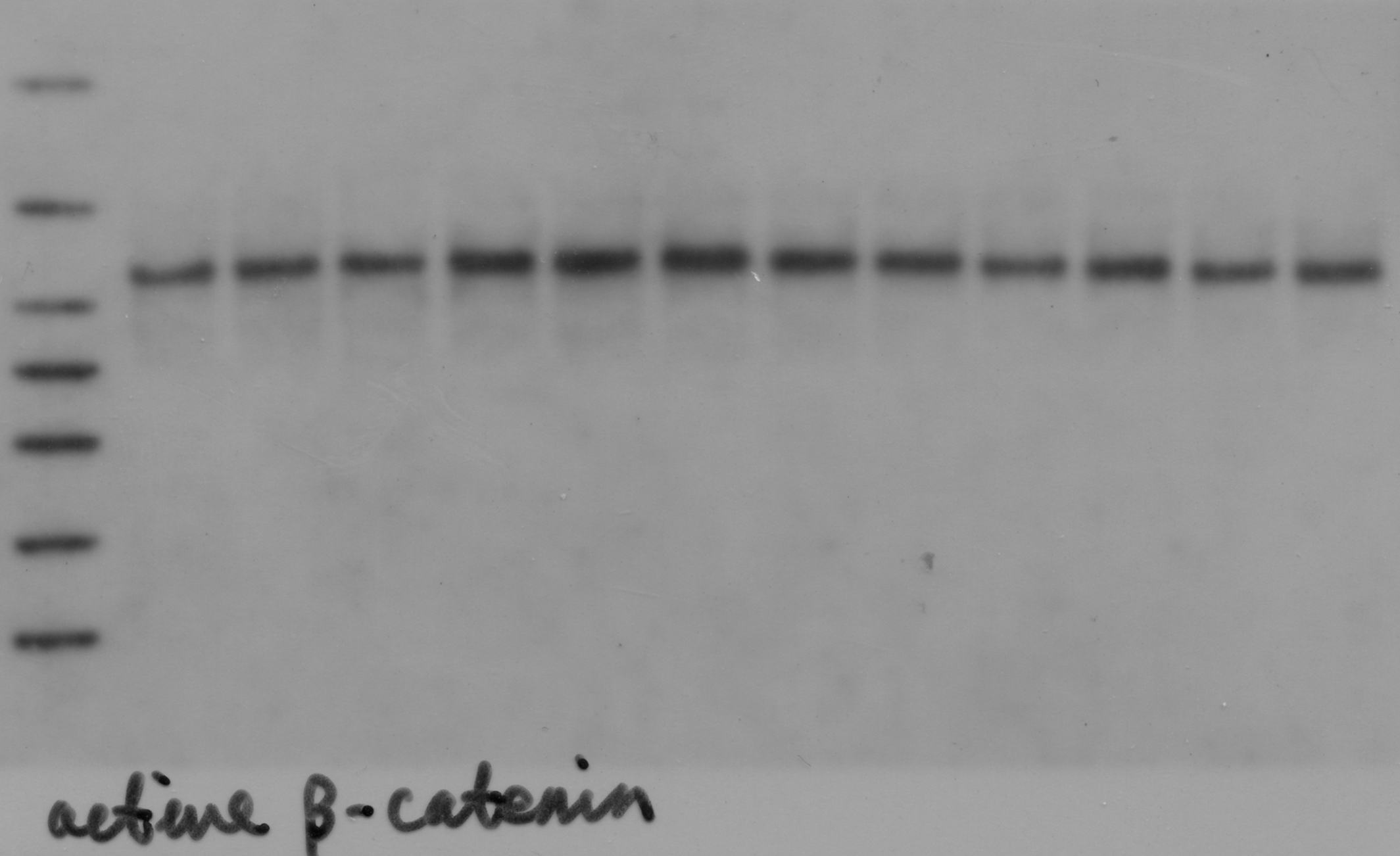

Supplement: Figure 7—source data 2. [file elife-92906-fig7-data2.zip › Figure 7-source data 2/Figure7-Active β-catenin.tif]

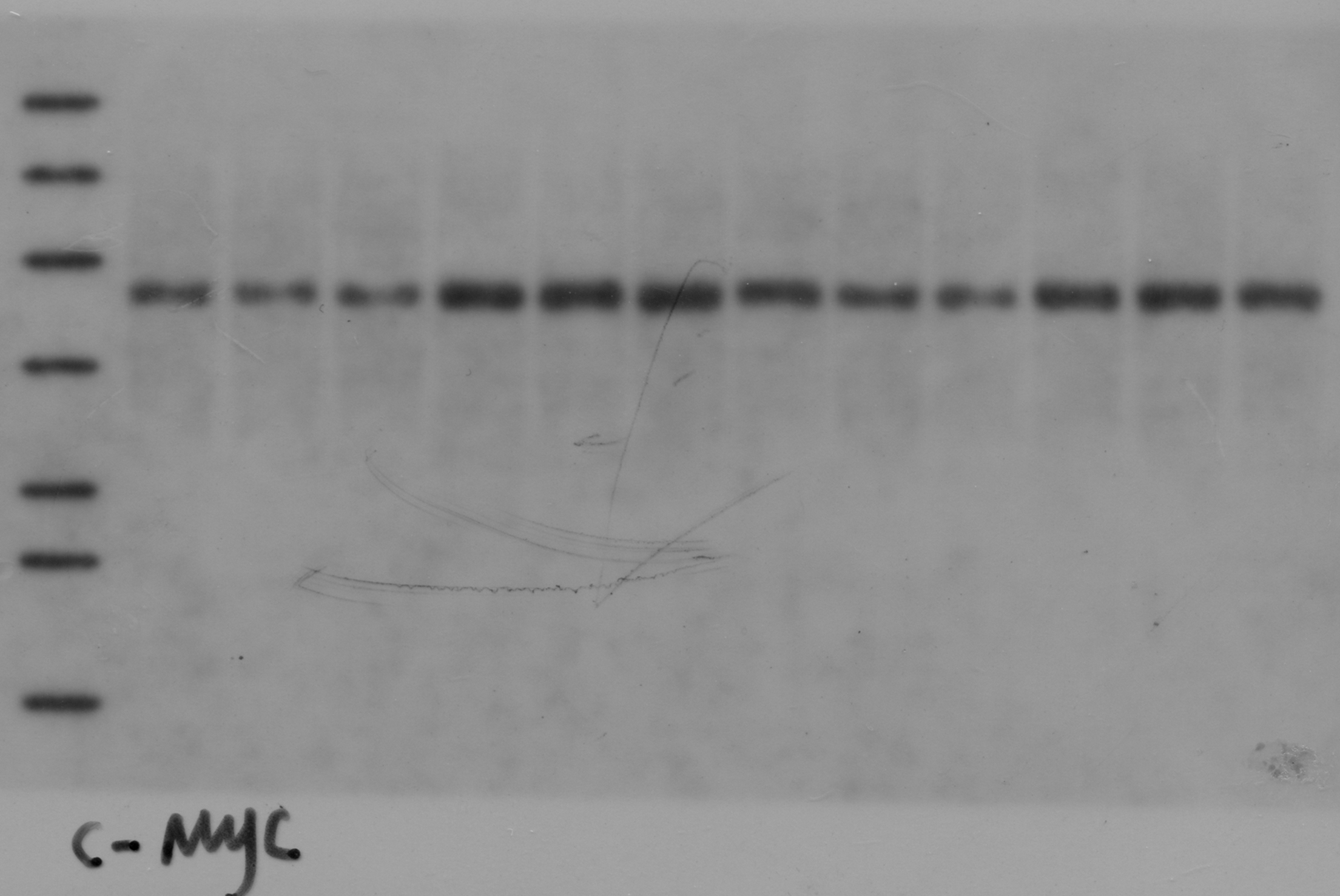

Supplement: Figure 7—source data 2. [file elife-92906-fig7-data2.zip › Figure 7-source data 2/Figure7-C-Myc.tif]

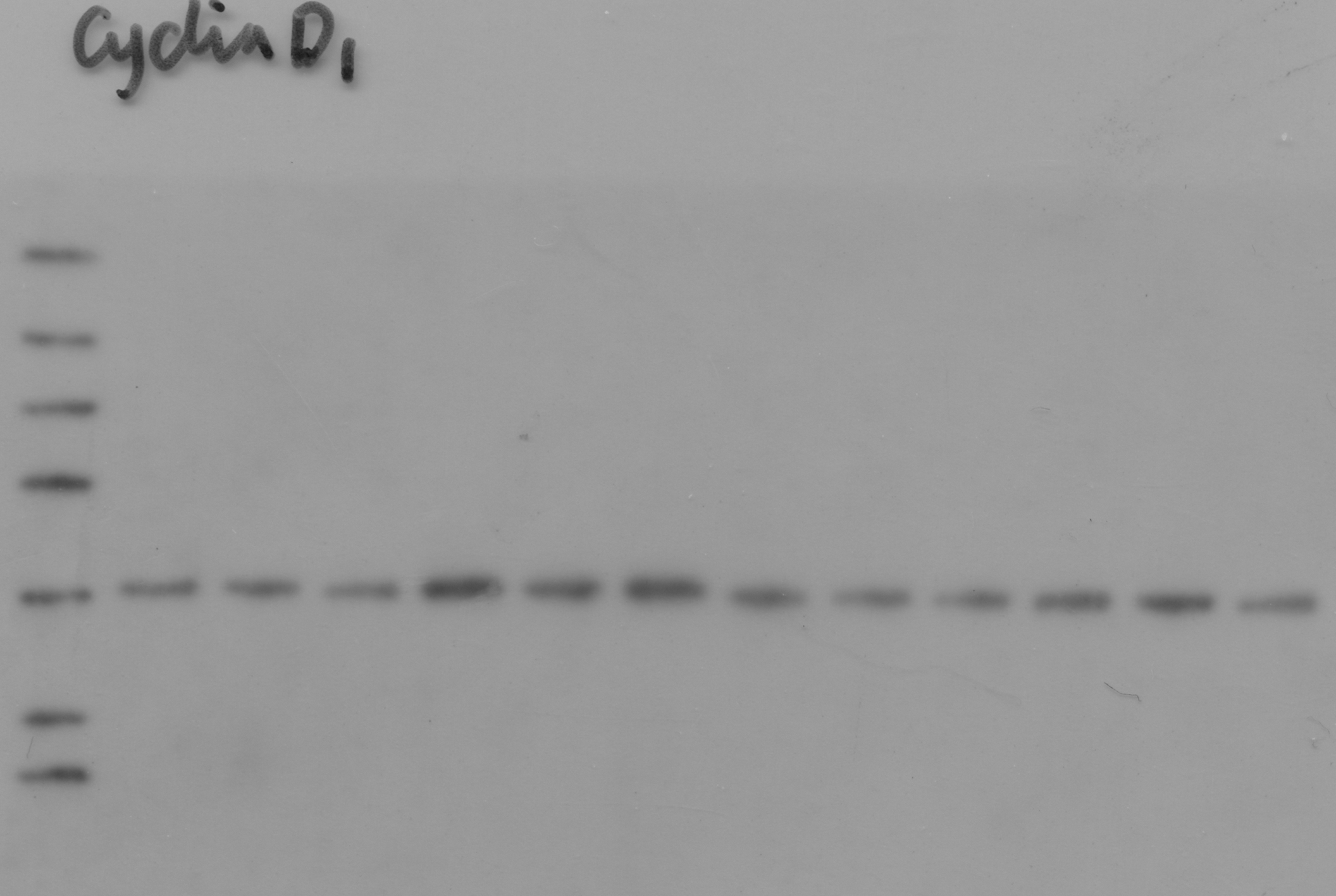

Supplement: Figure 7—source data 2. [file elife-92906-fig7-data2.zip › Figure 7-source data 2/Figure7-CyclinD1.tif]

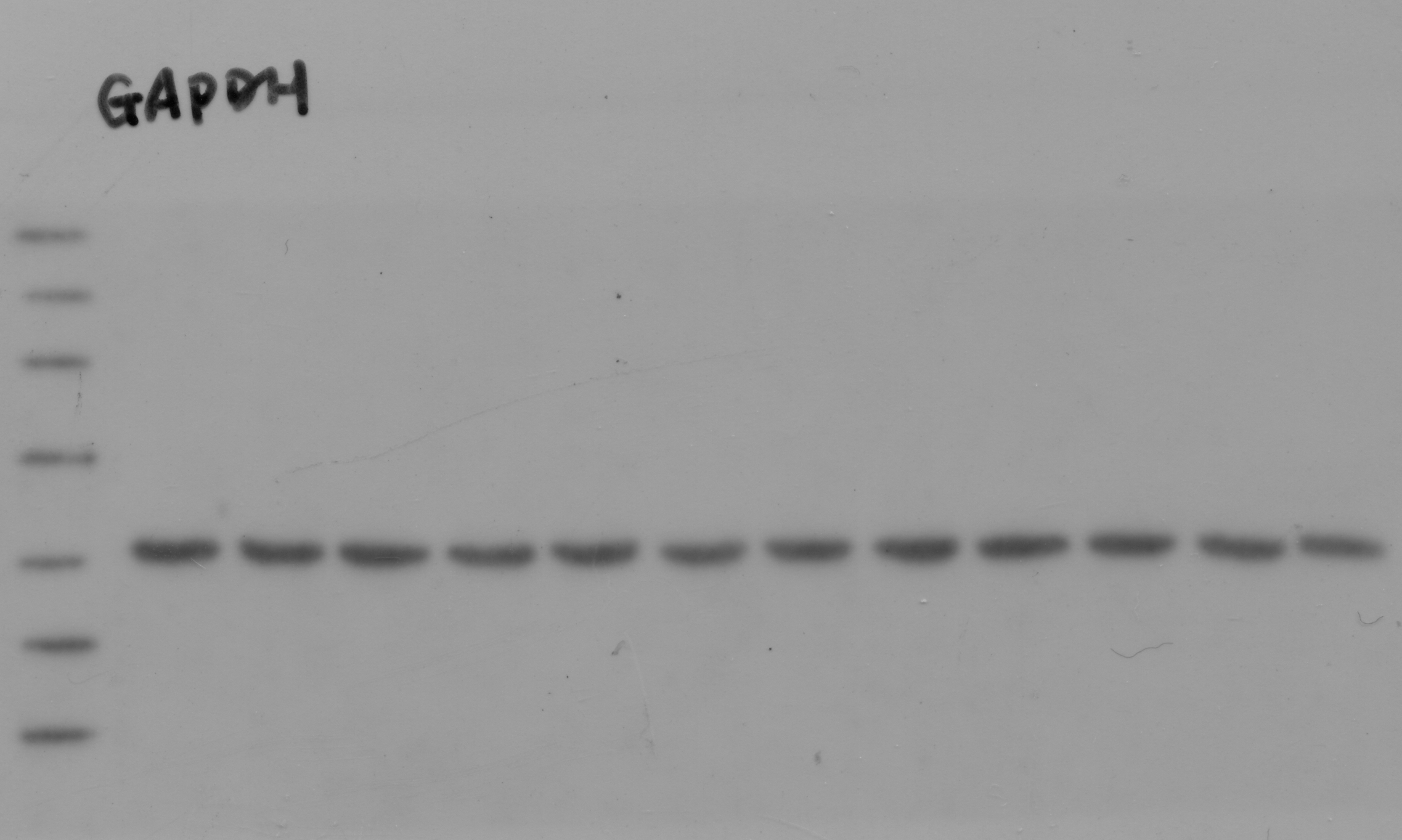

Supplement: Figure 7—source data 2. [file elife-92906-fig7-data2.zip › Figure 7-source data 2/Figure7-GAPDH.tif]

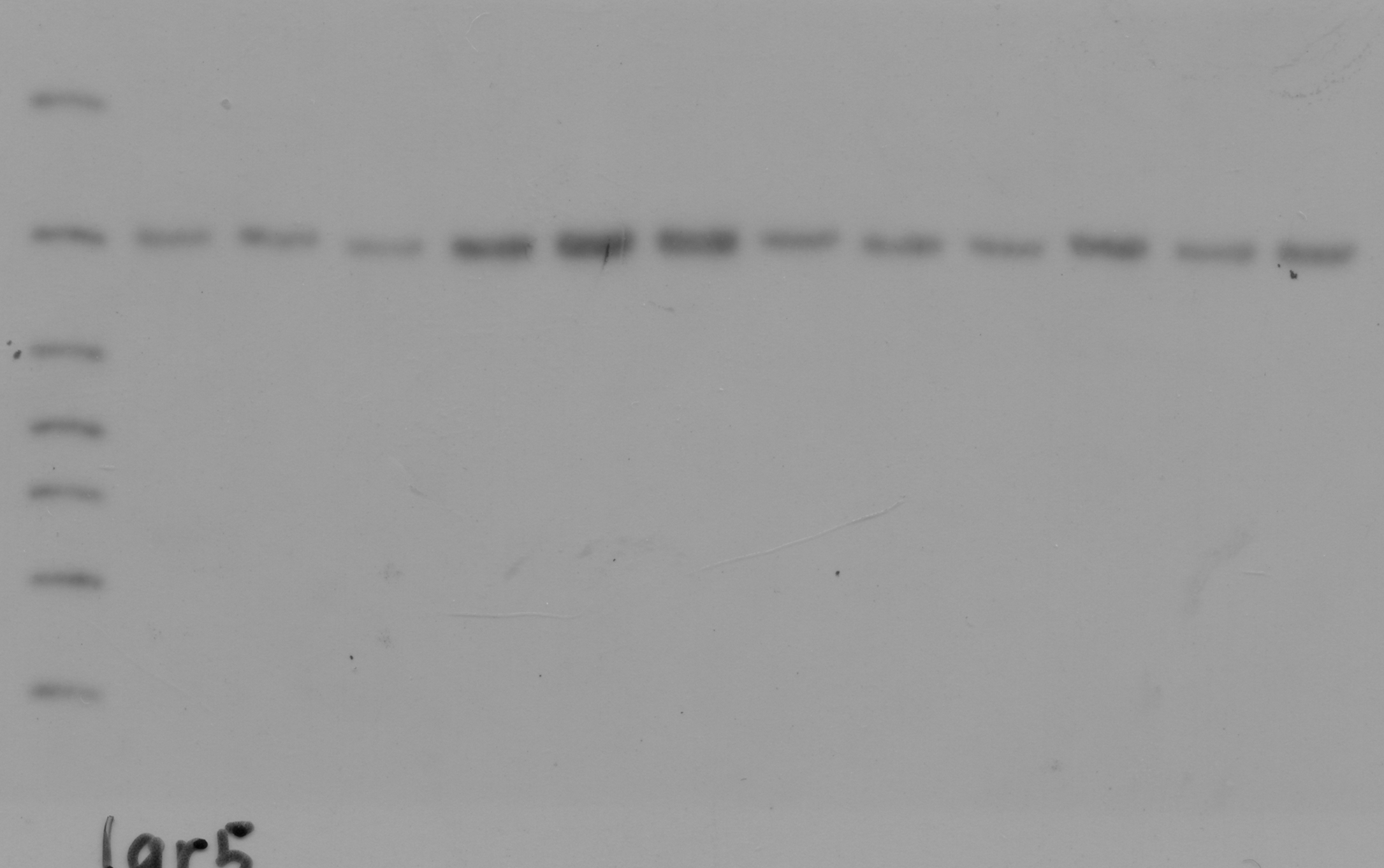

Supplement: Figure 7—source data 2. [file elife-92906-fig7-data2.zip › Figure 7-source data 2/Figure7-Lgr5.tif]

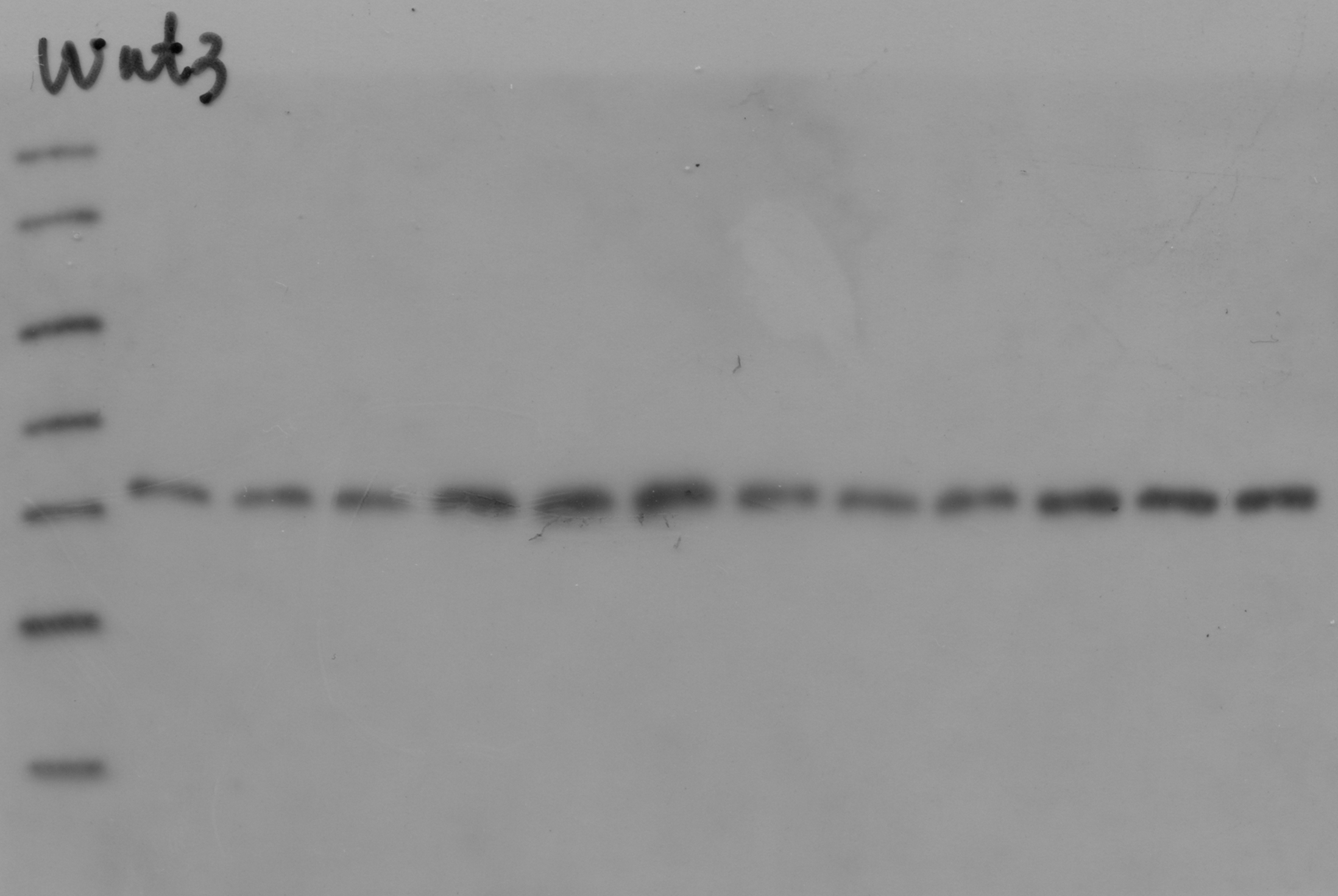

Supplement: Figure 7—source data 2. [file elife-92906-fig7-data2.zip › Figure 7-source data 2/Figure7-Wnt3.tif]

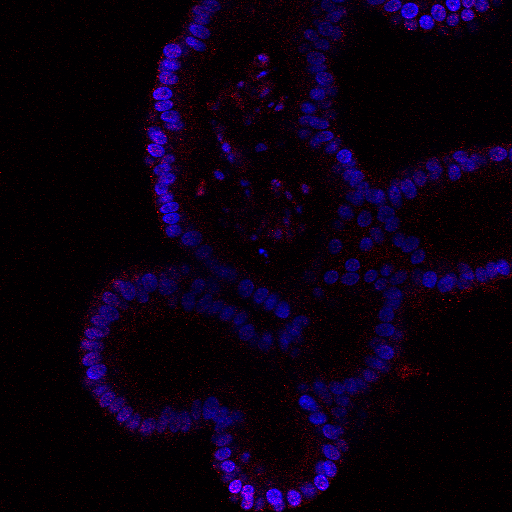

Supplement: Figure 9—source data 1. [file elife-92906-fig9-data1.zip › Figure 9-source data 1/Figur9-ETEC-LGR5.tif]

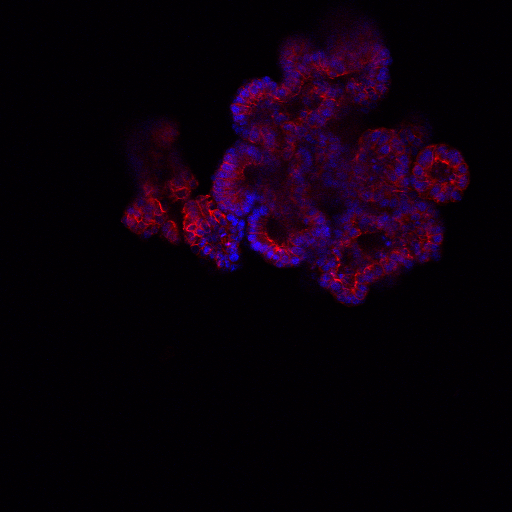

Supplement: Figure 9—source data 1. [file elife-92906-fig9-data1.zip › Figure 9-source data 1/Figure9-AKK-Catenin.tif]

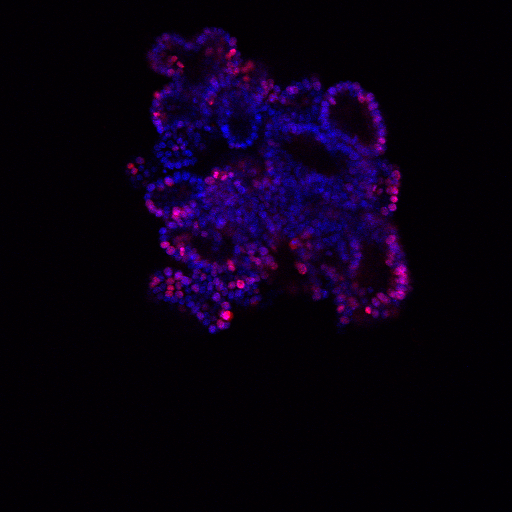

Supplement: Figure 9—source data 1. [file elife-92906-fig9-data1.zip › Figure 9-source data 1/Figure9-AKK-KI67.tif]

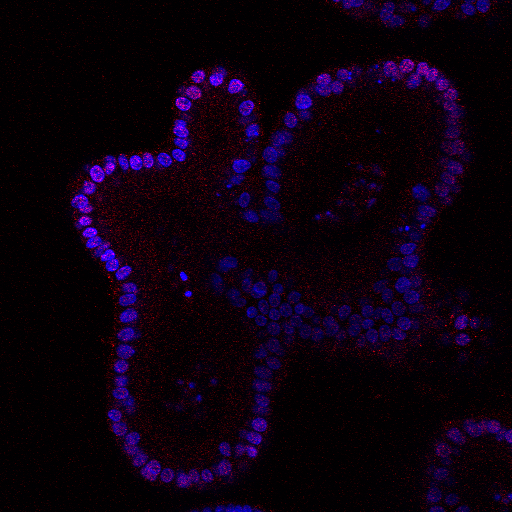

Supplement: Figure 9—source data 1. [file elife-92906-fig9-data1.zip › Figure 9-source data 1/Figure9-AKK-LGR5.tif]

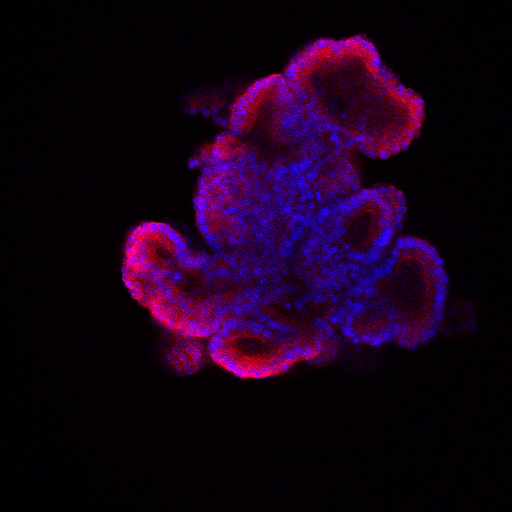

Supplement: Figure 9—source data 1. [file elife-92906-fig9-data1.zip › Figure 9-source data 1/Figure9-AKK-Villin.tif]

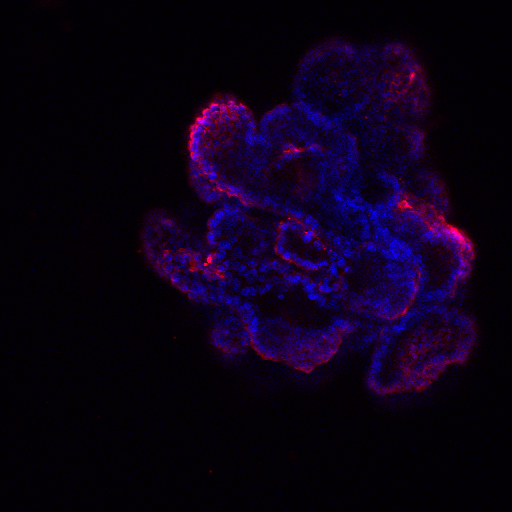

Supplement: Figure 9—source data 1. [file elife-92906-fig9-data1.zip › Figure 9-source data 1/Figure9-AKK-WNT3A.tif]

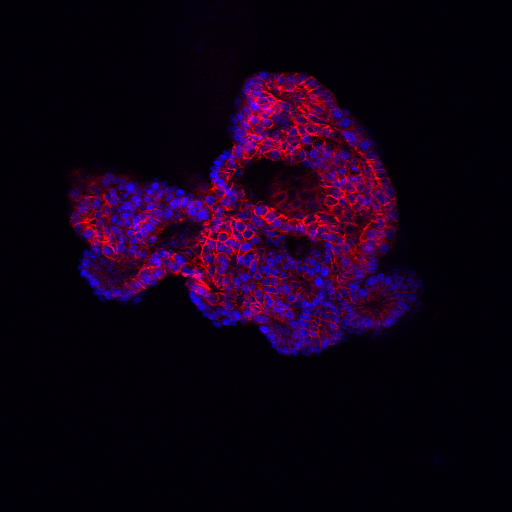

Supplement: Figure 9—source data 1. [file elife-92906-fig9-data1.zip › Figure 9-source data 1/Figure9-CON-Catenin.tif]

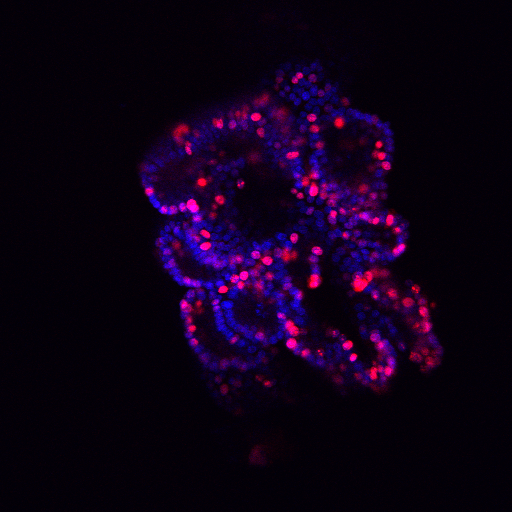

Supplement: Figure 9—source data 1. [file elife-92906-fig9-data1.zip › Figure 9-source data 1/Figure9-CON-KI67.tif]

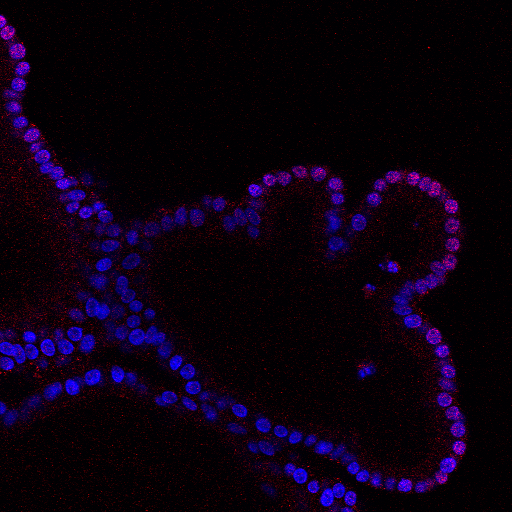

Supplement: Figure 9—source data 1. [file elife-92906-fig9-data1.zip › Figure 9-source data 1/Figure9-CON-LGR5.tif]

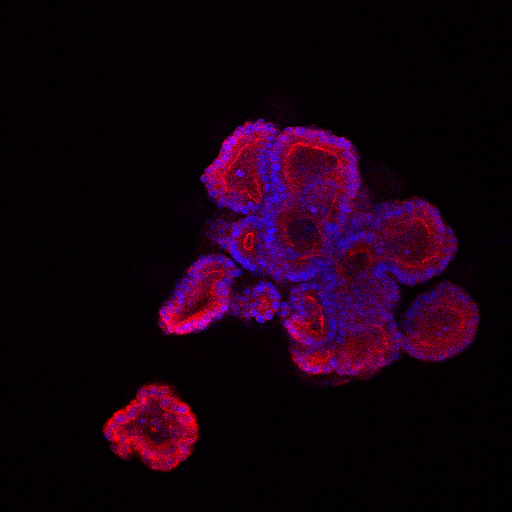

Supplement: Figure 9—source data 1. [file elife-92906-fig9-data1.zip › Figure 9-source data 1/Figure9-CON-Villin.tif]

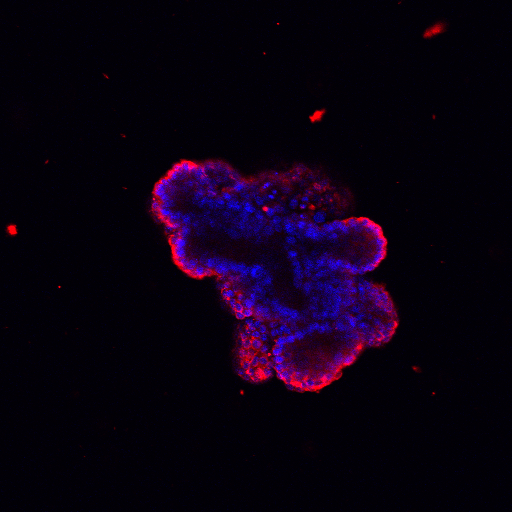

Supplement: Figure 9—source data 1. [file elife-92906-fig9-data1.zip › Figure 9-source data 1/Figure9-CON-WNT3A.tif]

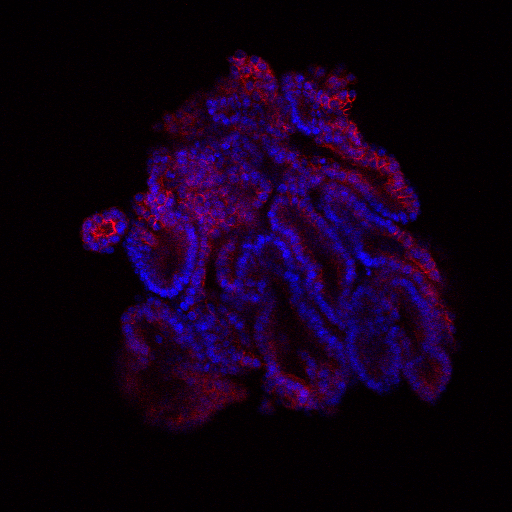

Supplement: Figure 9—source data 1. [file elife-92906-fig9-data1.zip › Figure 9-source data 1/Figure9-ETEC-Catenin.tif]

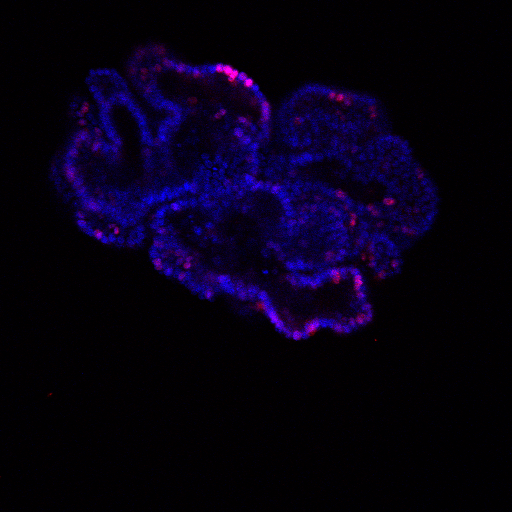

Supplement: Figure 9—source data 1. [file elife-92906-fig9-data1.zip › Figure 9-source data 1/Figure9-ETEC-KI67.tif]

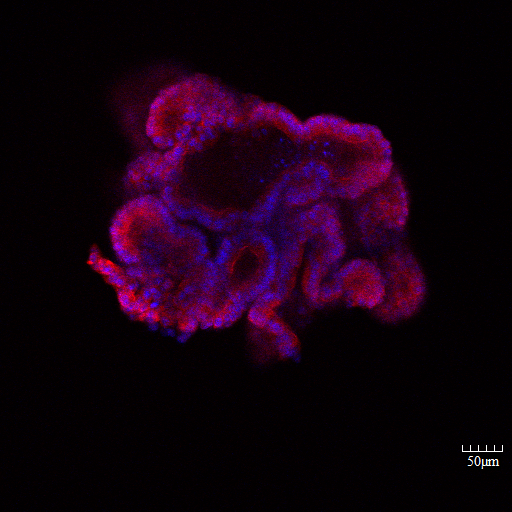

Supplement: Figure 9—source data 1. [file elife-92906-fig9-data1.zip › Figure 9-source data 1/Figure9-ETEC-VILLIN.tif]

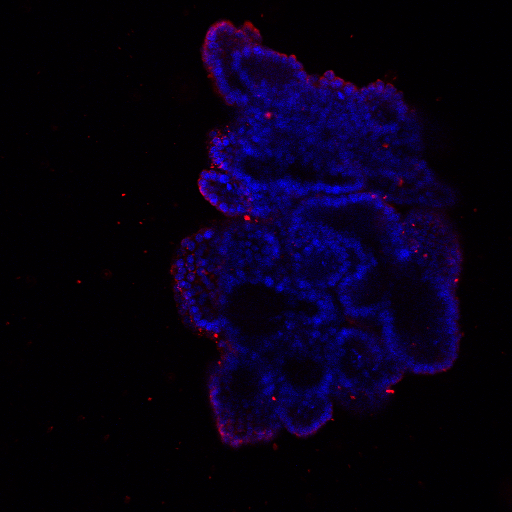

Supplement: Figure 9—source data 1. [file elife-92906-fig9-data1.zip › Figure 9-source data 1/Figure9-ETEC-WNT3A.tif]

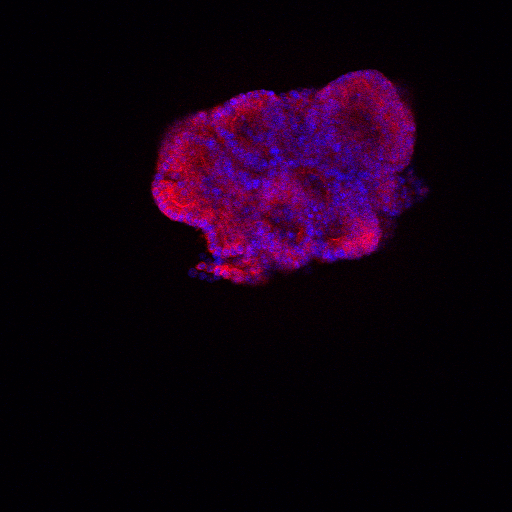

Supplement: Figure 9—source data 1. [file elife-92906-fig9-data1.zip › Figure 9-source data 1/Figure9-WATC59-VILLIN.tif]

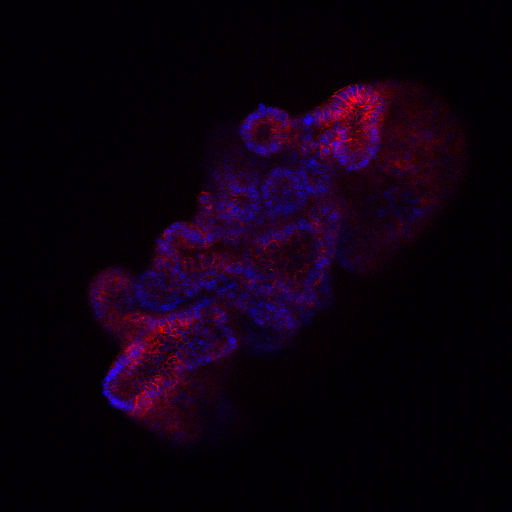

Supplement: Figure 9—source data 1. [file elife-92906-fig9-data1.zip › Figure 9-source data 1/Figure9-WNTC59-Catenin.tif]

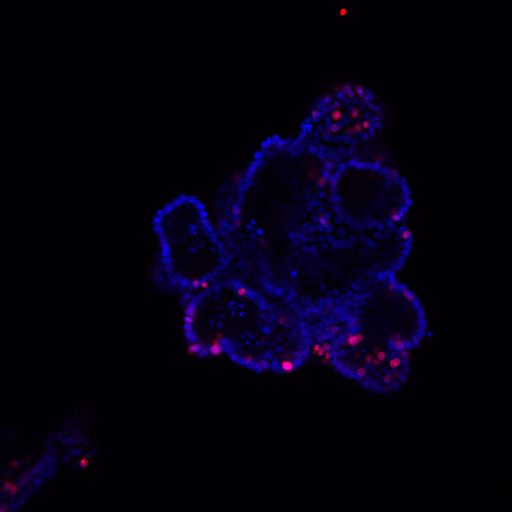

Supplement: Figure 9—source data 1. [file elife-92906-fig9-data1.zip › Figure 9-source data 1/Figure9-WNTC59-KI67.tif]

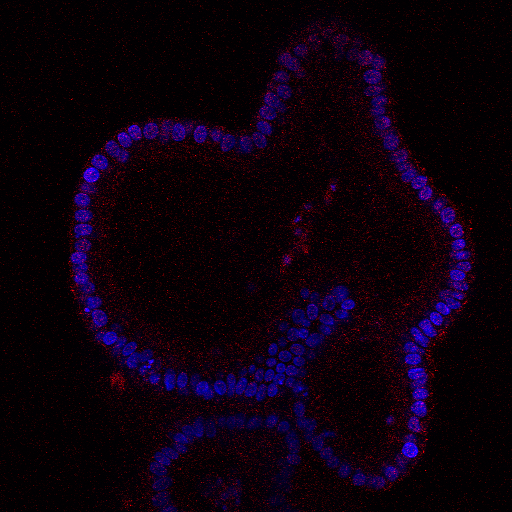

Supplement: Figure 9—source data 1. [file elife-92906-fig9-data1.zip › Figure 9-source data 1/Figure9-WNTC59-LGR5.tif]

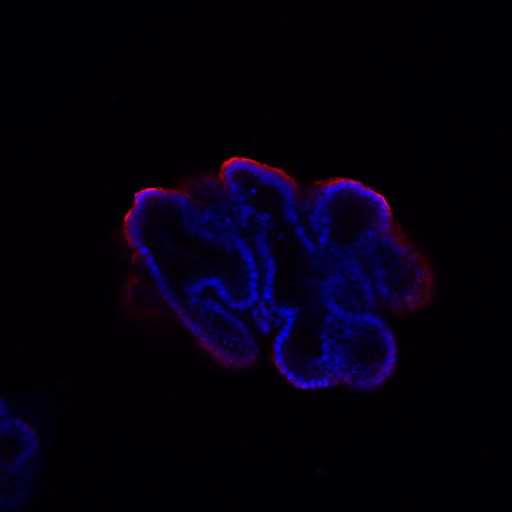

Supplement: Figure 9—source data 1. [file elife-92906-fig9-data1.zip › Figure 9-source data 1/Figure9-WNTC59-WNT3A.tif]

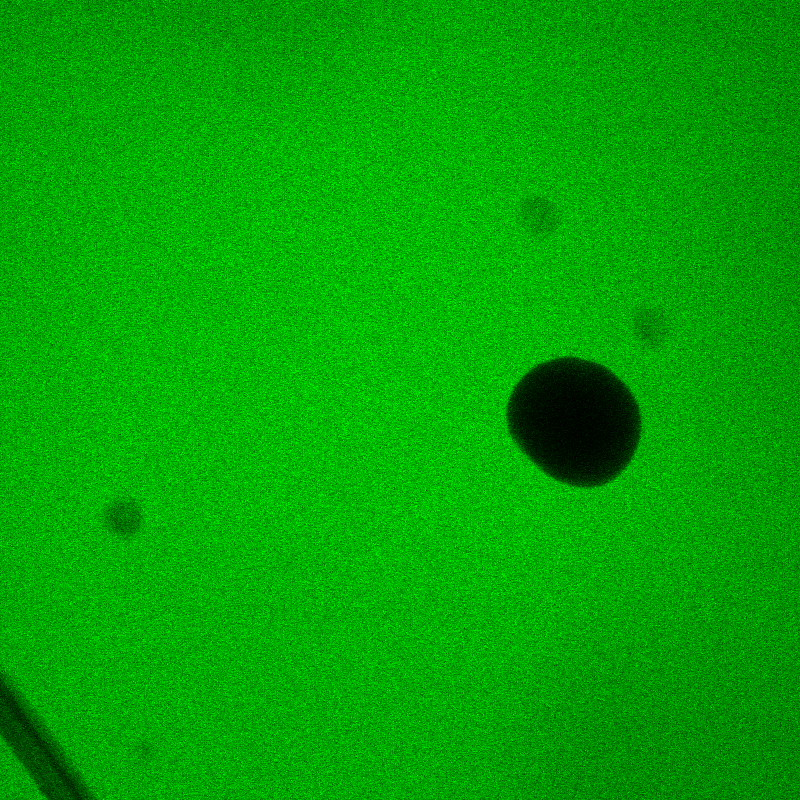

Supplement: Figure 10—source data 1. [file elife-92906-fig10-data1.zip › Figure 10-source data 1/Figure10-AKK-FITC.tif]

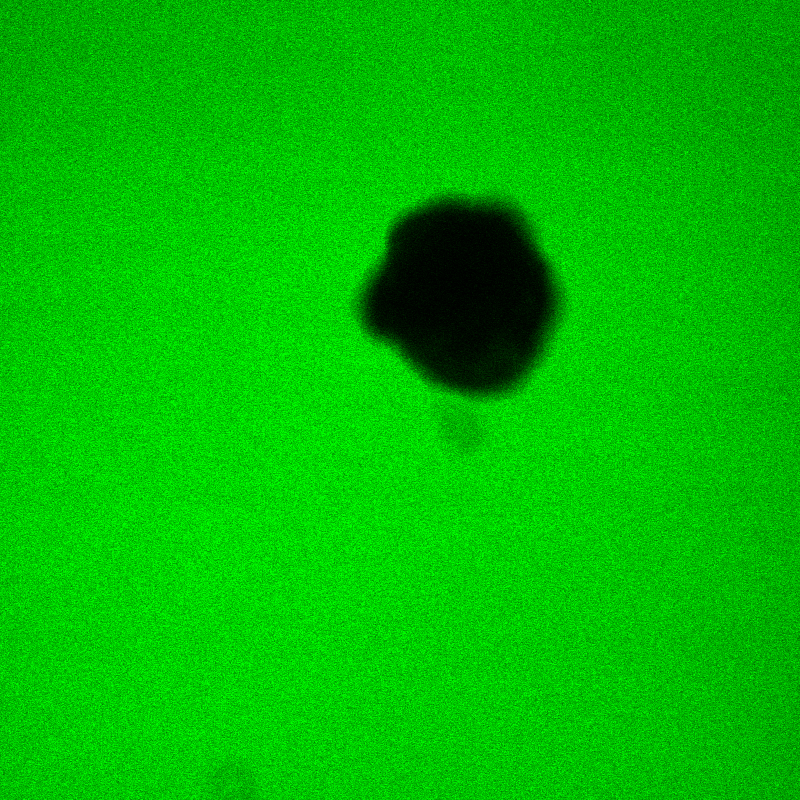

Supplement: Figure 10—source data 1. [file elife-92906-fig10-data1.zip › Figure 10-source data 1/Figure10-CON-FITC.tif]

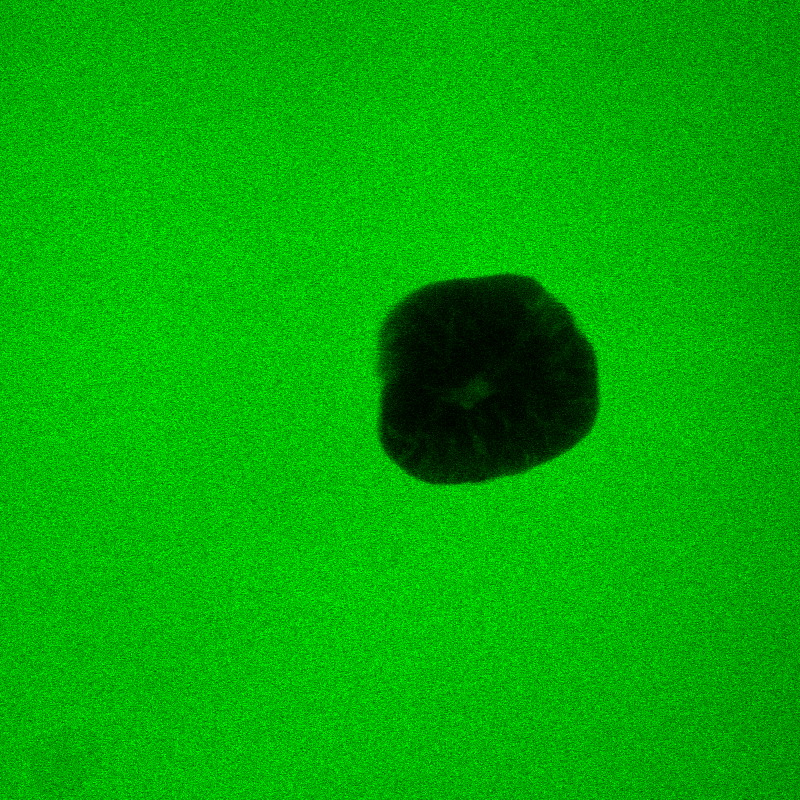

Supplement: Figure 10—source data 1. [file elife-92906-fig10-data1.zip › Figure 10-source data 1/Figure10-ETEC-FITC.tif]

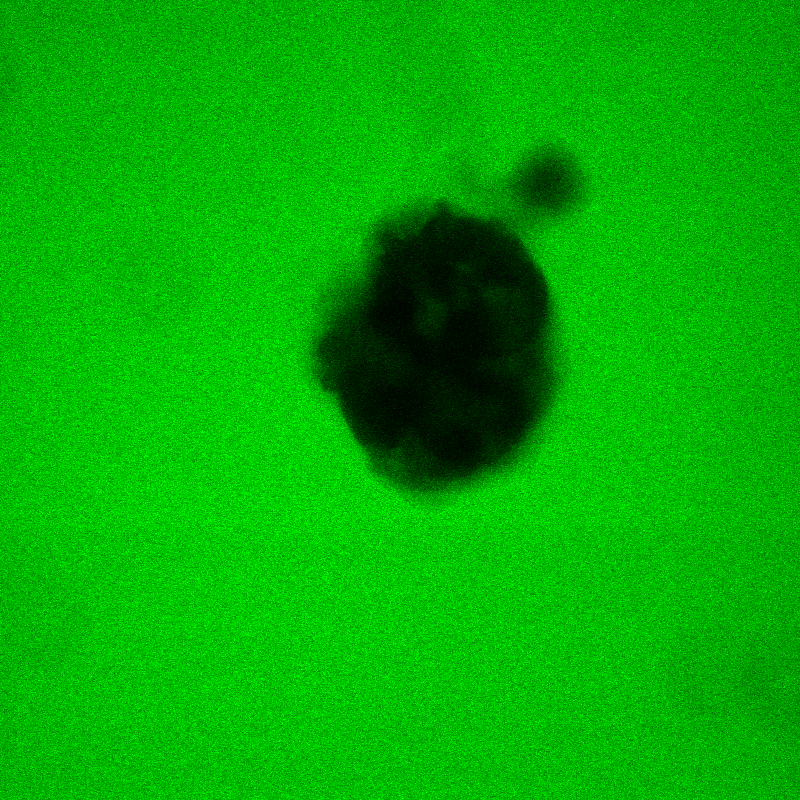

Supplement: Figure 10—source data 1. [file elife-92906-fig10-data1.zip › Figure 10-source data 1/Figure10-WNTC59-FITC.tif]

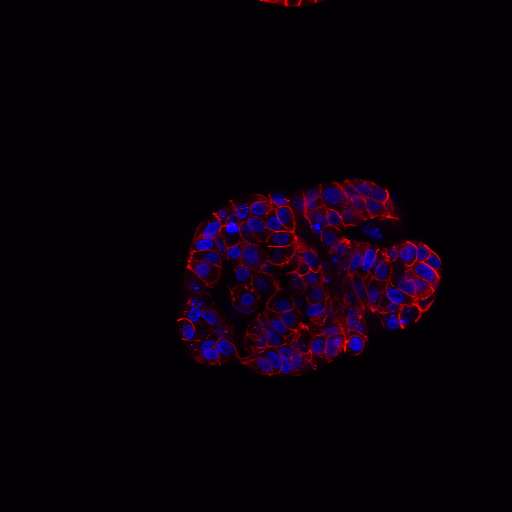

Supplement: Figure 10—source data 2. [file elife-92906-fig10-data2.zip › Figure 10-source data 2/Figure10-AKK-Catenin.tif]

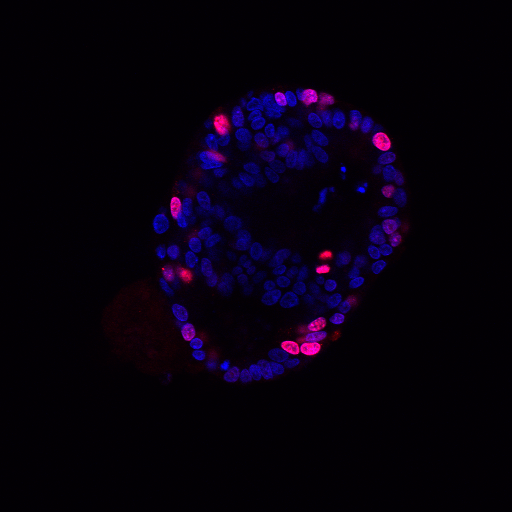

Supplement: Figure 10—source data 2. [file elife-92906-fig10-data2.zip › Figure 10-source data 2/Figure10-AKK-KI67.tif]

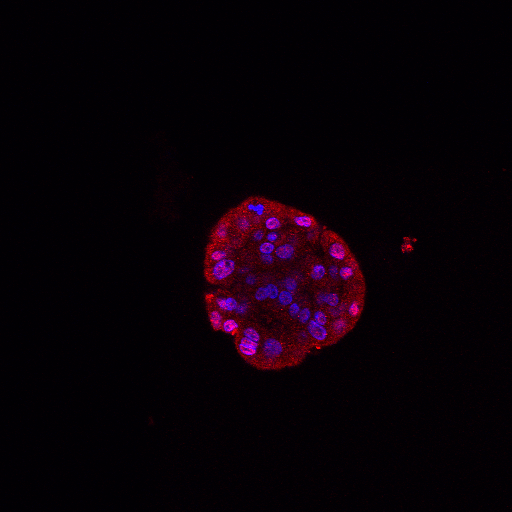

Supplement: Figure 10—source data 2. [file elife-92906-fig10-data2.zip › Figure 10-source data 2/Figure10-Akk-LGR5.tif]

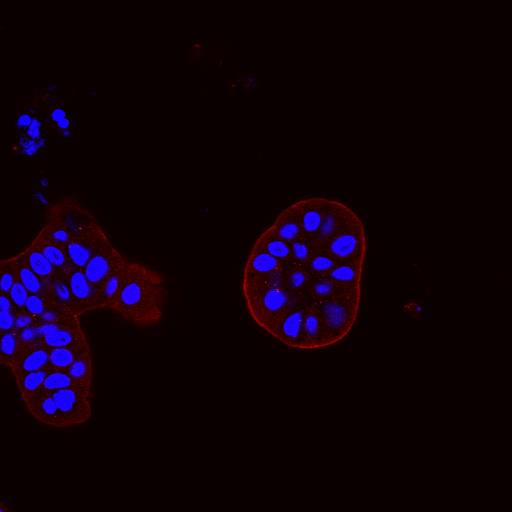

Supplement: Figure 10—source data 2. [file elife-92906-fig10-data2.zip › Figure 10-source data 2/Figure10-AKK-Villin.tif]

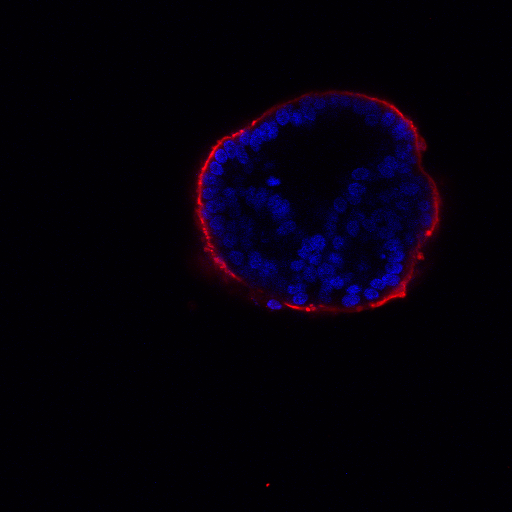

Supplement: Figure 10—source data 2. [file elife-92906-fig10-data2.zip › Figure 10-source data 2/Figure10-Akk-WNT3A.tif]

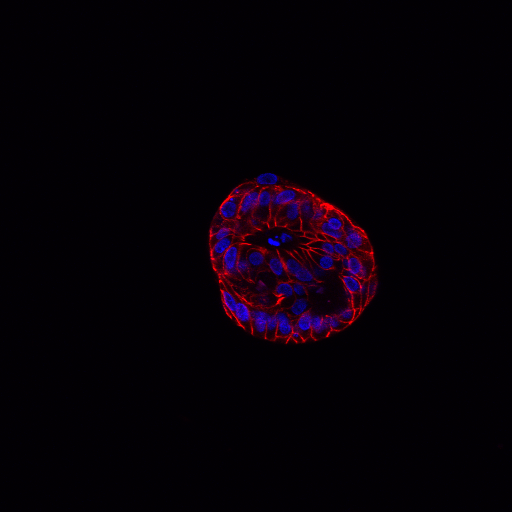

Supplement: Figure 10—source data 2. [file elife-92906-fig10-data2.zip › Figure 10-source data 2/Figure10-CON-Catenin.tif]

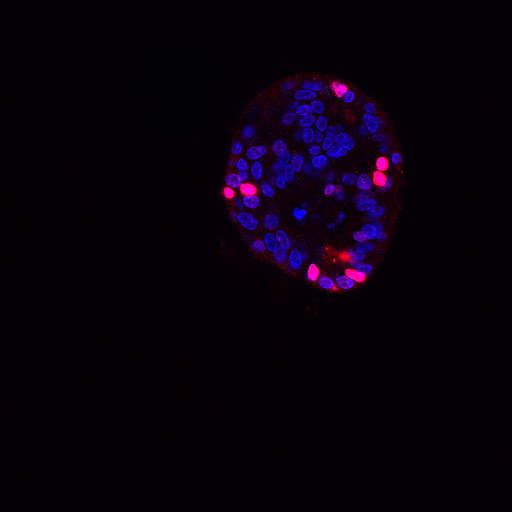

Supplement: Figure 10—source data 2. [file elife-92906-fig10-data2.zip › Figure 10-source data 2/Figure10-CON-KI67.tif]

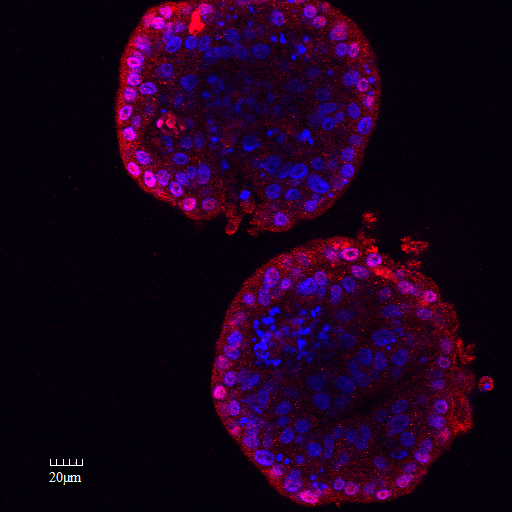

Supplement: Figure 10—source data 2. [file elife-92906-fig10-data2.zip › Figure 10-source data 2/Figure10-CON-LGR5.tif]

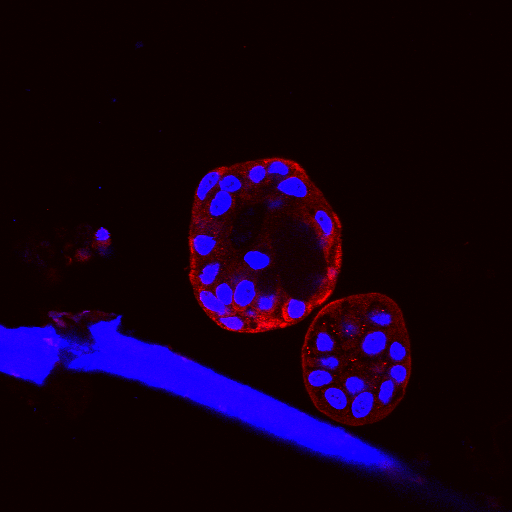

Supplement: Figure 10—source data 2. [file elife-92906-fig10-data2.zip › Figure 10-source data 2/Figure10-CON-Villin.tif]

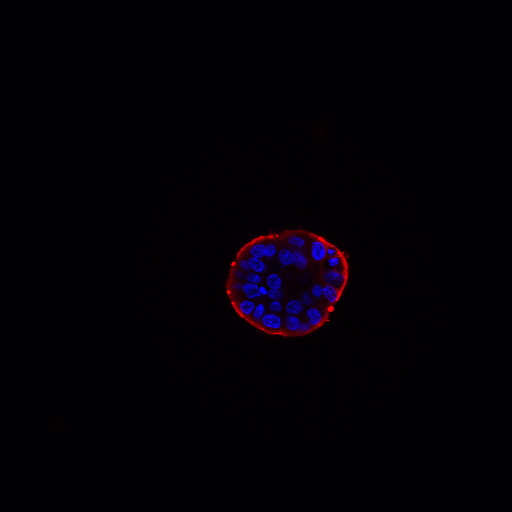

Supplement: Figure 10—source data 2. [file elife-92906-fig10-data2.zip › Figure 10-source data 2/Figure10-CON-WNT3A.tif]

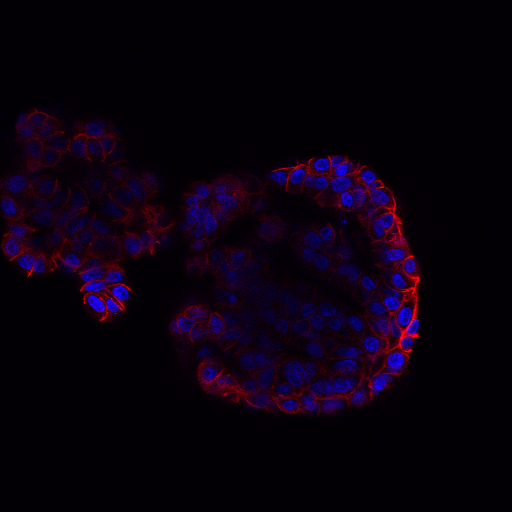

Supplement: Figure 10—source data 2. [file elife-92906-fig10-data2.zip › Figure 10-source data 2/Figure10-ETEC-Catenin.tif]

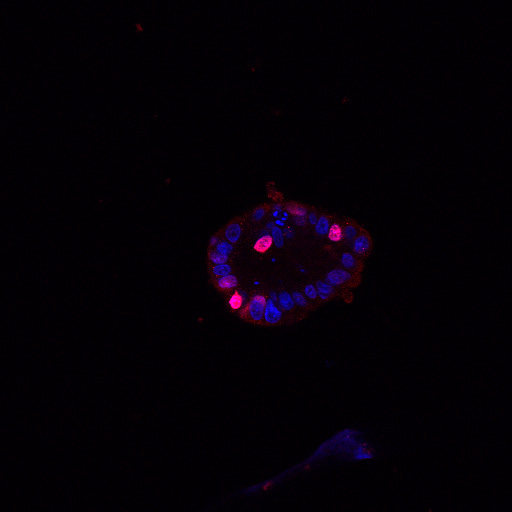

Supplement: Figure 10—source data 2. [file elife-92906-fig10-data2.zip › Figure 10-source data 2/Figure10-ETEC-KI67.tif]

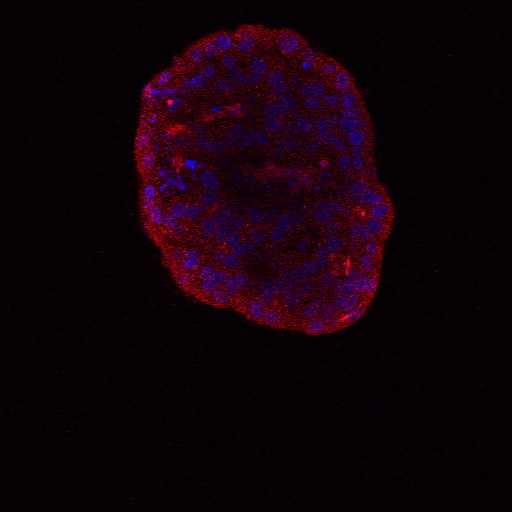

Supplement: Figure 10—source data 2. [file elife-92906-fig10-data2.zip › Figure 10-source data 2/Figure10-ETEC-LGR5.tif]

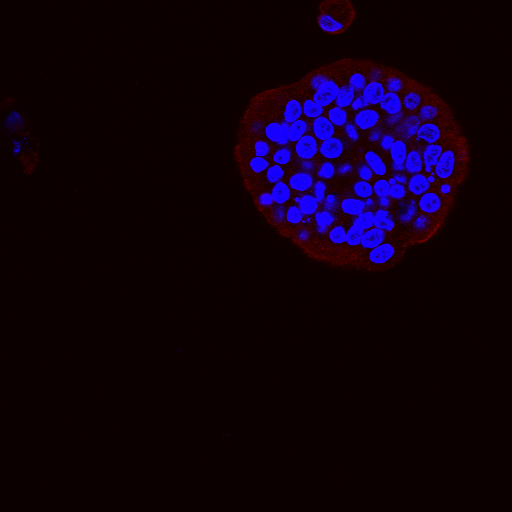

Supplement: Figure 10—source data 2. [file elife-92906-fig10-data2.zip › Figure 10-source data 2/Figure10-ETEC-Villin.tif]

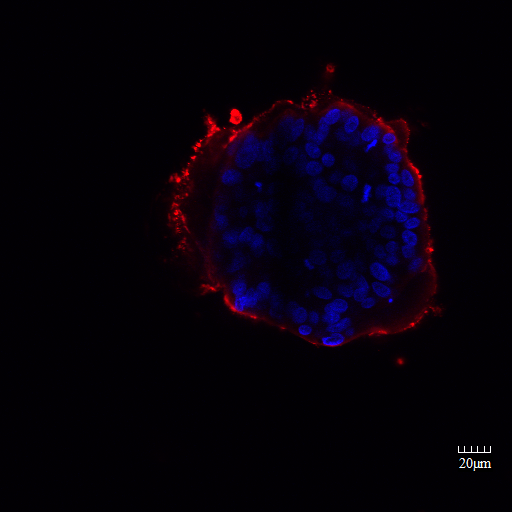

Supplement: Figure 10—source data 2. [file elife-92906-fig10-data2.zip › Figure 10-source data 2/Figure10-ETEC-WNT3A.tif]

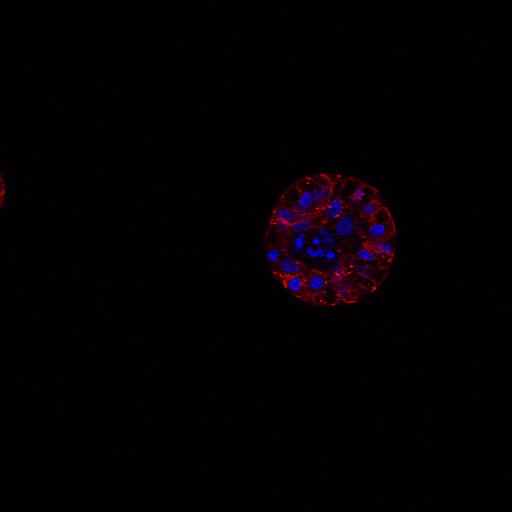

Supplement: Figure 10—source data 2. [file elife-92906-fig10-data2.zip › Figure 10-source data 2/Figure10-WNTC59-Catenin.tif]

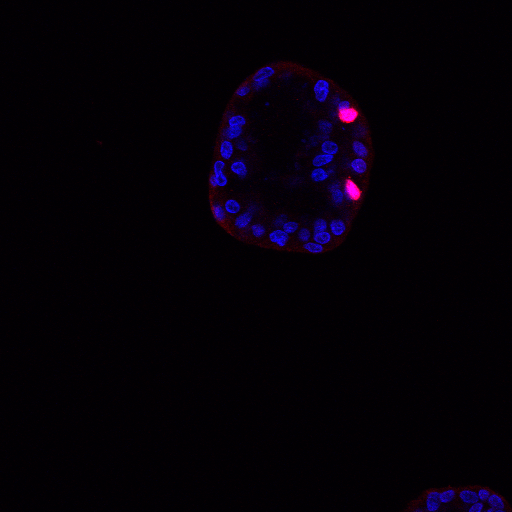

Supplement: Figure 10—source data 2. [file elife-92906-fig10-data2.zip › Figure 10-source data 2/Figure10-WNTC59-KI67.tif]

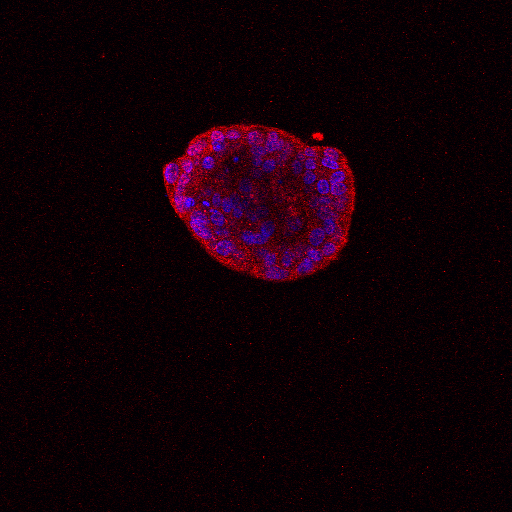

Supplement: Figure 10—source data 2. [file elife-92906-fig10-data2.zip › Figure 10-source data 2/Figure10-WNTC59-LGR5.tif]

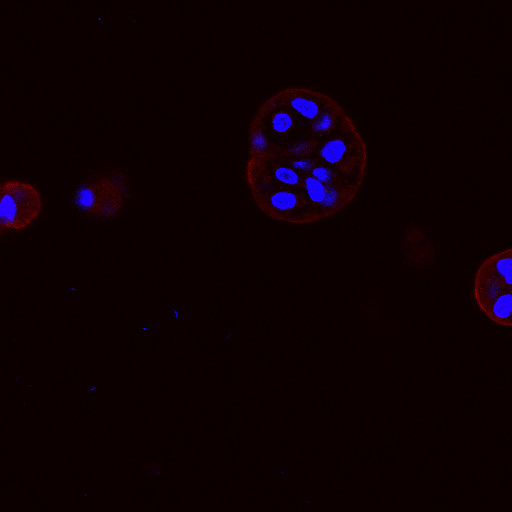

Supplement: Figure 10—source data 2. [file elife-92906-fig10-data2.zip › Figure 10-source data 2/Figure10-WNTC59-Villin.tif]

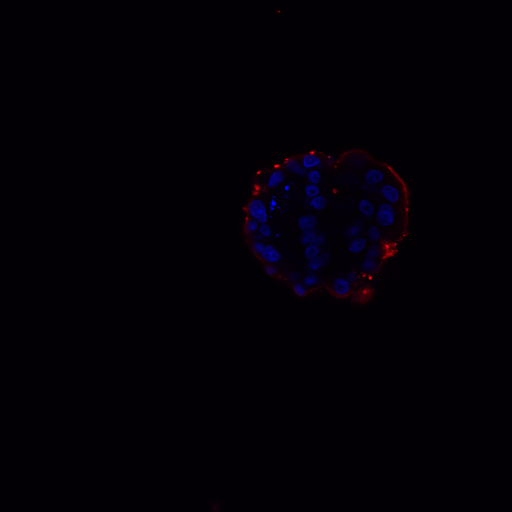

Supplement: Figure 10—source data 2. [file elife-92906-fig10-data2.zip › Figure 10-source data 2/Figure10-WNTC59-WNT3A.tif]
