# Supplementary material for: Akkermansia muciniphila identified as key strain to alleviate gut barrier injury through Wnt signaling pathway
Source: eLife. 2025 Feb 6;12:RP92906. doi: 10.7554/eLife.92906 (PMC11801796; doi:10.7554/eLife.92906)
Supplement: Supplementary file 1. [file elife-92906-supp1.docx]

***Supplementary file 1***

***Akkermansia muciniphila* identified as key strain to alleviate gut barrier injury through Wnt signaling pathway**

Xin Ma ^a,b^, Meng Li ^a,b^, Yuanyuan Zhang ^a^, Tingting Xu ^a^, Xinchen Zhou ^a,b^, Mengqi Qian ^a^, Zhiren Yang ^a,b^, Xinyan Han ^a,b,*^

^a^ Key Laboratory of Animal Nutrition and Feed Science in East China, Ministry of Agriculture, College of Animal Sciences, Zhejiang University, Hangzhou 310058, China

^b^ Hainan Institute of Zhejiang University, Yongyou Industry Park, Yazhou Bay Sci-Tech City, Sanya 572000, China

*Corresponding author

Xinyan Han

College of Animal Sciences, Zhejiang University, 866 Yuhangtang Road, Hangzhou 310058, China

E-mail: [xyhan@zju.edu.cn](mailto:xyhan@zju.edu.cn)

**Supplementary file 1a.** Real-time quantitative PCR primers and conditions.

| Gene | Primer sequences (5' to 3') | Genbank accession / Reference |
| --- | --- | --- |
| ETEC K88 | F: CGCCAGTAACTGGTGGTGTA | M25302.1 |
|  | R: CCATCAGGGTTTCTGAGTACTAC |  |
| Pig-  *IL-10* | F: GACCAGATGGGCGACTTGTTG | NM_214041.1 |
|  | R: GGGAGTTCACGTGCTCCTTGAT |  |
| Pig-  *TGF-β1* | F: GAAGCGCATCGAGGCCATTC | NM_214015 |
|  | R: GGCTCCGGTTCGACACTTTC |  |
| Pig-  *TNF-α* | F: CGCTCTTCTGCCTACTGCACTT | NM_214022.1 |
|  | R: CGGCTTTGACATTGGCTACAA |  |
| Pig-  *IL-1β* | F: GCCAGTCTACATTGCTCATGTTTCT | NM_001005149.1 |
|  | R: GTTGTCACCATTGTTAGCCATCAC |  |
| Pig-  *IL-6* | F: GCCTTCAGTCCAGTCGCCTTCT | NM_214399.1 |
|  | R: GTGGCATCACCTTTGGCATCTTC |  |
| Pig-  *IFN-γ* | F: CAGAGCCAAATTGTCTCCTTCTAC | NM_213948.1 |
|  | R: GTCATTCAGTTTCCCAGAGCTACCA |  |
| Pig-  *ZO-1* | F: GAGGCTCAGCCCTATCCATCTG | XM_021098856.1 |
|  | R: CGGGACCTGCTCATAACTTCGT |  |
| Pig-  *Occludin* | F: CGGCCATATCCAGAGTCTTCGT | NM_001163647.2 |
|  | R: CGTTTTGAAGACGCCTCCAAGT |  |
| Pig-  *Claudin1* | F: CCTACGCTGGTGACAACATTG | NM_001244539.1 |
|  | R: GTGGTGTTCAGATTCAGCAAGGA |  |
| Pig-  *E-Cadherin* | F: CCCCAACACTTCTCCCTTCACT | EU805482.1 |
|  | R: CTCGAGGGTTTTCTTTGGCTTC |  |
| Pig-  *β-catenin* | F: GCTGCTGTTTTGTTCCGAATGTC | NM_214367.1 |
|  | R: CCTGGGCACCAATATCAAGTCC |  |
| Pig-  *Lgr5* | F: CCTTGGCCCTGAACAAAATA | Gonzalez et al., 2013 |
|  | R: ATTTCTTTCCCAGGGAGTGG |  |
| Pig-  *Ki67* | F: AGTCTGTAAGGAAAGCCACCC | Stenhouse et al., 2018 |
|  | R: ACAAAGCCCAAGCAGACAGG |  |
| Pig-  *Lyz1* | F: GGTCTATGATCGGTGCGAGT | Gonzalez et al., 2013 |
|  | R: AACTGCTTTGGGTGTCTTGC |  |
| Pig-  *MUC2* | F: GGCTGCTCATTGAGAGGAGT | Gonzalez et al., 2013 |
|  | R: ATGTTCCCGAACTCCAAGG |  |
| Pig-  *villin* | F: ACGTGTCTGACTCCGAGGGAAAGGT | Yin et al., 2022 |
|  | R: ACTGCTTCGCTTTGATAAAGTTCAG |  |
| Pig-  *Wnt3a* | F: GCGACTTCCTCAAGGACAAG | Gonzalez et al., 2013 |
|  | R: GGTCACGTGTACCGAAGGAT |  |
| Pig-  *β-actin* | F: CACGCCATCCTGCGTCTGGA | Krishna et al., 2015 |
|  | R: AGCACCGTGTTGGCGTAGAG |  |
| Mouse-  *IL-10* | F: GGACCAGCTGGACAACATACTGCTA | Yu et al., 2020 |
|  | R: CCGATAAGGCTTGGCAACCCAAGT |  |
| Mouse-  *TGF-β1* | F: GCTGAACCAAGGAGACGGAAT | Ran et al., 2020 |
|  | R: GCTGATCCCGTTGATTTCCA |  |
| Mouse-  *TNF-α* | F: CCACGCTCTTCTGTCTACTG | Yu et al., 2020 |
|  | R: ACTTGGTGGTTTGCTACGAC |  |
| Mouse-  *IL-1β* | F: GGACAGCCTGTTACTACCTGACACATT | Ding et al., 2020 |
|  | R: CCTAGGAAACAGCAATGGTCGGGAC |  |
| Mouse-  *IL-6* | F: GAGTCACAGAAGGAGTGGCTAAGGA | Yu et al., 2020 |
|  | R: CGCACTAGGTTTGCCGAGTAGATCT |  |
| Mouse-  *IFN-γ* | F: GGACCAGCTGGACAACATACTGCTA | Yu et al., 2020 |
|  | R: CCGATAAGGCTTGGCAACCCAAGT |  |
| Mouse-  *Axin2* | F: AACCTATGCCCGTTTCCTCT | Kim et al., 2021 |
|  | R: GAGTGTAAAGACTTGGTCCA |  |
| Mouse-  *Ctnnb1* | F: ATGGAGCCGGACAGAAAAGC | Kim et al., 2021 |
|  | R: GAGTGTAAAGACTTGGTCCA |  |
| Mouse-  *Hes1* | F: CCAGCCAGTGTCAACACGA | Kim et al., 2021 |
|  | R: AATGCCGGGAGCTACTTTCT |  |
| Mouse-  *Ki67* | F: CCAGCTGCCTGTAGTGTCAA | Sittipo et al., 2020 |
|  | R: TCTTGAGGCTCGCCTTGATG |  |
| Mouse-  *Lgr5* | F: CCTGTCCAGGCTTTCAGAAG | Kim et al., 2021 |
|  | R: CTGTGGAGTCCATCAAAGCA |  |
| Mouse-  *Lyz1* | F: ATGGCGAACACAATGTCAAA | Kim et al., 2021 |
|  | R: GCCCTGTTTCTGCTGAAGTC |  |
| Mouse-  *MUC2* | F: CCTTAGCCAAGGGCTCGGAA | Kim et al., 2021 |
|  | R: GGCCCGAGAGTAGACCTTGG |  |
| Mouse-  *Notch1* | F: GCTGCCTCTTTGATGGCTTCGA | Kim et al., 2021 |
|  | R: CACATTCGGCACTGTTACAGCC |  |
| Mouse-  *Wnt3* | F: CTTCTAATGGAGCCCCACCT | Kim et al., 2021 |
|  | R: GAGGCCAGAGATGTGTACTGC |  |
| Mouse-  *β-actin* | F: TGGAATCCTGTGGCATCCATGAAAC | Kim et al., 2021 |
|  | R: TAAAACGCAGCTCAGTAACAGTCCG |  |

**Supplementary file 1b.** The information of antibodies used in Immunofluorescence

| Antibody | Company | Catalog | Host species | Dilution |
| --- | --- | --- | --- | --- |
| Pig-β-catenin | Servicebio | GB12016 | mouse | 1:1000 |
| Pig-Ki67 | Servicebio | GB121141 | mouse | 1:300 |
| Pig-Lgr5 | Origene technologies | TA503316 | mouse | 1:100 |
| Pig-MUC2 | Servicebio | GB14110 | mouse | 1:500 |
| Pig-villin | Servicebio | GB121209 | mouse | 1:500 |
| Pig-Wnt3a | Thermo Fisher Scientific | MA-31954 | mouse | 1:200 |
| Mouse-Ki67 | Servicebio | GB111141 | rabbit | 1:500 |
| Mouse-Lyz | Servicebio | GB11345 | rabbit | 1:500 |
| Mouse-MUC2 | Servicebio | GB11344 | rabbit | 1:500 |
| Phalloidine | Servicebio | G1028 |  | 1:300 |
| iFluor^TM^ 594 conjugated Goat anti-mouse IgG | Huabio | HA1126 | mouse | 1:500 |
| Alexa Fluor^®^ 488 conjugated Goat anti-mouse IgG (H+L) | Servicebio | GB25301 | mouse | 1:500 |
| Alexa Fluor^®^ 488 conjugated Goat anti-rabbit IgG (H+L) | Servicebio | GB25303 | rabbit | 1:500 |
| Cy3 conjugated Goat anti-rabbit IgG (H+L) | Servicebio | GB21303 | rabbit | 1:500 |

**Supplementary file 1c.** The information of antibodies used in Western blot

| Antibody | Company | Catalog | Host Species | Dilution |
| --- | --- | --- | --- | --- |
| Pig-ZO-1 | Thermo Fisher Scientific | 40-2200 | rabbit | 1:500 |
| Pig-Occludin | Abcam | ab222691 | rabbit | 1:500 |
| Pig-Claudin1 | Abcam | ab129119 | rabbit | 1:1000 |
| Pig-E-Cadherin | Thermo Fisher Scientific | 13-1700 | mouse | 1:1000 |
| Pig-β-catenin | Abcam | ab16051 | rabbit | 1:4000 |
| Mouse-Active-β-catenin | Cell Signaling Technology | #19807 | rabbit | 1:1000 |
| Mouse-c-Myc | Cell Signaling Technology | #18583 | rabbit | 1:1000 |
| Mouse-CyclinD1 | Cell Signaling Technology | #55506 | rabbit | 1:2000 |
| Mouse-Lgr5 | Abcam | ab75850 | rabbit | 1:1000 |
| Mouse-Wnt3a | Abcam | ab75850 | rabbit | 1:500 |
| GAPDH | Abcam | ab181602 | rabbit | 1:10000 |
| Goat anti-rabbit IgG (H+L) | Thermo Fisher Scientific | 31210 | rabbit | 1:5000 |
| Goat anti-mouse IgG (H+L) | Thermo Fisher Scientific | 31431 | mouse | 1:5000 |

**Supplementary file 1d.** The information of antibodies used in Flow cytometry

| Antibody | Company | Catalog |
| --- | --- | --- |
| Fixable Viability Stain 510 | BD Pharmingen | 564406 |
| Ms CD45 PerCP-Cy5.5 30-F11 | BD Pharmingen | 550994 |
| Ms CD3e BV786 145-2C11 | BD Pharmingen | 564379 |
| Ms CD4 FITC RM4-5 | BD Pharmingen | 553046 |
| Ms CD8a APC-Cy7 53-6.7 | BD Pharmingen | 557654 |
| Ms CD25 PE 3C7 | BD Pharmingen | 553075 |
| Ms Foxp3 Alexa 647 MF23 | BD Pharmingen | 560401 |
| Ms ROR Gma T BV650 Q31-378 | BD Pharmingen | 564722 |
| Ms I-A I-E BV605 M5/114.15.2 | BD Pharmingen | 563413 |
| Ms CD11c BV421 HL3 | BD Pharmingen | 562782 |
| Ms CD86 PE-Cy7 GL1 | BD Pharmingen | 560582 |
| Ms CD103 R718 M290 | BD Pharmingen | 752271 |

**REFERENCES**

Ding XL, Yu HT, Qiao SY. Lasso Peptide Microcin J25 Effectively Enhances Gut Barrier Function and Modulates Inflammatory Response in an Enterotoxigenic *Escherichia coli*-Challenged Mouse Model. Int. J. Mol. Sci. 2020, 21(18).

Kim S, Shin YC, Kim TY, Kim Y, Lee YS, Lee SH, Kim MN, Eunju O, Kim KS, Kweon MN. Mucin degrader *Akkermansia muciniphila* accelerates intestinal stem cell-mediated epithelial development. Gut Microbes. 2021, 13(1).

Krishna VD, Roach E, Zaidman NA, Panoskaltsis-Mortari A, Rotschafer JH, O'Grady SM, Cheeran MCJ. Differential Induction of Type I and Type III Interferons by Swine and Human Origin H1N1 Influenza A Viruses in Porcine Airway Epithelial Cells. PLoS One. 2015, 10(9).

Gonzalez LM, Williamson I, Piedrahita JA, Blikslager AT, Magness ST. Cell Lineage Identification and Stem Cell Culture in a Porcine Model for the Study of Intestinal Epithelial Regeneration. PLoS One. 2013, 8(6).

Sittipo P, Pham HQ, Park CE, Kang GU, Zhi Y, Ji HJ, Jang A, Seo HS, Shin JH, Lee YK. Irradiation-Induced Intestinal Damage Is Recovered by the Indigenous Gut Bacteria *Lactobacillus acidophilus*. Front. Cell. Infect. Microbiol. 2020, 10.

Stenhouse C, Hogg CO, Ashworth CJ. Associations between fetal size, sex and both proliferation and apoptosis at the porcine feto-maternal interface. Placenta. 2018, 70: 15-24.

Yin LM, Li J, Zhang YT, Yang Q, Yang CY, Yi ZF, Yin YB, Wang QY, Li JZ, Ding NS, Zhang ZG, Yang HS, Yin YL. Changes in progenitors and differentiated epithelial cells of neonatal piglets. Anim. Nutr. 2022, 8(1): 265-276.

Yu HT, Wang YM, Zeng XF, Cai S, Wang G, Liu L, Huang S, Li N, Liu HB, Ding XL, Song QL, Qiao SY. Therapeutic administration of the recombinant antimicrobial peptide microcin J25 effectively enhances host defenses against gut inflammation and epithelial barrier injury induced by enterotoxigenic Escherichia coli infection. Faseb J. 2020, 34(1): 1018-1037.
